# A quantitative definition of hypervalency†
†Electronic supplementary information (ESI) available: Full data for charge calculations on the test set of molecules; original data for Fig. 1–3, including geometries, energies and QTAIM charges; geometries and QTAIM charges for all other species; worked examples of *γ* calculations; results for alternative charge models. See DOI: 10.1039/c5sc02076j


**DOI:** 10.1039/c5sc02076j

**Published:** 2015-08-14

**Authors:** Marcus C. Durrant

**Affiliations:** a Department of Applied Sciences , Northumbria University , Newcastle-upon-Tyne , NE1 8ST , UK . Email: marcus.durrant@northumbria.ac.uk

## Abstract

From the inception of Lewis' theory of chemical bonding, hypervalency has remained a point of difficulty that has not been fully resolved by the currently accepted qualitative definition of this term. Therefore, in this work, a quantitative measure of hypervalency has been developed. The only required input is the atomic charge map, which can be obtained from either quantum calculations or from experiment. Using this definition, it is found that well-known species such as O_3_, CH_2_N_2_ and ClO_4_^–^, are indeed hypervalent, whilst others such as XeF_4_, PCl_5_ and SO_4_^2–^, are not. Quantitative analysis of known species of general formulae XF_*n*_^*m*–^, XCl_*n*_^*m*–^, and XO_*n*_^*m*–^ shows that there are no fundamental differences in chemical bonding for hypervalent and non-hypervalent species. Nevertheless, hypervalency is associated with chemical instability, as well as a high degree of covalent rather than ionic bonding. The implications for accepted Lewis structure conventions are discussed.

## Abstract 2

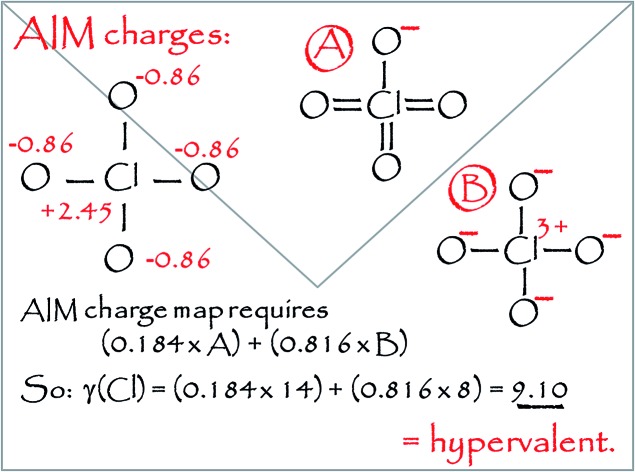
The concept of hypervalency has had a long but troubled history. Although several attempts have been made to dispense with the concept altogether, it remains in widespread use. By developing a simple but quantitative measure of hypervalency, the concept can be rehabilitated to provide valuable chemical insights in the context of Lewis models.

## Introduction

**Scheme 1:**
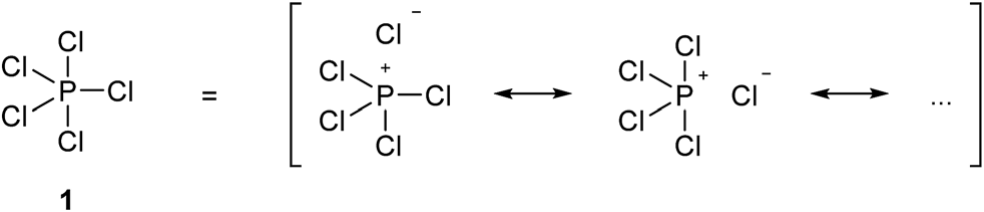
Conventional and resonance hybrid representations of PCl_5_.

In 1916, Gilbert Lewis laid one of the foundations of chemical bonding theory in his seminal paper ‘The Atom and the Molecule’.^1^ This work introduced two important concepts. Firstly, most chemical bonds are formed by electron pairs that are shared between adjacent atoms within a molecule in two-centre two-electron (2c-2e) covalent bonds. Secondly, in general, main group atoms within a molecule have a total of eight electrons in their valence shell, the so-called octet rule. Lewis also emphasized a third concept, perhaps less well remembered but equally important, that ‘the distinction between the most extreme polar and nonpolar types is only one of degree, and that a single molecule, or even part of a molecule, may pass from one extreme type to another, not by sudden and discontinuous change, but by imperceptible gradations’. In other words, all chemical bonds lie somewhere on a continuous spectrum between pure ionic and pure covalent. The further development of Lewis' theory and the emergence of the concept of hypervalency have been summarized by Jensen.^2^ In particular, it soon became clear that the two principles of the 2c-2e bond and the octet rule were sometimes in conflict. Over time, the position championed by Langmuir, namely that the octet rule should be observed when writing molecular formulae by the use of formal charges to define partially ionic bonds, came to be accepted, at least for the elements in period 2. For period 3 and beyond, however, this point of view was never entirely satisfactory, most notably because of the need to invoke purely ionic bonds for some compounds. For example, in the gas phase PCl_5_ is a discrete molecule that can be made to obey the octet rule by writing out a set of ionic resonance hybrids as shown in Scheme 1, but solid PBr_5_ actually exists as separate [PBr_4_]^+^ and Br^–^ ions. Therefore, in order to avoid confusion between a single ionized resonance form and a truly ionic species, structure **1** is accepted as the conventional representation of PCl_5_. Since in structure **1** the P atom has 10 valence electrons in five 2c-2e bonds, it is considered to have an ‘expanded octet’, leading to the concept of hypervalency. In 1969, Musher proposed the following definition of hypervalency; ‘we classify as “hypervalent” molecules and ions all those molecules and ions formed by elements in Groups V–VIII of the periodic table in any of their valences other than their lowest stable chemical valence of 3, 2, 1, and 0 respectively’.^3^ This is the currently accepted definition.

**Scheme 2:**
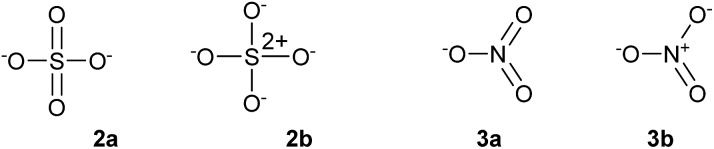
Alternative descriptions of the sulphate (**2**) and nitrate (**3**) ions.

**Scheme 3:**
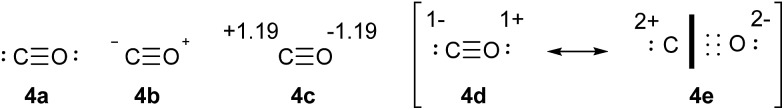
Lewis structure of CO (**4a**), associated formal charges (**4b**), QTAIM calculated charges (**4c**), and covalent (**4d**) *versus* ionic (**4e**) resonance forms.

The concepts of the electron pair, the octet rule and hypervalency have been forensically examined by Gillespie and co-workers.^4^ They pointed out that even though individual ionic resonance structures such as those shown in Scheme 1 have eight valence electrons, the total number of electrons involved in all five P–Cl bonds is nevertheless still 10 and so PCl_5_ breaks the octet rule as formulated by Lewis. They also suggested that the term ‘hypervalent’ has no practical use, since the chemical bonding in supposedly hypervalent molecules is no different to that found in non-hypervalent molecules, as revealed by analysis of the electron localization function (ELF) obtained from quantum calculations. Moreover the ELF analysis indicates that molecules such as SeMe_6_, in which the Se–C bonds are relatively non-polar, can have electron populations exceeding 8 at the central atom. According to Gillespie, it follows that species such as the nitrate and sulphate ions can be written in entirely analogous ways, as shown in Scheme 2, **2a** and **3a**. Although Gillespie's logic has never been refuted, it has, unfortunately, been ignored by the wider chemical community, and the formally charged species **3b** is almost universally insisted upon, in historical deference to the octet rule. Meanwhile, although Musher's definition of hypervalency may not be ideal, it is difficult to avoid such a term for known molecules such as the neutral NH_4_ radical^5^ and CLi_6_,^6^ which are clearly anomalous in terms of the Lewis model.

The advent of quantum mechanics has greatly extended our understanding of chemical bonding; in particular the 3c-4e bonding concept provides a rationale for the bonding in a wide range of molecules that can be described as hypervalent.^7^ Nevertheless, the marriage of quantum theory with the Lewis model has not always been a happy one. Most notoriously, the concept that elements beyond the second period can use d-orbital hybridization provided a convenient rationalization of how such elements can ‘expand their octet’. This explanation has been shown to be incorrect;^8^ instead, the large ionic contribution to the bonding in species such as PCl_5_ and SO_4_^2–^ means that the central atom's share of the bonding electrons sums to no more than eight, even though more than eight electrons may be required to construct all of the bonds. Gillespie has (disapprovingly) referred to this concept as the ‘modified octet rule’.^4^ For example, Schmøkel *et al.* have recently analysed the bonding in K_2_SO_4_ by both experimental and theoretical methods.^9^ They established that the S–O bonds are highly polarized, concluding that the octet-compliant structure **2b** in Scheme 2 is a better description of sulphate than the hypervalent structure **2a**. A very similar conclusion was previously reached by Reed and Schleyer, on the basis of quantum calculations.^8^ Nevertheless, this raises a new problem for classical Lewis octet compounds; for example, in PF_3_ the P–F bonds are highly polarized, such that PF_2_^+^F^–^ resonance forms, in which P has only six valence electrons, are predominant. Hence, the modified octet rule adds a new complication that detracts from the simplicity of the Lewis octet rule. Such studies also highlight the difficulties of reducing the detailed interpretation of sophisticated electronic structure calculations back down to the level of elementary concepts such as bond orders and electron pairs, in stark contrast to the simplicity of Lewis models which can be constructed on the back of the proverbial envelope.

In view of the utility of Lewis models and the desirability of a simple, general, and unified picture of chemical bonding, in this work a new method for electron counting is proposed. In the spirit of Lewis' original concepts, this method does not make direct use of any form of quantum calculations, but rather depends only on the atomic charges. These can be obtained from either experiment or theory, using Bader's Quantum Theory of Atoms in Molecules (QTAIM),^10^ with consistent results in either case. The new method leads naturally to a quantitative definition of hypervalency. It is shown that some molecules and ions are indeed hypervalent, that these include examples from period 2, and that hypervalency is generally associated with highly covalent bonding and chemical instability. It may be noted here that with the single exception of OF_4_, all of the molecules and ions mentioned throughout this paper have been characterized by at least one experimental study, in order to avoid any possibility of a misinterpretation based on a purely hypothetical species.

## Results and discussion

### Calculation of atomic charges

The initial goal of this study was to identify a suitable quantum method for the calculation of atomic charges. In recent years, the experimental determination of atomic charges from electron densities obtained by X-ray crystallography has become fairly routine. Such data can be compared directly with theoretical values obtained by QTAIM analysis of the output from quantum calculations.^10^ A search of the literature provided a test set of 17 molecules and salts for which QTAIM-compatible atomic charges have been reported, giving a total of 235 data points.^9^,^11^ These experimental charge values include data for nine individual elements, obtained in 12 different laboratories, ranging from +4.27 (S atom in K_2_SO_4_) to 1.45 [N atoms in (H_2_N)_2_CSO_2_]. Full details of the test set molecules are given in the ESI.†

This test set was used to evaluate eight different quantum methods, as detailed in Table 1. In each case, QTAIM charges were calculated post-SCF and compared graphically with the experimental data, using the *R*^2^ values for plots of obs. *versus* calc. charges, together with the RMS (obs. – calc.) errors to evaluate the various methods. For comparison, a few of the test compounds included values for more than one crystallographically independent molecule; a plot comparing these different experimental data gave *R*^2^ = 0.981, RMSE 0.099 (68 data points).

**Table 1:** Evaluation of quantum methods for QTAIM charge calculations

Method number	Procedure	*R* ^2^ value	RMS error
1	wB97XD/6-311+G(d,p), full geometry optimization	0.961	0.156
2	Method 1 for geometry optimization, followed by single point using wB97XD/6-311++G(3df,2pd)	0.958	0.166
3	Method 1 for geometry optimization, followed by single point using B3LYP/6-311++G(3df,2pd)	0.956	0.166
4	Method 1 for geometry optimization, followed by single point using MP4/6-31+G	0.955	0.168
5	wB97XD/DGDZVP, full geometry optimization	0.966	0.147
6	Method 1 for geometry optimization, followed by single point using MP4/DGDZVP	0.954	0.210
7	B3LYP/DGDZVP, full geometry optimization	0.966	0.145
8	MP2/DGDZVP, full geometry optimization	0.959	0.164

All eight methods in Table 1 gave good results, confirming that, as expected, QTAIM analysis is relatively insensitive to the choice of quantum method. The two most expensive methods using Møller–Plesset MP4 single point calculations gave relatively poor results, whilst for the DFT methods there was no improvement when using a large basis set over medium sized ones. DFT methods 5 and 7 using the wB97XD and B3LYP functionals respectively out-performed the MP2 and MP4 methods, and also gave very similar results to each other. Method 5 was selected for all subsequent calculations, since it gave marginally better performance than method 7 overall, and also the worst individual (obs. – calc.) value was better for method 5 than for method 7 (0.40 and 0.55 respectively).

### General principles; carbon monoxide

**Scheme 4:**
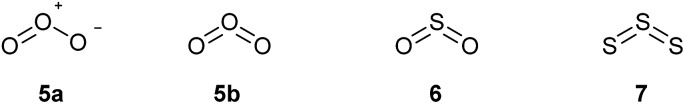
Alternative resonance forms of O_3_ and the conventional structures of SO_2_ and S_3_.

CO provides a very simple test case that can be used to establish some general principles concerning the relationships between charge and bonding. The conventional structure of CO is shown in Scheme 3, **4a**, and is consistent with the clear experimental and theoretical evidence that CO has a triple bond. Since the positions of all 10 valence electrons are defined, there is no need to specify formal Lewis charges; however, the implied charges are shown in **4b**. Meanwhile, the QTAIM charges, obtained by the standard procedure used throughout this work, are shown in **4c**. The contradiction between the formal Lewis charges and QTAIM calculated charges is immediately apparent. The resolution of this discrepancy is found in Lewis' concept of bond polarity. The six electrons of the triple bond are unequally shared between the C and O atoms, such that we may write extreme resonance forms as in **4d** and **4e**. It is important to emphasize that both of these structures represent a triple bond; **4d** is purely covalent, **4e** is purely ionic, but in both cases there are six bonding electrons and two lone pairs. The heavy black line in **4e** is intended to emphasize that all six bonding electrons are resident only on the oxygen atom. In both **4d** and **4e**, the O atom has eight valence electrons, whereas the C atom has eight in **4d** but only two in **4e**.

In order to calculate the overall electron count at the C atom, we may now define a parameter called the valence electron equivalent, *γ*, as ‘the formal shared electron count at a given atom, obtained by any combination of valid ionic and covalent resonance forms that reproduces the observed charge distribution’. Mathematically, if
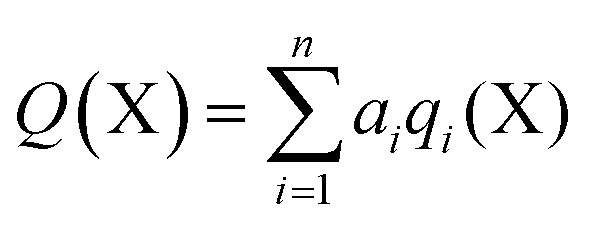
then
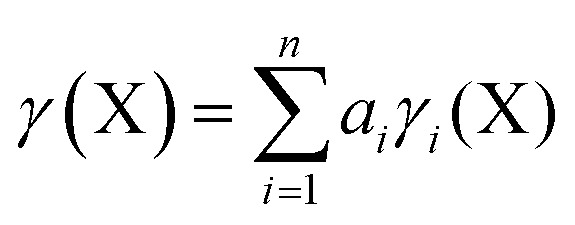
where Q(X) and *γ*(X) are the overall charge and valence electron equivalent of atom X, *q*_*i*_(X) and *γ*_*i*_(X) are the formal charge and electron count of each contributing resonance form, and *a*_*i*_ are the coefficients required to reproduce Q(X). It follows that for any given atom X, if *γ*(X) = 8, the atom obeys the original Lewis octet rule. If *γ*(X) < 8, the atom obeys the ‘modified octet rule’, but if *γ*(X) > 8, neither form of the octet rule is obeyed and the atom is hypervalent. In such a case, no combination of formally charged Lewis octet-compliant structures can reproduce the observed charge distribution and it is necessary to include a contribution from an ‘expanded octet’ structure. Taking CO as a worked example, the triple bond is quite heavily polarized toward the more electronegative oxygen atom, and a mixture of 27% of **4d** plus 73% of **4e** is required to reproduce the observed charges, such thatCharge on C = (0.27 × –1) + (0.73 × +2) = +1.19

Then, using the same proportions,*γ*(C) = (0.27 × 8) + (0.73 × 2) = 3.62

Hence, the C atom obeys the Lewis octet rule if the charge distribution is ignored, but obeys the modified octet rule if the charges are taken into account. However, the more electronegative O atom has eight electrons in both **4d** and **4e**. Thus, the Lewis octet rule is strictly obeyed for the more electronegative atom. This is a general principle that must be observed when choosing valid resonance forms for the calculation of *γ* values.

Using this general methodology, it is possible to calculate *γ* for any atom in any closed shell molecule or ion, provided only that the charge distribution is available from experiment or theory. As long as the standard rules of covalent bonding are applied, there is no need to carry out any detailed quantum analysis of the bond orders by ELF or QTAIM calculations. The only limitation in choosing valid resonance forms is that for the most electronegative atom(s) in a given structure, *γ* must be ≥8 in all component resonance forms, and exactly 8 if the atom occupies a terminal position. When bonded atoms carry opposite charges, these can be eliminated by increasing or decreasing the bond order as required, even if this results in hypervalent centres. It is often not necessary to consider the complete charge distribution, but only the charges of the atom of interest and the summed charges of the fragments to which it is bonded. A crucial point is that although various different combinations of resonance forms may be used to reproduce the observed charges, each of these combinations yields the same value of *γ*; hence, *γ* is uniquely determined from the charge map. The ESI† includes a selection of worked examples of *γ* calculations.

As noted above, the standard Lewis description of CO leads to formal charges that contradict the true charge distribution. This is by no means an isolated case; another simple example is the ammonium ion. The formal Lewis charge for NH_4_^+^ must be placed on the N atom, whereas QTAIM calculations show a charge of –0.89 on N and +0.47 on each H atom. The well-established chemistry of the NH_4_^+^ ion is in good qualitative agreement with this picture. As with CO, the true charges contradict the formal Lewis charges, but are in good agreement with the relative electronegativities of the component atoms; as indeed is generally the case. To summarize; formal Lewis charges are used for electron accounting purposes, but have no more than a purely coincidental relationship with the true atomic charges, which originate instead from the relative electronegativities of the component atoms.

### Relationship between the valence electron equivalent and bond energies

**Fig. 1:**
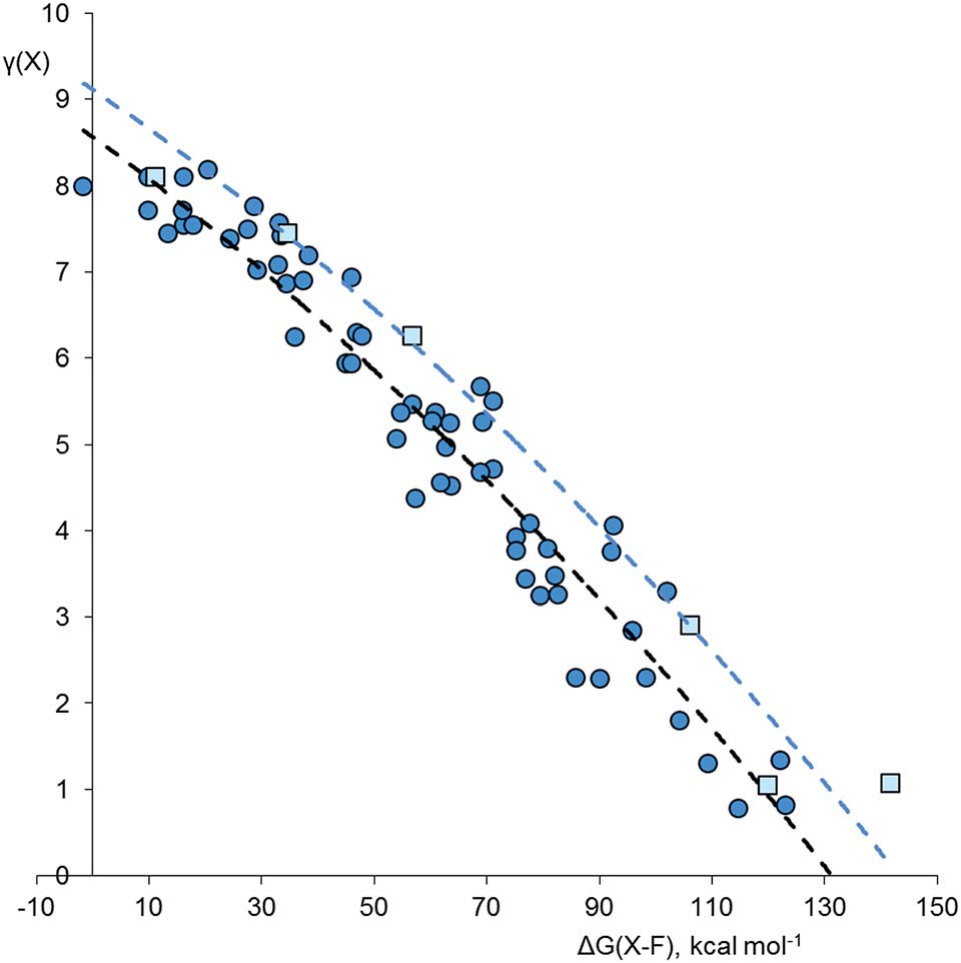
Plot of *γ*(X) *versus* Δ*G*(X–F) for fluorides XF_*n*_^*m*–^. Points for second row and heavier elements are represented by light blue squares and dark blue circles respectively. The dashed curves are an empirical fit to the two data sets, offset by 11.9 kcal mol^–1^ to the right for the second row elements. Correction factors; –1.4 and –2.8 kcal mol^–1^ for explicit and implicit Rb^+^ respectively.

**Fig. 2:**
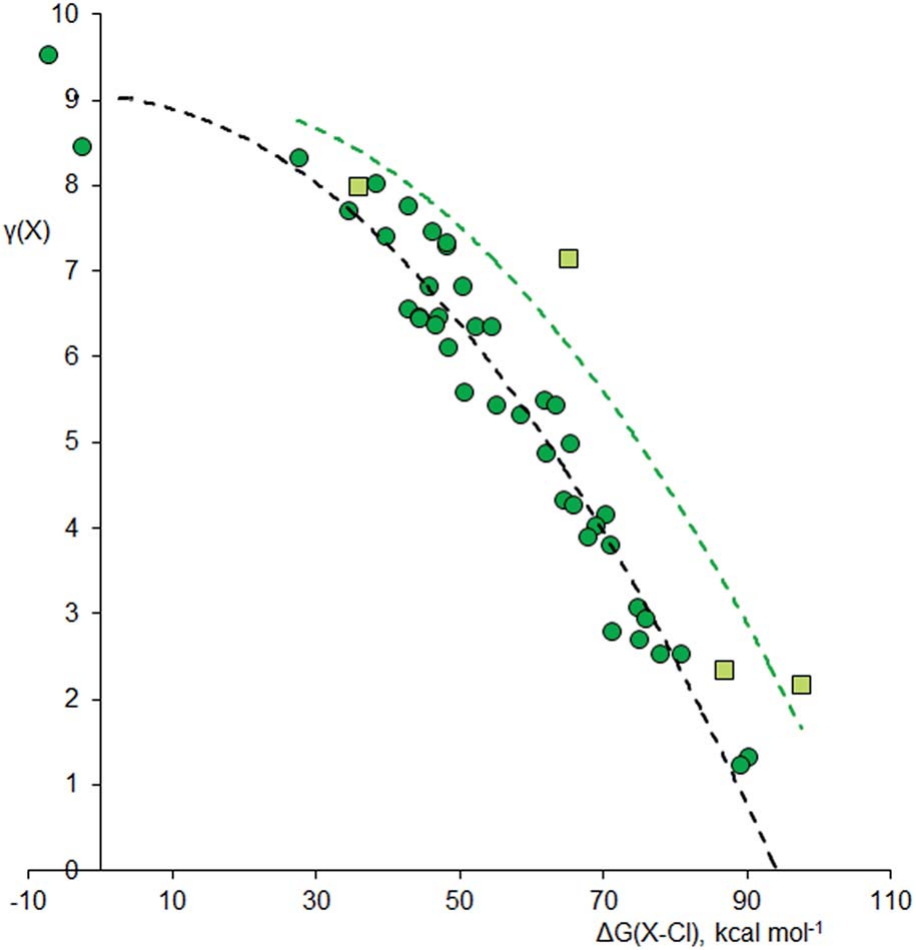
Plot of *γ*(X) *versus* Δ*G*(XCl) for chlorides XCl_*n*_^*m*–^. Points for second row and heavier elements are represented by light green squares and dark green circles respectively. The dashed curves are an empirical fit to the two data sets, offset by 12.7 kcal mol^–1^ to the right for the second row elements. Correction factors; –20.2 and +25.4 kcal mol^–1^ for explicit and implicit Rb^+^ respectively.

**Fig. 3:**
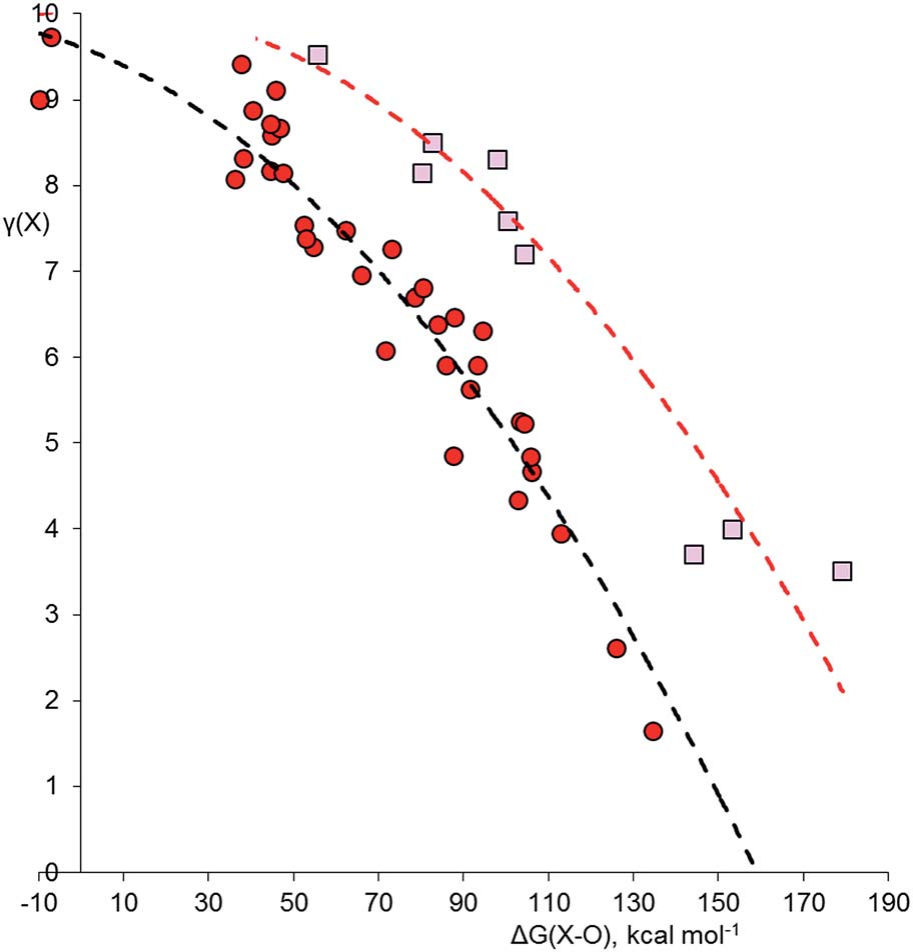
Plot of *γ*(X) *versus* Δ*G*(X–O) for oxides XO_*n*_^*m*–^. Points for second row and heavier elements are represented by pink squares and red circles respectively. The dashed curves are an empirical fit to the two data sets, offset by 43.5 kcal mol^–1^ to the right for the second row elements. Correction factors; –9.6 and +56.2 kcal mol^–1^ for explicit and implicit Rb^+^ respectively.

Using the method described above, the values of *γ* for the central atom in a series of 66 fluorides XF_*n*_^*m*–^, 46 chlorides XCl_*n*_^*m*–^, and 45 oxides XO_*n*_^*m*–^ (*n* = 2–8, *m* = 0–6) were calculated. References for the more exotic species in this and subsequent sections are given in the ESI.† The values of *γ*(X) so obtained were then plotted against the bond free energies Δ*G*, also obtained by quantum method 5. The results are shown in Fig. 1–3 respectively. Using the fluorides as an example, for the neutral molecules, the values of Δ*G*(X–F) were simply obtained by dividing the value of Δ*G* for the general atomization reaction, eqn (1), by the value of *n*;

1 [XF_*n*_] → [X] + *n*[F]

where each species in square brackets denotes an individual calculation. For the anions, a somewhat more complicated procedure was required. Rubidium was chosen as a counterion, since this gives a high degree of ionic bonding between the anion and cation. However, the inclusion of an explicit Rb^+^ centre in weakly bound compounds such as RbF_3_ led to heavily distorted geometries, since the Rb–F bond is stronger than the F–F bonds in the F_3_^–^ anion. The best solution was found to be the use of one implicit Rb^+^, together with the required number of explicit Rb centres. For example, F_3_^–^ was modelled as such, and the F–F bond energy was calculated using eqn (2);

2 [Rb^+^] + [F_3_^–^] → [Rb] + 3[F]

The extension of this approach to multiply charged species is illustrated in eqn (3), using phosphate as an example;

3 [Rb^+^] + [Rb_2_PO_4_^–^] → 3[Rb] + [P] + 4[O]

To allow for the effects of the Rb, correction factors were applied for the numbers of explicit and implicit Rb^+^ cations; the values of these parameters are given in the figure captions. This approach proved to give satisfactory results for all of the ions considered.

Considering the plot for fluorides in Fig. 1, there is a clear correlation between *γ*(X) and Δ*G*(X–F). Stronger bonds are highly polarized and have smaller *γ* values, as found for example in SiF_4_ [Δ*G* = 122 kcal mol^–1^, *γ*(Si) = 1.34], whilst weakly bonded molecules such as XeF_6_ have more covalent bonding and higher values of *γ* [Δ*G* = 10 kcal mol^–1^, *γ*(Xe) = 7.72]. Compounds of second row elements, such as F_2_O, tend to have higher *γ* values for a given Δ*G* than those of heavier elements. The resulting two data sets have been empirically fitted to a common parabolic curve, displaced by 11.9 kcal mol^–1^ for the second row elements.

Closer inspection of Fig. 1 shows that there are four species for which *γ*(X) > 8. These are the ClF_6_^–^ ion [*γ*(Cl) = 8.19], the F_3_^–^ ion [*γ*(F) = 8.11], XeF_3_ [*γ*(Xe) = 8.11], and ClF_5_ [*γ*(Cl) = 8.10]. These values are only slightly greater than 8, and could perhaps be accounted for by the margin of error of the calculations. Nevertheless, taking the data at face value, these four species are all hypervalent, both by Musher's qualitative definition, and by the present quantitative definition.

Since fluorine is the most electronegative element, fluorides tend to have particularly ionic bonding, so it is difficult for the central atom to retain a high electron density. Chlorine is less electronegative, and Fig. 2 shows the analogous plot for chlorides, XCl_*n*_^*m*–^. This plot is very similar to that for the fluorides; again, the second row elements have higher *γ* values for a given Δ*G*(X–Cl), but the curvature is more pronounced. There are three species that are clearly hypervalent, namely XeCl_4_ [*γ*(Xe) = 9.53], XeCl_2_ [*γ*(Xe) = 8.47], and SCl_4_ [*γ*(S) = 8.33]. For the Cl_3_^–^ ion, *γ*(Cl) = 8.04; hence this species is not considered to be hypervalent, at least using data from method 5.

Fig. 3 shows the plot for the oxides, XO_*n*_^*m*–^. Although O is more electronegative than Cl, many of the oxides require structures with double bonds, which might lead to higher *γ* values. The same trends as observed for the other two plots are again apparent. However, hypervalency is more common, with no fewer than 16 hypervalent molecules and ions (Table 2). As with the other plots, there is no discontinuity for species with *γ* > 8, indicating that there are no fundamental differences in the bonding between hypervalent and non-hypervalent species. Fig. 3 includes data points for seven neutral and anionic radicals. The odd electron is well known to be delocalized in most species of this type, and the best Lewis scheme for their bonding has been the subject of some debate. For the four XO_2_ radicals (X = N, P, Cl or Br), simply placing the odd electron on the central heteroatom gave an excellent fit to the rest of the data set; this also ensures that in all contributing resonance forms, the more electronegative O atoms always have *γ* = 8, as specified above. The same holds true for the PCl_4_, XeF_3_ and SF_3_ radicals in the other data sets. For the NO_3_ radical and the BrO_5_^2–^ and IO_5_^2–^ radical anions, there is no reasonable Lewis structure that does not have the radical on an O atom, and indeed this was confirmed by Mulliken spin state analysis; hence, these radical species require an exception to the general principle that the most electronegative atoms must have 8 electrons.

**Table 2:** Hypervalent oxides XO_*n*_^*m*–^

Species	*γ*(X)	Species	*γ*(X)
XeO_4_	9.72	ClO_2_^–^	8.58
O_3_	9.52	NO_4_^3–^	8.50
XeO_6_^4–^	9.41	ClO_2_	8.31
ClO_4_^–^	9.11	NO_3_^–^	8.30
XeO_3_	9.00	BrO_2_^–^	8.17
BrO_5_^–^	8.88	NO_3_	8.14
BrO_4_^–^	8.71	BrO_3_^–^	8.14
ClO_3_^–^	8.67	BrO_2_	8.07

Some of these oxides merit further discussion. Thus, in agreement with Schmøkel *et al.*,^9^ sulphate is not hypervalent [*γ*(S) = 4.34]; but perchlorate and perbromate are both quite markedly hypervalent [*γ*(Cl) = 9.11, *γ*(Br) = 8.71]. This is consistent with the relative electronegativities of the elements; the Pauling *Χ* values are 3.16 and 2.96 for Cl and Br respectively, compared to 2.58 for S. Table 2 also contains some second row species, namely ozone, orthonitrate, nitrate and the neutral NO_3_ radical. The conventional structure of nitrate is shown as **3b** in Scheme 2. However, the calculated charge on N is +0.85, less than the value of +1.0 required by **3b**. The observed charge is reproduced by a combination of (0.15 × **3a**) + (0.85 × **3b**), hence*γ*(N) = (0.15 × 10) + (0.85 × 8) = 8.30

**Scheme 5:**
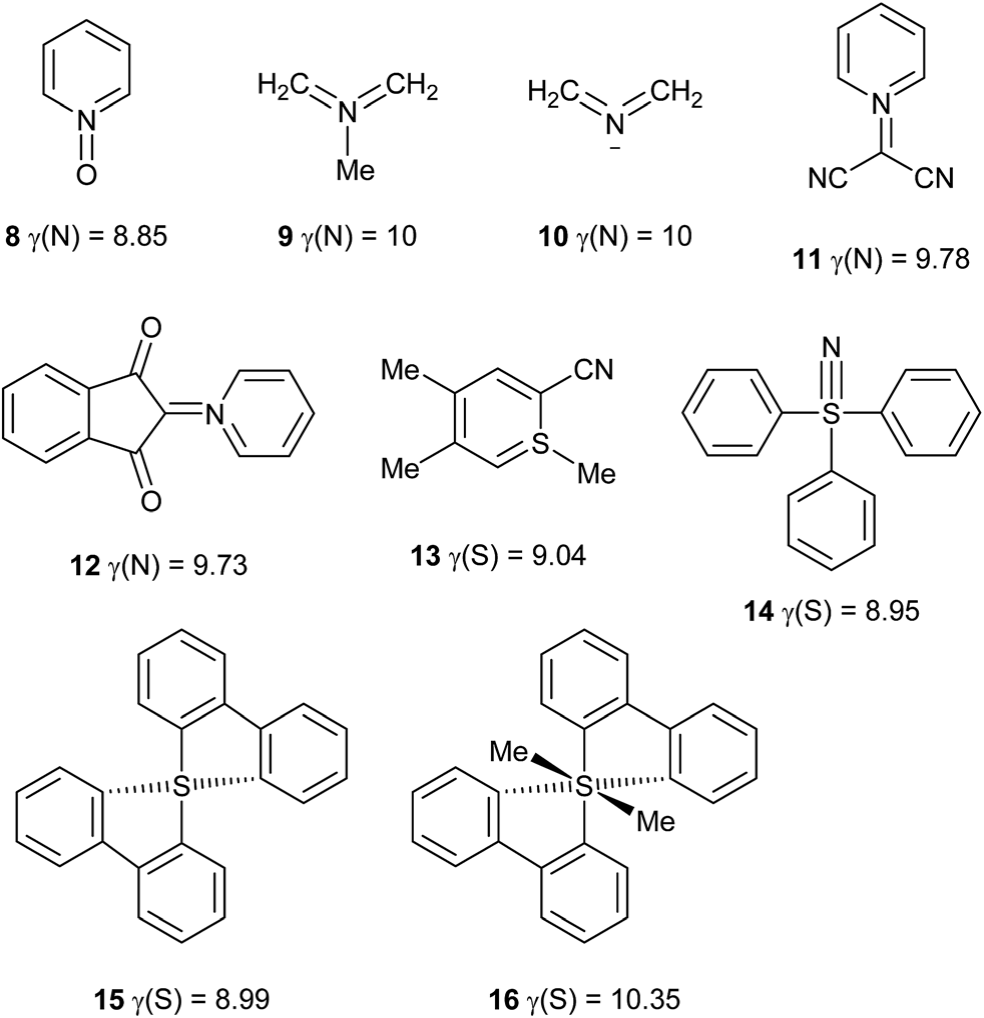
Hypervalent molecules and ions.

Similarly, the conventional formula for ozone, given in Scheme 4, **5a**, is in poor agreement with the observed charge distribution of +0.24 and –0.12 on the central and terminal O atoms respectively. The combination of (0.24 × **5a**) and (0.76 × **5b**) gives the correct charges and results in *γ*(O) = 9.52 for the central O atom. In this case, there is no electronegativity difference between the atoms, so the bonding is particularly covalent, leading to a high value of *γ* and relatively weak bonding. Bonding in the isoelectronic SO_2_ is both stronger and much more ionic11*c* [*γ*(S) = 5.25] and this species is not hypervalent, in spite of the fact that ozone is conventionally written as in **5a** and SO_2_ as in **6**; a convention which is an exact reversal of the true covalent *versus* ionic bonding trends for the two molecules. Moreover the charge distribution in S_3_ (Scheme 4, **7**) is very similar to that in ozone (+0.20 and –0.10 on the central and terminal S atoms respectively), giving *γ*(S) = 9.60; here again, there is no logical justification for distinguishing O_3_ from S_3_ by the use of structures **5a** and **7** respectively.

### Other hypervalent species

Using the definition of a hypervalent atom as one for which *γ* > 8, it is now possible to define conditions for which *γ* is maximized. First, multiple bonds, when required to satisfy the valences of the more electronegative atoms, tend to increase *γ*. Second, the electronegativity of the central atom should be similar to those of its neighbours. Based on these principles, in addition to the 24 hypervalent species discussed above, a further set of 36 hypervalent molecules and ions has been identified, as shown in Table 3 and Scheme 5. Note that where *γ*(X) is given as an integer, atom X has that value of *γ* in each of the component resonance structures required to reproduce the charges. Such species can therefore be considered as attaining maximum hypervalency. This is invariably the case when X is the most electronegative atom in the molecule or ion. It should be noted that most of these compounds give qualitative support for the association of hypervalency with instability, and indeed several of them are explosive.

**Table 3:** Hypervalent molecules and ions

Species	*γ*(X)	Species	*γ*(X)
CLi_6_	*γ*(C) = 10	FLi_2_	*γ*(F) = 9
HN_3_, N_3_^–^	*γ*(N) = 10	PPS	*γ*(P) = 8.94
CH_2_NN	*γ*(N) = 10	HArF	*γ*(Ar) = 8.63
CH_2_NCH	*γ*(N) = 10	HKrF	*γ*(Kr) = 8.58
NNS, PNS	*γ*(N) = 10	Me_3_NO	*γ*(N) = 8.56
NS_2_^–^	*γ*(N) = 10	ClNO_2_	*γ*(N) = 8.55
OLi_4_	*γ*(O) = 10	HXeF	*γ*(Xe) = 8.39
XeF_2_O_3_	*γ*(Xe) = 9.32	CF_3_NO_2_	*γ*(N) = 8.35
NNO	*γ*(N) = 9.28	Ph_3_I	*γ*(I) = 8.30
PNO	*γ*(N) = 9.20	MeONO_2_	*γ*(N) = 8.26
HCNO^*a*^	*γ*(N) = 9.14	MeNO_2_	*γ*(N) = 8.13
NH_4_ radical	*γ*(N) = 9	PhNO_2_	*γ*(N) = 8.13
N_5_^+^	*γ*(N) = 9	Ph_4_Se	*γ*(Se) = 8.10

^*a*^ *γ*(N) = 8.70 for CNO^–^ ion.

**Scheme 6:**
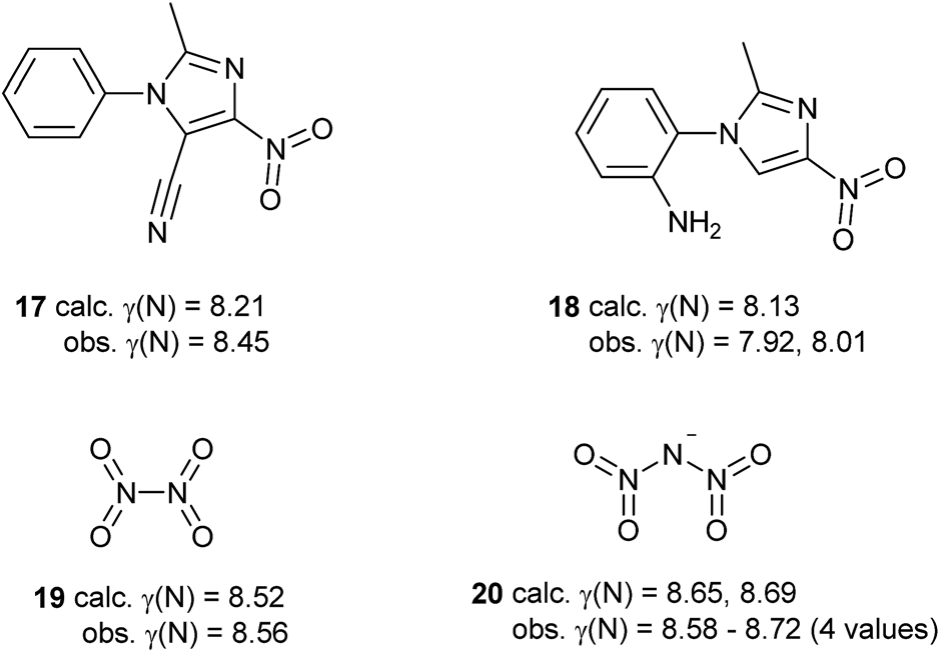
Comparison of *γ*(N) values for nitro groups obtained by experimental and theoretical methods.

Some of the species in Table 3 and Scheme 5 merit further comment. Like nitrate, simple aromatic and aliphatic nitro compounds are found to be moderately hypervalent. This is interesting since the nitro group is a textbook example of a moiety that is made to obey the octet rule by the use of arbitrary charges. These results have been cross-checked using the experimental data for the four compounds from the test set that contain nitro groups, Scheme 6. There is generally good agreement between *γ*(N) values obtained from experimental and theoretical charges. Three of these species are hypervalent by both theory and experiment; the exception is compound **18**, which has experimentally determined *γ*(N) values which are very close to 8 for the two independent molecules in the unit cell. This arises from the unusually strong polarization of the nitro group, which carries a total charge of –0.75 and –0.76 in the two crystallographically independent molecules, compared to *e.g.* –0.59 for compound **17**. Hence, the contribution from the R^+^·NO_2_^–^ [*γ*(N) = 8] resonance form is particularly large for **18**. This possibility does not arise for the N–NO_2_ species **19** and **20**, which consequently have higher values of *γ*(N). A similar explanation can be applied to the greater hypervalency of CF_3_NO_2_ compared to MeNO_2_ (Table 3).

Another interesting comparison can be made between the anion **10** in Scheme 5 and the isoelectronic neutral molecule CH_2_OCH_2_. Both have singlet ground states and similar charge distributions, but CH_2_OCH_2_ is known to have a diradical character.^12^ This serves to reduce the C–O bond orders from 2 to 1, avoiding the need for a hypervalent O atom. This suggests an important principle that the most electronegative atoms are able to retain lone pairs that less electronegative atoms can be made to use for bonding. For this reason, hypervalent compounds of O and F are very rare. A similar comparison can be made between the NH_4_ radical and the putative H_3_O radical, in that ND_4_ has been found to have a lifetime of >20 μs by ion-beam spectroscopy,^5^ whereas the lifetime of D_3_O is <1 ns.^13^

### Non-hypervalent species

**Scheme 7:**
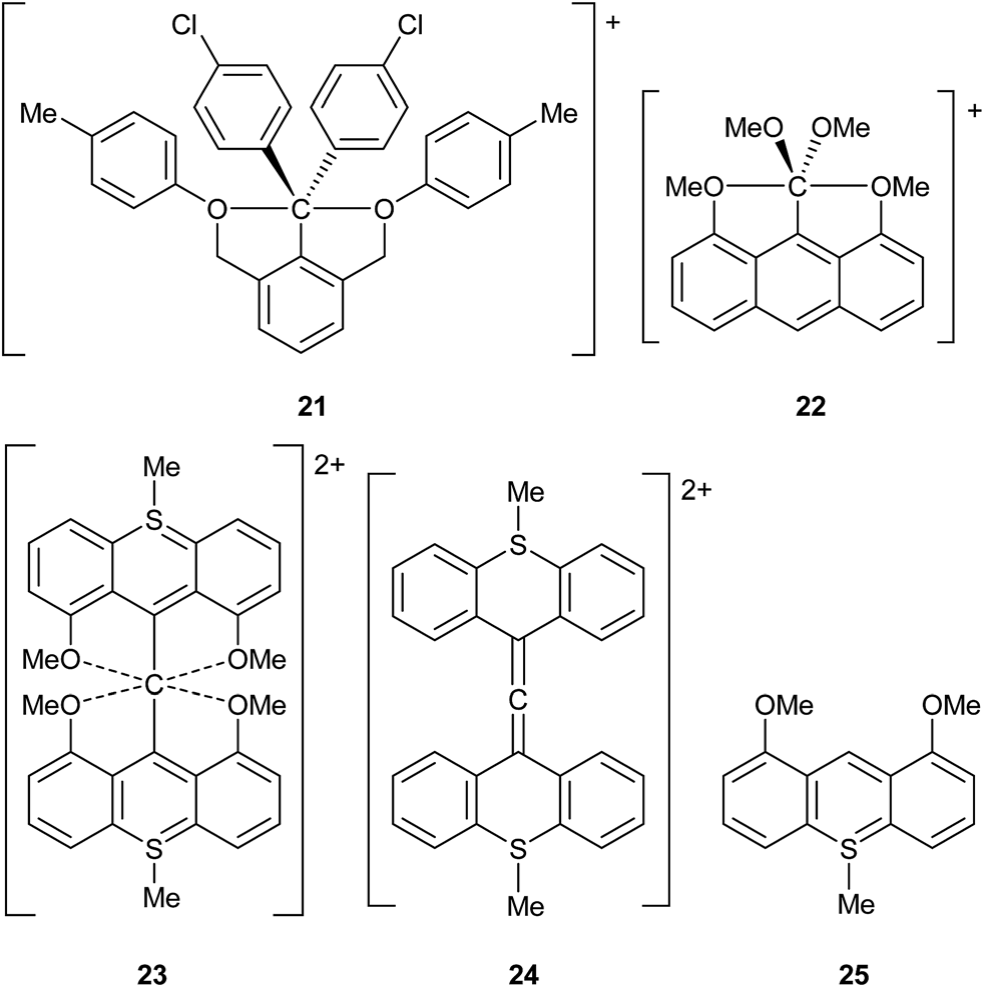
Hypercoordinate carbon compounds and derived fragments.

The use of *γ* values also sometimes rules out hypervalency in situations where it might have been anticipated. For example, it might be thought that C achieves a hypervalent state during the course of S_N_2 reactions.^14^ To test this idea, the saddle point structures Cl–CH_3_–Cl and Br–CH_3_–Br have been analysed by the usual procedure, giving *γ*(C) values of 7.15 and 7.25 respectively. These can be compared to *γ*(C) values of 7.46 and 7.70 for CH_3_Cl and CH_3_Br respectively; hence, *γ*(C) actually *decreases* during S_N_2 reactions, due a switch from more covalent to more ionic C–X bonds in the transition state. Akiba *et al.* have prepared several fascinating hypercoordinate and potentially hypervalent carbon compounds, as shown in Scheme 7, **21–23**.^14^ Although **21** and **22** both break the Lewis octet rule, their C–O bonds are quite polarized, such that they have *γ*(C) values of 6.61 and 5.01 respectively; hence neither of these cations is hypervalent by this measure. Cation **23** presents a particular problem as it can be formulated either as an allene, or as having a six-coordinate C atom. The former would not be hypervalent, whilst the latter would have *γ*(C) = 8.83. In order to better choose between these two alternatives, calculations have also been done on the two fragments **24** and **25**. The calculated charge on the central C of allene **24** is –0.53, compared to –0.42 for **23**; moreover the charges on the O atoms in **25** and **23** are nearly identical at –1.11 and –1.12 respectively. Hence, there is no evidence for the charge redistribution from O to C that would be required for the hypervalent form of **23**. Moreover, the calculated C=C bond lengths in **23** and **24** are identical at 1.317 Å (the experimental values14*c* for **23** are 1.310 and 1.319 Å). It is also worth pointing out that formula **23** has three hypervalent centres, since the two S atoms have *γ*(S) = 8.83; all three hypervalent centres are obviated by the allene formulation. Hence the latter seems to be more appropriate, notwithstanding a weak bonding interaction between the central C and the O atoms as revealed by QTAIM analysis.

The SiH_6_^2–^ anion has been obtained as its K^+^ and Rb^+^ salts from high pressure synthesis.^15^ Although this ion could be considered as hypervalent, the present analytical method gave *γ*(Si) = 2.78 for the naked anion. This is consistent with the fact that H is more electronegative than Si (*Χ* = 2.20 and 1.90 respectively), giving the H atoms hydride character in this species. For comparison, the published^15^ QTAIM analysis of K_2_SiH_6_ leads to *γ*(Si) = 3.00. Similarly, the [Ph_3_SiH_2_]^–^ anion gives *γ*(Si) = 2.57, consistent with experimental and computational characterization which revealed a hydridic character.^16^ In general, the preparation of hypervalent compounds of elements less electronegative than H is likely to be problematic. For example, hypervalent examples of phosphorus (*Χ* = 2.19) seem to be very rare, the only example found in the present work being PPS (Table 3); the PS_4_^3–^ anion has *γ*(P) = 7.94.

### Alternative charge models

Over the years, many alternative methods for the calculation of atomic charges within a molecule have been devised, often providing markedly different results. Since the valence electron equivalent can be calculated from any self-consistent charge map, it is interesting to investigate the effects of different charge models on the value of *γ*. Table 4 compares the results of *γ* calculations on a variety of species, using quantum method 5 plus the QTAIM, Natural Bond Orbital (NBO), Hirshfeld and Mulliken charge models. In addition, the final row of the table includes RMS fit data for a subset of 10 of the neutral oxides XO_n_ used in the construction of Fig. 3; these values were obtained by optimization of the parabolic function used to correlate *γ*(X) with Δ*G*(X–O).

**Table 4:** Calculation of *γ* using alternative charge models

Species	*γ*(QTAIM)	*γ*(NBO)	*γ*(Hirshfeld)	*γ*(Mulliken)
**16**	10.35	9.56	11.33	10.44
CH_2_NN	10	9.91	9.78	9.56
XeO_4_	9.72	8.98	14.42	12.49
O_3_	9.52	9.35	9.56	9.43
SCl_4_	8.33	8.46	8.98	8.81
MeNO_2_	8.13	8.41	9.13	8.74
Cl_3_^–^	8.04	8.10	8.22	8.08
KrF_2_	7.99	7.94	9.12	8.34
ICl_4_^–^	7.72	7.79	9.47	8.58
SO_2_	5.25	6.80	9.05	8.12
SO_3_	4.85	7.13	10.69	9.17
SO_4_^2–^	4.34	6.78	11.53	9.39
PF_6_^–^	2.30	4.40	8.96	6.90
RMS	0.426	0.494	0.918	0.990

As revealed by Table 4, there is a reasonable straight line correlation between *γ* values obtained by the QTAIM and NBO charge models (*R*^2^ = 0.899 for a set of 25 data points), although the range of *γ* values is narrower for NBO. These two methods both give good correlations between *γ*(X) and Δ*G*(X–O) and are also in excellent qualitative agreement over which species in Table 4 are hypervalent. In contrast, the Hirshfeld and Mulliken schemes return markedly different *γ* values and also give much poorer correlations between *γ*(X) and Δ*G*(X–O). Furthermore, the latter two models both predict that SO_2_ and SO_4_^2–^ are hypervalent, in clear disagreement with the experimental and theoretical consensus that these species have highly polarized, non-hypervalent bonding.^8^,^9^,11c

Bader and Matta have provided a robust defence of the choice of QTAIM for the calculation of atomic charges.^17^ They pointed out that ‘charge, as defined within QTAIM, is the measurable expectation value of a Dirac observable and is now routinely determined in accurate X-ray diffraction experiments on crystals’. Interestingly, they also noted (and refuted) the widespread notion that QTAIM charges are exaggerated in magnitude. This bears directly on the results in Table 4; since the Hirshfeld and Mulliken methods generally give smaller absolute charges than QTAIM, they overestimate the covalent contribution, leading to improbably high values of *γ*. Strongly ionic species such as RbF provide a good indicator of whether a given charge method will be valid for *γ* calculations; QTAIM and NBO both predict a charge on the Rb^+^ ion of +0.94, whereas the Hirshfeld and Mulliken methods predict unreasonably low charges of +0.67 and +0.78 respectively.

To summarize; although many charge models are available, QTAIM charges are derived from a theoretically rigorous procedure originating directly from the underlying physics. Another key advantage in the present context is that QTAIM charges can also be obtained directly from experimental data without any use of quantum calculations, which is not the case for NBO. Moreover, QTAIM gives the best correlation between *γ*(X) and Δ*G*(X–O); and together with NBO, correctly predicts that SO_2_ and SO_4_^2–^ are not hypervalent. Hence, QTAIM is recommended for the current application, although some other charge models such as NBO would lead to very similar conclusions.

### Reappraisal of the octet rule

The original Lewis octet rule is obeyed by all main group elements in their lowest common valencies. Since the group 14 elements have four valence electrons, their compounds naturally tend to have eight bonding electrons and they almost always obey the rule, with very few exceptions such as CLi_6_ and SiH_6_^2–^. O and F also tend to obey the rule, since as discussed above, these very electronegative elements are evidently reluctant to give up lone pairs for the formation of additional bonds. Homonuclear species such as O_3_ and F_3_^–^ provide rare exceptions. The presently unknown OF_4_ would provide another [*γ*(O) = 8.71] and should be marginally stable [calculated Δ*G*(O–F) = +4.4 kcal mol^–1^], although it would doubtlessly be explosive. Of the second row elements, only N, with five valence electrons but lower electronegativity, has the right combination of properties for hypervalency to be a relatively common feature of its chemistry. Even then, the small size of the N atom means that with the exception of the NH_4_ radical, all of the hypervalent N species identified in this work have multiple bonds. This tends to obscure the presence of hypervalency, by the invocation of formal charges to reduce the apparent bond order in conformity with the Lewis octet rule. Nevertheless, as Gillespie has observed, the fact that one can always write a structure that is consistent with the octet rule does not provide any evidence for the legitimacy of that rule.4*a* It is interesting to note that as long ago as 1997, valence bond theory calculations had established a hypervalent formulation of diazomethane;^18^ however this result has again been largely ignored by the wider chemical community.

Beyond the second row, atoms are larger, whilst their lower electronegativities render their lone pairs more available for conversion into bonding electrons. Hence, violations of the original Lewis octet rule are commonplace for those elements with more than four valence electrons, leading to the concepts of the ‘expanded octet’ and the ‘modified octet rule’. The ‘expanded octet’ concept is still in widespread use to describe the observed chemistry of these elements, but has lacked any proper theoretical basis since the possibility of extensive d-orbital participation was discredited a quarter century ago.^8^ Meanwhile, the many exceptions to the ‘modified octet rule’ described in this paper indicate that this concept is misleading and must be discarded. In so doing, the original Lewis octet rule can be reinstated, as a useful rule of thumb for the chemistry of the elements in their lowest common valencies, but certainly not as a fundamental and inviolable chemical principle. Exactly the same status is of course already accepted for Lewis' 2c-2e rule and also the 18-electron rule.

**Scheme 8:**
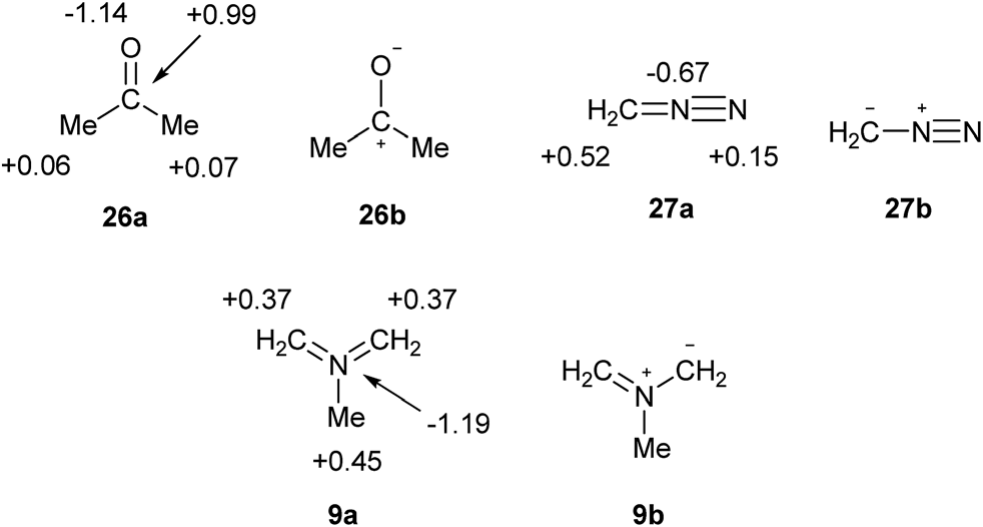
Calculated charge maps (a) and formally charged resonance forms (b) for acetone, diazomethane and azomethine ylide.

What are the implications for the writing of chemical formulae? At the present time, the perceived need to adhere to the octet rule results in formal charges that have no fundamental meaning, and often poorly reproduce or even contradict the actual charge maps; such a model can scarcely be considered to be beyond improvement. The problem is avoided for elements beyond the second row by allowing multiple bonds at the expense of octet rule compliance. There is now a consensus that such bonds are often highly polarized, as in the cases of sulphate and phosphate. However, it is also undeniably the case that many familiar octet-compliant structures also incorporate highly polarized bonds. For example, consider the charge map for acetone, **26a** in Scheme 8. The single most accurate representation of this structure is clearly **26b**, and indeed this form is invoked in countless reaction mechanisms. Nevertheless, **26a** is the standard formula. Since it is implicitly accepted that bonds such as C=O and S=O can be quite polar in nature, there is no logical reason to insist on the specification of precise but arbitrary ionic contributions only in those cases of second row elements where the octet rule would otherwise be violated, such as nitro compounds. Moreover, the observed charge maps for such species can often only be reproduced by including a contribution from the fully covalent, hypervalent formulae. Particularly striking examples of the failure of the current convention to predict atomic charges are provided by **27** and **9** [which both have *γ*(N) = 10] in Scheme 8. As usual, the observed charges are consistent with the relative electronegativities, and the hypervalent formulae are the most logical option.

## Conclusions

Use of the valence electron equivalent *γ* provides a simple but general and robust quantitative method for assessing hypervalency in molecules and ions, based only a map of the atomic charges. Bond orders are assigned by conventional chemical principles, using electronegativities to prioritize different atoms, without any recourse to detailed analysis of quantum calculations, with all its attendant complexities. Quantum calculations will, of course, continue to provide our deepest level of understanding of all types of chemical bonding, including hypervalency. Nevertheless, the Lewis approach provides a simple, robust and, above all, useful conceptual framework that has always been essentially independent of quantum mechanics. Since QTAIM charges can be obtained from experiment as well as from theory, the present work preserves that independence whilst refining the application of Lewis' concepts to hypervalent molecules. It is important to note that although more accurate experimental or theoretical charge data might lead to some revision of *γ* values for individual species in the future, the methodology itself is robust and generally applicable.

Many species that would be considered hypervalent by Musher's definition, such as PCl_5_, SO_4_^2–^, XeF_6_*etc.* can be described as hypercoordinate but not hypervalent according to their *γ* values. Rather, such species show a high degree of ionic bonding that relieves electron density at the central atom, such that *γ* < 8. Nevertheless, it is certainly possible for *γ* to exceed 8; the largest *γ* value identified in this work is 10.35 for molecule **16**, whose hypervalency has already attracted theoretical interest.^19^

Plots of *γ versus* Δ*G* show that the chemical bonding in hypervalent species is generally highly covalent and relatively weak, but not fundamentally different to that in non-hypervalent species. Roughly speaking, the elements located on the diagonal from N to Xe in the p-block each have a suitable combination of more than four valence electrons and midrange electronegativities, rendering them particularly suitable for the manifestation of hypervalency.

Finally, the writing of octet-compliant, formally charged structures for second row elements is currently required by tradition, but not for any fundamental chemical reason, and indeed produces incorrect charge descriptions for many molecules and ions. For heavier elements, expanded octet structures are the norm, with the implicit understanding that both single and multiple bonds will often have a highly polar character. There are no fundamental differences in chemical bonding between the second row and heavier elements, although the former are smaller and tend to be more covalent. Therefore, the formulation of multiply bonded, formally hypervalent second row structures such as **3a** for nitrate or **27a** for diazomethane should no longer be considered as incorrect by the chemical community.

## Computations

All quantum calculations were carried out with Gaussian09 software.^20^ In each case, full geometry optimization was followed by a frequency calculation to check for the absence of imaginary frequencies and also to obtain thermochemical values, which were used as obtained for Δ*G* calculations. QTAIM analyses were done on the formatted Gaussian checkpoint files using AIMAll software.^21^

## Supplementary Material

Supplementary information
