# Supplementary material for: A quantitative definition of hypervalency
Source: Chem Sci. 2015 Aug 14;6(11):6614–23. doi: 10.1039/c5sc02076j (PMC6054109; doi:10.1039/c5sc02076j)
Supplement: Supplementary file 1 [file SC-006-C5SC02076J-s001.pdf]

Electronic Supplementary Material (ESI) for Chemical Science.
This journal is © The Royal Society of Chemistry 2015

SUPPORTING INFORMATION

A Quantitative Definition of Hypervalency

M. C. Durrant

CONTENTS

1. Test set of molecules and ions used for evaluation of charge calculations 2
2. Observed and calculated charge values 4
3. Graphs of calculated versus observed charges 13
4. Data for fluorides XF_n^{m-}, Figure 1 14
5. Data for chlorides XCl_n^{m-}, Figure 2 25
6. Data for oxides XO_n^{m-}, Figure 3 and Table 2 32
7. Thermodynamic data for atoms and ions 42
8. Data for species in Table 3 43
9. Data for species in Scheme 5 48
10. Data for species in Scheme 7 54
11. Miscellaneous species mentioned in the text 61
12. Worked examples of γ calculations 63
13. Plots of γ(X) versus ΔG(X-O) for different charge schemes 72

1. Test Set of Molecules and Ions used for Evaluation of Charge Calculations

References are given in square brackets.

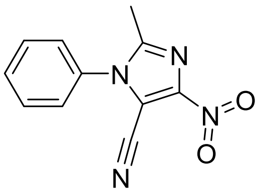

1 [11a]

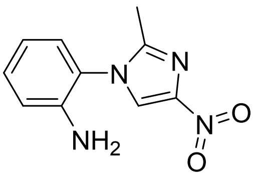

2 [11b]

3 K_2SO_4 [9]

4 SO_2 [11c]

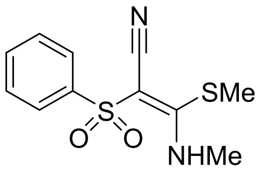

5 [11c]

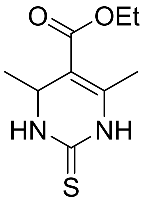

6 [11d]

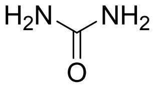

7 [11e]

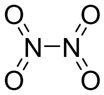

8 [11f]

9 $KMnO_4$ [11g]

10 $KClO_4$ [11g]

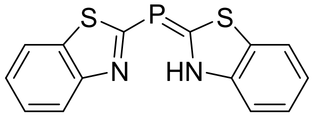

11 [11h]

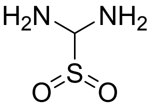

12 [11i]

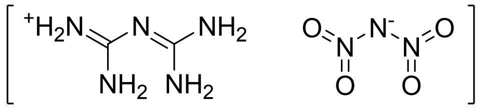

13 [11j]

Note; Geometry optimization of **13** from the X-ray crystal structure gave an imaginary frequency. Therefore, the calculated geometries were obtained from a separately constructed model which eliminated the imaginary frequency. The crystal structure is compared with the optimized geometries in Figure S1 below.

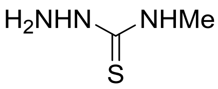

14 [11k]

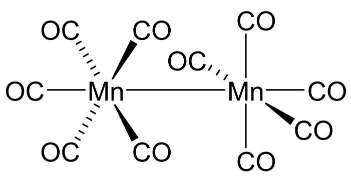

15 [11l]

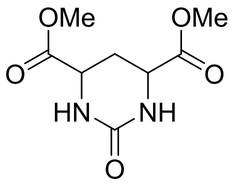

16 [11m]

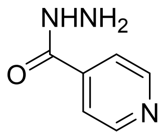

17 [11n]

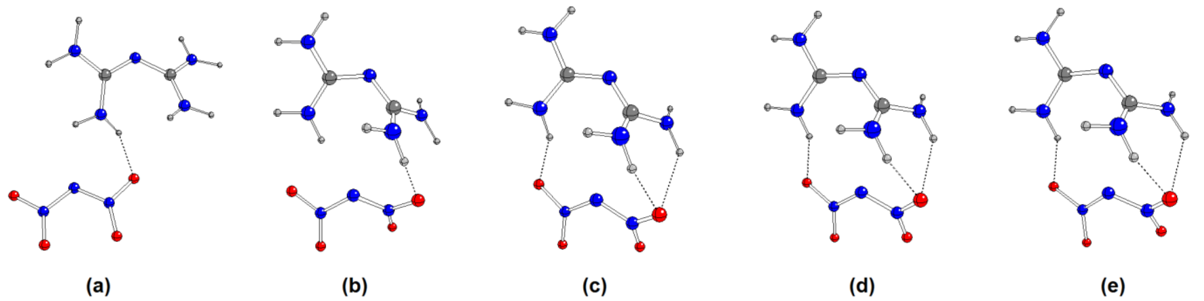

Fig. S1 Experimental and theoretical structures of molecule **13**; (a) X-ray geometry; (b) method 1; (c) method 5; (d) method 7; (e) method 8.

2. Observed and Calculated Charge Values

Quantum methods are defined in Table 1. Columns 'expt. charge', ' expt. charge 1' and 'expt. charge 2' are experimental values; 'mean expt. charge' is the mean of the experimental values (where applicable).

2.1 Molecule 1

atom	expt. charge	method 1	method 2	method 3	method 4	method 5	method 6	method 7	method 8
C3	0.009	-0.006	0.012	0.017	0.006	-0.033	0.001	-0.032	-0.049
C2	0.108	-0.003	0.012	0.018	0.015	-0.020	0.025	-0.018	-0.033
C1	0.175	0.315	0.322	0.313	0.315	0.251	0.257	0.250	0.254
C6	-0.053	0.001	0.016	0.023	0.019	-0.015	0.032	-0.015	-0.028
C5	0.066	-0.003	0.015	0.020	0.009	-0.030	0.005	-0.030	-0.045
C4	0.167	-0.009	0.009	0.014	0.006	-0.042	-0.018	-0.040	-0.052
N1	-1.192	-1.227	-1.211	-1.172	-1.244	-1.200	-1.454	-1.140	-1.155
C7	0.720	1.002	0.978	0.953	0.898	0.966	1.102	0.925	0.866
N2	-0.957	-1.097	-1.061	-1.039	-0.916	-1.105	-1.260	-1.066	-1.016
C8	0.623	0.792	0.771	0.755	0.648	0.825	0.992	0.800	0.791
C9	0.304	0.472	0.475	0.468	0.507	0.483	0.614	0.470	0.456
C91	0.844	0.923	0.941	0.929	0.872	0.915	0.988	0.896	0.771
N91	-1.160	-1.080	-1.105	-1.095	-1.031	-1.110	-1.238	-1.089	-0.948
N8	0.189	0.405	0.447	0.445	0.322	0.390	0.355	0.364	0.340
O81	-0.390	-0.436	-0.455	-0.453	-0.387	-0.432	-0.465	-0.421	-0.400
O82	-0.384	-0.467	-0.485	-0.483	-0.441	-0.462	-0.504	-0.449	-0.434
C71	0.233	0.015	0.085	0.101	0.110	-0.035	0.117	-0.027	-0.052
H3	0.156	0.046	0.027	0.022	0.035	0.082	0.059	0.079	0.094
H2	0.055	0.052	0.033	0.027	0.042	0.087	0.065	0.084	0.102
H6	0.118	0.060	0.042	0.035	0.049	0.095	0.073	0.089	0.110
H5	0.120	0.049	0.030	0.025	0.037	0.084	0.061	0.080	0.097
H4	-0.008	0.045	0.026	0.021	0.034	0.080	0.058	0.078	0.092
H73	0.116	0.069	0.044	0.037	0.053	0.093	0.065	0.089	0.099
H71	0.065	0.043	0.019	0.013	0.025	0.067	0.037	0.062	0.072
H72	0.079	0.039	0.016	0.010	0.020	0.064	0.033	0.061	0.068

2.2 Molecule 2

atom	expt. charge 1	expt. charge 2	mean expt. charge	method 1	method 2	method 3	method 4	method 5	method 6	method 7	method 8
C1	0.180	0.209	0.195	0.322	0.323	0.315	0.311	0.301	0.358	0.291	0.281
C2	-0.187	-0.203	-0.195	-0.004	0.013	0.018	0.026	-0.033	-0.009	-0.030	-0.032
C3	-0.064	0.088	0.012	-0.016	-0.001	0.004	-0.013	-0.027	0.037	-0.028	-0.056
C4	-0.061	-0.087	-0.074	-0.006	0.012	0.016	0.019	-0.048	-0.040	-0.045	-0.047
C5	0.020	0.027	0.024	-0.020	-0.006	0.001	-0.021	-0.024	0.043	-0.024	-0.054
C6	0.245	0.283	0.264	0.438	0.435	0.425	0.412	0.376	0.398	0.367	0.375
N1	-0.974	-0.933	-0.954	-1.258	-1.242	-1.203	-1.278	-1.232	-1.485	-1.172	-1.189
N6	-1.217	-1.200	-1.209	-1.132	-1.133	-1.096	-1.093	-1.096	-1.176	-1.065	-1.081
C7	0.650	0.705	0.678	0.982	0.957	0.933	0.872	0.948	1.087	0.907	0.849
N2	-0.736	-0.814	-0.775	-1.108	-1.074	-1.053	-0.933	-1.115	-1.269	-1.076	-1.026
C8	0.482	0.486	0.484	0.738	0.719	0.707	0.580	0.754	0.892	0.735	0.723
C9	0.150	0.177	0.164	0.410	0.422	0.416	0.458	0.363	0.473	0.353	0.323
N8	0.296	0.237	0.267	0.384	0.426	0.425	0.310	0.370	0.342	0.344	0.336
O82	-0.537	-0.539	-0.538	-0.449	-0.468	-0.466	-0.402	-0.446	-0.480	-0.435	-0.412
O81	-0.505	-0.458	-0.482	-0.491	-0.510	-0.508	-0.468	-0.487	-0.532	-0.476	-0.457
C71	-0.242	-0.345	-0.294	0.013	0.083	0.100	0.108	-0.038	0.110	-0.029	-0.055
H2	0.153	0.152	0.153	0.049	0.031	0.024	0.041	0.085	0.065	0.079	0.097
H3	0.166	0.127	0.147	0.036	0.017	0.012	0.027	0.072	0.049	0.069	0.088
H4	0.111	0.129	0.120	0.037	0.018	0.012	0.028	0.073	0.053	0.070	0.084
H5	0.149	0.131	0.140	0.028	0.009	0.003	0.020	0.064	0.039	0.059	0.081
H61	0.553	0.526	0.540	0.401	0.405	0.392	0.396	0.390	0.390	0.382	0.391
H62	0.538	0.514	0.526	0.393	0.397	0.385	0.387	0.385	0.385	0.377	0.385
H9	0.250	0.246	0.248	0.126	0.109	0.103	0.139	0.163	0.160	0.158	0.175
H72	0.177	0.180	0.179	0.037	0.013	0.007	0.017	0.061	0.029	0.056	0.066
H73	0.163	0.170	0.167	0.029	0.006	-0.001	0.010	0.053	0.020	0.049	0.061
H71	0.172	0.273	0.223	0.062	0.037	0.031	0.046	0.087	0.058	0.083	0.093

2.3 Molecule 3

atom	expt. charge 1	expt. charge 2	mean expt. charge	method 1	method 2	method 3	method 4	method 5	method 6	method 7	method 8
S	4.27		4.270	3.745	3.858	3.799	3.307	3.871	4.226	3.716	3.798
O1,2<br>O3	-1.37<br>-1.39	-1.43	-1.405	-1.393	-1.421	-1.402	-1.308	-1.441	-1.536	-1.398	-1.423
K1/2	0.72	0.77	0.745	0.913	0.914	0.904	0.962	0.946	0.958	0.939	0.947

2.4 Molecule 4

atom	expt. charge	method 1	method 2	method 3	method 4	method 5	method 6	method 7	method 8
S	2.57	2.291	2.407	2.368	2.122	2.374	2.638	2.290	2.175
O	-1.22	-1.146	-1.204	-1.184	-1.061	-1.187	-1.319	-1.145	-1.087

2.5 Molecule 5

atom	expt. charge	method 1	method 2	method 3	method 4	method 5	method 6	method 7	method 8
S1	2.358	2.470	2.592	2.553	2.357	2.534	(a)	2.430	2.693
S2	0.144	0.112	0.109	0.117	0.236	0.110		0.105	0.157
O1	-1.220	-1.315	-1.356	-1.337	-1.240	-1.346		-1.305	-1.447
O2	-1.359	-1.287	-1.333	-1.314	-1.206	-1.318		-1.278	-1.425
N1	-1.287	-1.155	-1.180	-1.167	-1.110	-1.185		-1.164	-1.236
N2	-1.258	-1.195	-1.187	-1.149	-1.195	-1.172		-1.131	-1.322
C1	0.042 (b)	-0.110	-0.121	-0.109	-0.204	-0.020		-0.009	0.081
C2	0.643	0.452	0.446	0.427	0.366	0.400		0.391	0.387
C3	0.842	0.878	0.897	0.881	0.826	0.851		0.830	0.825
C4	0.049	-0.102	-0.027	-0.015	-0.079	-0.151		-0.144	-0.047
C5	0.608	0.356	0.425	0.426	0.442	0.278		0.271	0.436
C6	-0.217	-0.164	-0.173	-0.170	-0.285	-0.196		-0.181	-0.236
C7	0.046	-0.005	0.013	0.018	0.037	-0.033		-0.027	-0.005
C8	0.154	-0.011	0.005	0.010	0.001	-0.039		-0.037	-0.012
C9	-0.172	-0.016	0.003	0.007	0.004	-0.054		-0.050	-0.043
C10	-0.010	-0.006	0.010	0.015	0.006	-0.033		-0.032	-0.005
C11	0.145	0.004	0.020	0.026	0.046	-0.021		-0.015	0.011

Notes:

(a) This molecule proved to be intractable using Method 6.

(b) The experimental value for C1 of -0.916 in Table 7 of the Supporting Information for reference [11c] is incorrect; the correct value is +0.042.
(S. Grabowsky, personal communication).

2.6 Molecule 6

atom	expt. charge	method 1	method 2	method 3	method 4	method 5	method 6	method 7	method 8
S	0.01	-0.002	-0.024	-0.010	-0.052	-0.021	0.007	-0.018	0.045
O1	-1.17	-1.179	-1.213	-1.193	-1.144	-1.181	-1.307	-1.157	-1.289
O2	-1.13	-1.114	-1.151	-1.122	-1.098	-1.114	-1.278	-1.071	-1.254
N1	-1.12	-1.194	-1.181	-1.139	-1.187	-1.171	-1.356	-1.130	-1.331
N3	-1.10	-1.165	-1.154	-1.117	-1.155	-1.144	-1.323	-1.100	-1.291
C2	0.68	0.638	0.634	0.601	0.612	0.645	0.786	0.614	0.713
C4	0.28	0.392	0.422	0.415	0.455	0.357	0.473	0.341	0.464
C4'	-0.21	-0.002	0.062	0.077	0.067	-0.072	0.052	-0.063	0.042
C5	-0.13	-0.008	-0.026	-0.016	-0.043	0.072	0.176	0.070	0.151
C5'	1.27	1.540	1.603	1.560	1.451	1.525	1.700	1.465	1.658
C6	0.24	0.405	0.408	0.392	0.414	0.303	0.264	0.305	0.283
C6'	0.08	0.018	0.085	0.103	0.103	-0.036	0.109	-0.029	0.099
C7	0.23	0.460	0.533	0.527	0.501	0.412	0.534	0.396	0.509
C8	0.05	0.022	0.085	0.099	0.092	-0.051	0.077	-0.044	0.069
H1	0.49	0.426	0.433	0.419	0.443	0.420	0.431	0.411	0.428
H3	0.43	0.420	0.425	0.410	0.431	0.414	0.421	0.404	0.419
H4	0.10	0.056	0.031	0.026	0.035	0.083	0.056	0.080	0.063
H41'	0.15	0.031	0.009	0.003	0.010	0.057	0.021	0.055	0.021
H42'	0.15	0.004	-0.018	-0.024	-0.017	0.029	-0.011	0.024	-0.007
H43'	0.16	0.010	-0.013	-0.019	-0.012	0.035	-0.006	0.031	-0.001
H61'	0.04	0.055	0.030	0.022	0.041	0.081	0.053	0.074	0.054
H62'	0.01	0.009	-0.013	-0.020	-0.011	0.034	-0.001	0.029	0.003
H63'	0.04	0.062	0.037	0.030	0.052	0.084	0.057	0.082	0.065
H71	0.11	0.041	0.015	0.012	0.019	0.065	0.035	0.064	0.041
H72	0.12	0.038	0.013	0.009	0.016	0.063	0.032	0.061	0.039
H81	0.07	0.014	-0.010	-0.015	-0.004	0.039	0.001	0.036	0.005
H82	0.06	0.010	-0.013	-0.018	-0.008	0.036	-0.003	0.033	0.000
H83	0.07	0.014	-0.009	-0.014	-0.011	0.040	0.000	0.037	0.002

2.7 Molecule 7

atom	expt. charge	method 1	method 2	method 3	method 4	method 5	method 6	method 7	method 8
O	-1.177	-1.201	-1.235	-1.213	-1.152	-1.205	-1.325	-1.181	-1.164
C	1.667	1.862	1.884	1.835	1.691	1.846	2.114	1.789	1.796
N	-1.214	-1.128	-1.130	-1.091	-1.070	-1.101	-1.181	-1.070	-1.106
H1	0.482	0.410	0.414	0.401	0.412	0.400	0.403	0.393	0.404
H2	0.493	0.387	0.391	0.378	0.388	0.381	0.383	0.373	0.386

2.8 Molecule 8

atom	expt. charge	method 1	method 2	method 3	method 4	method 5	method 6	method 7	method 8
N	0.72	0.746	0.782	0.771	0.613	0.742	0.797	0.708	0.656
O	-0.36	-0.373	-0.391	-0.385	-0.306	-0.371	-0.398	-0.354	-0.328

2.9 Molecule 9

atom	expt. charge	mean expt. charge	method 1	method 2	method 3	method 4	method 5	method 6	method 7	method 8
K	0.90	0.900	0.928	0.929	0.923	0.967	0.962	0.968	0.958	0.958
Mn	1.60	1.600	1.917	2.043	1.996	2.026	1.958	2.224	1.915	1.948
O2	-0.70	-0.625	-0.615	-0.650	-0.639	-0.647	-0.632	-0.701	-0.624	-0.532
O3	-0.60	-0.625	-0.807	-0.837	-0.820	-0.850	-0.829	-0.895	-0.813	-0.920

2.10 Molecule 10

atom	expt. charge	mean expt. charge	method 1	method 2	method 3	method 4	method 5	method 6	method 7	method 8
K	0.7	0.700	0.938	0.938	0.931	0.973	0.962	0.970	0.956	0.940
Cl	2.2	2.200	2.627	2.731	2.672	2.191	2.485	2.545	2.214	2.553
O1	-0.8	-0.725	-0.904	-0.907	-0.889	-0.750	-0.840	-0.862	-0.756	-0.854
O2, O3	-0.7	-0.725	-0.879	-0.928	-0.913	-0.833	-0.884	-0.896	-0.830	-0.893

2.11 Molecule 11

atom	expt. charge	method 1	method 2	method 3	method 4	method 5	method 6	method 7	method 8
S1	0.203	0.219	0.223	0.225	0.386	0.231	(a)	0.220	0.249
S2	0.070	0.171	0.172	0.177	0.320	0.181		0.168	0.223
P1	0.636	1.030	1.042	1.047	0.993	0.988		0.990	1.040
N1	-1.133	-1.242	-1.235	-1.194	-1.245	-1.202		-1.160	-1.168
N2	-1.210	-1.194	-1.165	-1.141	-1.097	-1.197		-1.159	-1.106
C1	-0.057	-0.209	-0.226	-0.243	-0.283	-0.202		-0.211	-0.273
C2	-0.157	-0.155	-0.157	-0.155	-0.280	-0.183		-0.174	-0.196
C3	-0.092	0.008	0.028	0.032	0.050	-0.009		-0.008	-0.027
C4	-0.148	-0.009	0.005	0.010	0.001	-0.032		-0.032	-0.054
C5	-0.163	-0.006	0.011	0.014	0.012	-0.036		-0.035	-0.046
C6	-0.136	-0.002	0.013	0.018	0.018	-0.005		-0.007	-0.034
C7	0.314	0.412	0.412	0.403	0.397	0.351		0.350	0.364
C8	0.046	-0.046	-0.066	-0.080	-0.182	-0.027		-0.046	-0.145
C9	-0.148	-0.178	-0.180	-0.178	-0.304	-0.217		-0.204	-0.226
C10	-0.080	0.002	0.022	0.027	0.042	-0.017		-0.015	-0.034
C11	-0.171	-0.014	0.002	0.006	-0.001	-0.042		-0.042	-0.059
C12	-0.133	-0.016	0.002	0.006	-0.002	-0.047		-0.045	-0.057
C13	-0.098	-0.014	0.003	0.008	0.012	-0.032		-0.030	-0.049
C14	0.283	0.421	0.412	0.403	0.363	0.407		0.400	0.396
H1	0.778	0.517	0.526	0.510	0.565	0.498		0.487	0.508
H3	0.163	0.047	0.028	0.022	0.040	0.083		0.078	0.094
H4	0.180	0.037	0.018	0.012	0.027	0.073		0.069	0.085
H5	0.161	0.037	0.018	0.012	0.028	0.073		0.069	0.083
H6	0.188	0.045	0.026	0.020	0.037	0.081		0.076	0.095
H10	0.164	0.041	0.022	0.016	0.034	0.077		0.072	0.090
H11	0.194	0.030	0.012	0.005	0.020	0.066		0.062	0.080
H12	0.177	0.029	0.010	0.004	0.019	0.065		0.061	0.078
H13	0.181	0.038	0.018	0.012	0.030	0.073		0.068	0.090

Note: (a) This molecule proved to be intractable using Method 6.

2.12 Molecule 12

atom	expt. charge	method 1	method 2	method 3	method 4	method 5	method 6	method 7	method 8
S	1.759	2.072	2.180	2.139	1.979	2.153	2.420	2.117	2.162
O	-1.255	-1.289	-1.337	-1.315	-1.226	-1.328	-1.452	-1.265	-1.212
C	0.893	1.142	1.131	1.110	1.043	1.131	1.275	1.084	0.993
N	-1.452	-1.209	-1.221	-1.184	-1.219	-1.183	-1.292	-1.166	-1.194
H1	0.671	0.414	0.419	0.404	0.431	0.407	0.417	0.387	0.386
H2	0.707	0.476	0.483	0.470	0.502	0.461	0.480	0.443	0.442

2.13 Molecule 13

atom	expt. charge 1	expt. charge 2	mean expt. charge	method 1	method 2	method 3	method 4	method 5	method 6	method 7	method 8
N1	-0.229	-0.092	-0.161	-0.256	-0.248	-0.239	-0.225	-0.224	-0.203	-0.206	-0.195
N2	0.648	0.639	0.644	0.667	0.704	0.692	0.555	0.655	0.687	0.622	0.638
N3	0.709	0.594	0.652	0.693	0.732	0.720	0.569	0.673	0.706	0.637	0.660
O2	-0.604	-0.525	-0.565	-0.597	-0.616	-0.608	-0.563	-0.585	-0.616	-0.569	-0.600
O1	-0.472	-0.575	-0.524	-0.450	-0.469	-0.467	-0.391	-0.440	-0.461	-0.424	-0.441
O3	-0.422	-0.639	-0.531	-0.534	-0.555	-0.549	-0.500	-0.555	-0.594	-0.543	-0.569
O4	-0.341	-0.590	-0.466	-0.445	-0.464	-0.460	-0.392	-0.437	-0.460	-0.424	-0.438
N6	-0.981	-1.134	-1.058	-1.219	-1.193	-1.166	-1.073	-1.259	-1.424	-1.220	-1.358
C1	1.385	1.296	1.341	1.604	1.571	1.532	1.411	1.613	1.856	1.558	1.803
C2	1.418	1.296	1.357	1.614	1.582	1.543	1.419	1.609	1.863	1.550	1.801
N5	-1.410	-1.167	-1.289	-1.168	-1.172	-1.134	-1.131	-1.139	-1.241	-1.103	-1.241
N4	-1.355	-1.287	-1.321	-1.156	-1.159	-1.121	-1.097	-1.117	-1.209	-1.086	-1.206
N7	-1.435	-1.167	-1.301	-1.146	-1.150	-1.113	-1.099	-1.114	-1.201	-1.081	-1.183
N8	-1.320	-1.287	-1.304	-1.188	-1.193	-1.153	-1.156	-1.160	-1.262	-1.127	-1.246
H3	0.636	0.623	0.630	0.471	0.479	0.466	0.486	0.459	0.475	0.452	0.478
H4	0.552	0.619	0.586	0.435	0.441	0.428	0.445	0.424	0.430	0.415	0.435
H2	0.568	0.635	0.602	0.492	0.500	0.486	0.507	0.462	0.477	0.454	0.483
H1	0.469	0.602	0.536	0.422	0.427	0.414	0.425	0.415	0.420	0.407	0.423
H6	0.591	0.668	0.630	0.411	0.417	0.404	0.415	0.401	0.406	0.393	0.406
H5	0.578	0.619	0.599	0.434	0.439	0.426	0.447	0.424	0.431	0.416	0.430
H8	0.536	0.635	0.586	0.418	0.422	0.409	0.431	0.401	0.407	0.392	0.410
H7	0.481	0.602	0.542	0.497	0.506	0.492	0.516	0.493	0.515	0.486	0.508

2.14 Molecule 14

atom	expt. charge 1	expt. charge 2	mean expt. charge	method 1	method 2	method 3	method 4	method 5	method 6	method 7	method 8
S1	-0.150	-0.200	-0.175	-0.052	-0.072	-0.060	-0.090	-0.069	-0.037	-0.064	-0.007
N1	-0.680	-0.810	-0.745	-0.674	-0.671	-0.650	-0.621	-0.661	-0.648	-0.652	-0.675
N2	-1.100	-0.980	-1.040	-0.799	-0.792	-0.764	-0.799	-0.784	-0.881	-0.752	-0.757
N3	-1.150	-1.160	-1.155	-1.198	-1.188	-1.150	-1.188	-1.180	-1.351	-1.140	-1.170
C1	0.910	0.860	0.885	0.671	0.670	0.638	0.631	0.675	0.807	0.640	0.582
C2	-0.100	0.080	-0.010	0.372	0.439	0.440	0.453	0.291	0.465	0.285	0.273
H2	0.470	0.450	0.460	0.415	0.420	0.405	0.425	0.404	0.413	0.393	0.400
H3	0.440	0.400	0.420	0.425	0.429	0.415	0.446	0.422	0.433	0.413	0.435
H12	0.400	0.410	0.405	0.364	0.363	0.350	0.345	0.357	0.353	0.349	0.355
H11	0.360	0.380	0.370	0.371	0.370	0.357	0.356	0.367	0.365	0.360	0.371
H21	0.340	0.140	0.240	0.017	-0.008	-0.012	-0.006	0.041	0.006	0.039	0.048
H22	0.010	0.240	0.125	0.043	0.020	0.014	0.022	0.066	0.034	0.064	0.070
H23	0.300	0.170	0.235	0.044	0.021	0.015	0.023	0.071	0.041	0.065	0.073

2.15 Molecule 15

atom	expt. charge	mean expt. charge	method 1	method 2	method 3	method 4	method 5	method 6	method 7	method 8
Mn1	1.084	1.084	0.827	0.883	0.908	0.864	0.890	(a)	0.936	1.153
O1	-1.159	-1.159	-1.148	-1.187	-1.164	-1.127	-1.160		-1.139	-1.021
O2, O3, O4, O5	-1.183, -1.240, -1.232, -1.188	-1.211	-1.143	-1.180	-1.157	-1.129	-1.157		-1.136	-1.034
C1	0.941	0.941	0.987	1.014	0.989	0.993	0.984		0.955	0.692
C2, C3, C4, C5	1.017, 0.928, 1.049, 1.013	1.002	0.977	1.003	0.975	0.947	0.978		0.948	0.828

Note: (a) This molecule proved to be intractable using Method 6.

2.16 Molecule 16

atom	expt. charge	method 1	method 2	method 3	method 4	method 5	method 6	method 7	method 8
O1	-1.227	-1.202	-1.237	-1.216	-1.158	-1.202	-1.323	-1.179	-1.301
O2	-1.015	-1.109	-1.150	-1.120	-1.084	-1.111	-1.269	-1.073	-1.246
O3	-1.303	-1.165	-1.201	-1.181	-1.123	-1.168	-1.293	-1.148	-1.273
O4	-1.063	-1.108	-1.149	-1.119	-1.082	-1.111	-1.269	-1.070	-1.244
O5	-1.349	-1.166	-1.202	-1.182	-1.125	-1.170	-1.296	-1.151	-1.276
N1	-1.222	-1.170	-1.166	-1.127	-1.159	-1.150	-1.325	-1.103	-1.296
N2	-1.224	-1.145	-1.140	-1.102	-1.125	-1.124	-1.291	-1.087	-1.256
C1	1.901	1.882	1.903	1.851	1.754	1.871	2.166	1.809	2.106
C2	0.200	0.389	0.415	0.413	0.443	0.369	0.500	0.357	0.487
C3	-0.208	0.037	0.079	0.091	0.100	-0.003	0.093	0.004	0.085
C4	0.132	0.400	0.427	0.424	0.457	0.376	0.510	0.367	0.496
C5	1.622	1.581	1.642	1.604	1.470	1.566	1.739	1.524	1.697
C6	0.021	0.446	0.538	0.535	0.498	0.383	0.541	0.367	0.514
C7	1.516	1.555	1.617	1.580	1.440	1.541	1.707	1.500	1.662
C8	0.111	0.447	0.539	0.536	0.499	0.385	0.543	0.368	0.515
H01	0.607	0.436	0.442	0.429	0.457	0.427	0.439	0.418	0.437
H02	0.598	0.412	0.415	0.401	0.421	0.406	0.413	0.397	0.409
H2	0.166	0.046	0.020	0.014	0.028	0.065	0.033	0.071	0.044
H3A	0.127	0.050	0.025	0.018	0.039	0.077	0.049	0.068	0.052
H3B	0.106	0.034	0.009	0.003	0.015	0.061	0.027	0.057	0.035
H4	0.199	0.050	0.023	0.017	0.041	0.076	0.050	0.073	0.059
H6A	0.234	0.046	0.019	0.017	0.032	0.068	0.037	0.067	0.041
H6B	0.215	0.054	0.029	0.026	0.035	0.076	0.047	0.075	0.053
H6C	0.235	0.053	0.029	0.025	0.034	0.076	0.046	0.076	0.054
H8A	0.186	0.048	0.024	0.021	0.028	0.073	0.042	0.071	0.048
H8B	0.219	0.046	0.019	0.016	0.033	0.068	0.037	0.067	0.043
H8C	0.216	0.054	0.029	0.026	0.035	0.075	0.046	0.075	0.054

2.17 Molecule 17

atom	expt. charge	method 1	method 2	method 3	method 4	method 5	method 6	method 7	method 8
O1	-1.060	-1.169	-1.201	-1.181	-1.134	-1.171	-1.294	-1.147	-1.119
N1	-0.930	-1.157	-1.129	-1.107	-1.040	-1.152	-1.322	-1.117	-1.068
N2	-0.910	-0.821	-0.819	-0.787	-0.824	-0.810	-0.930	-0.778	-0.810
N3	-0.750	-0.637	-0.638	-0.617	-0.560	-0.622	-0.598	-0.615	-0.629
C1	-0.110	-0.017	-0.001	0.006	0.003	-0.033	0.022	-0.030	-0.055
C2	0.070	-0.031	-0.028	-0.024	-0.034	-0.055	-0.091	-0.043	-0.048
C3	-0.110	-0.044	-0.025	-0.016	-0.024	-0.068	-0.023	-0.063	-0.088
C4	0.290	0.543	0.548	0.538	0.481	0.510	0.613	0.492	0.456
C5	0.210	0.534	0.540	0.532	0.473	0.498	0.594	0.481	0.447
C6	1.120	1.439	1.468	1.431	1.336	1.424	1.621	1.374	1.368
H1	0.170	0.078	0.060	0.054	0.079	0.113	0.099	0.110	0.124
H2	0.560	0.408	0.412	0.399	0.417	0.402	0.406	0.393	0.411
H3	0.190	0.039	0.021	0.015	0.040	0.077	0.057	0.071	0.094
H4	0.170	0.045	0.025	0.019	0.046	0.079	0.062	0.075	0.091
H5	0.150	0.048	0.027	0.022	0.048	0.081	0.064	0.078	0.093
H3A	0.390	0.366	0.365	0.353	0.341	0.360	0.355	0.352	0.363
H3B	0.420	0.375	0.375	0.364	0.350	0.368	0.366	0.367	0.371

3. Graphs of Calculated Versus Observed Charges

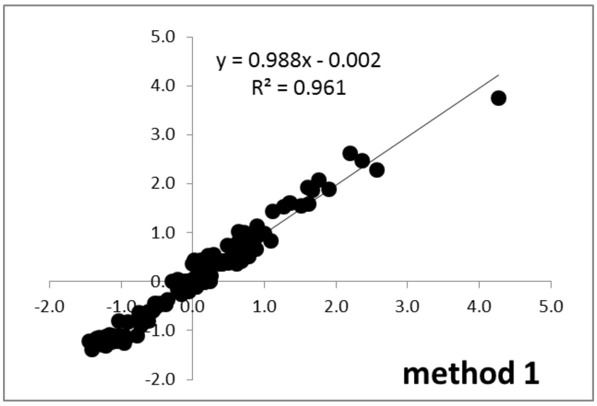

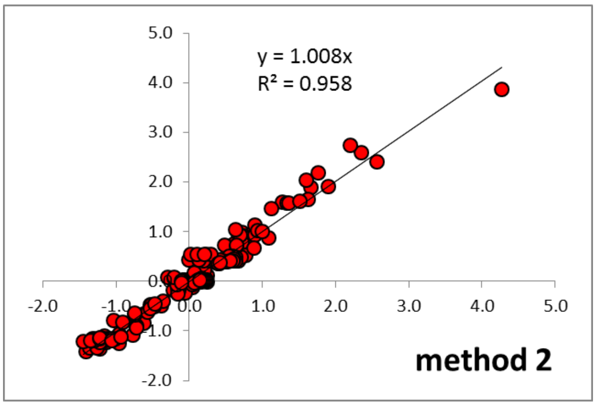

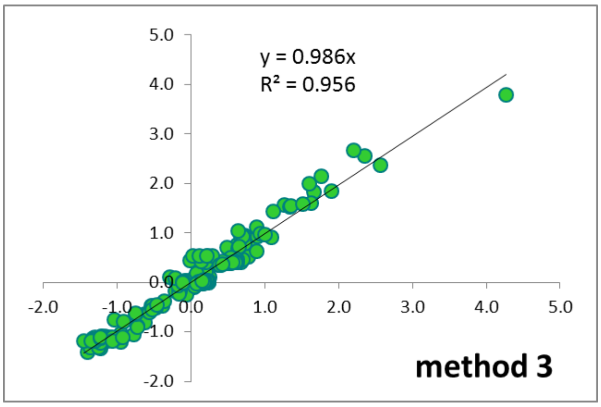

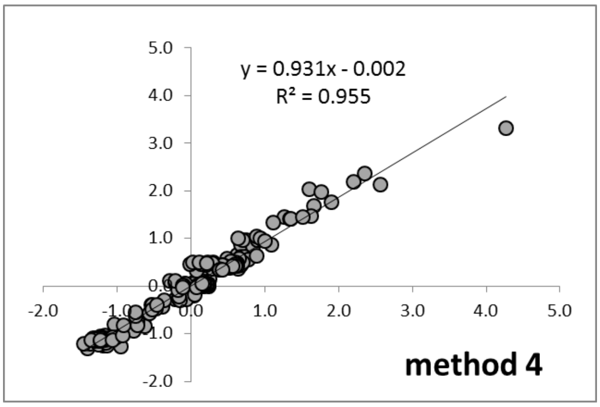

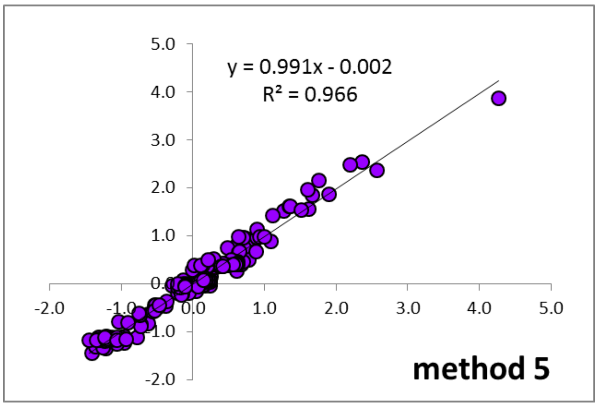

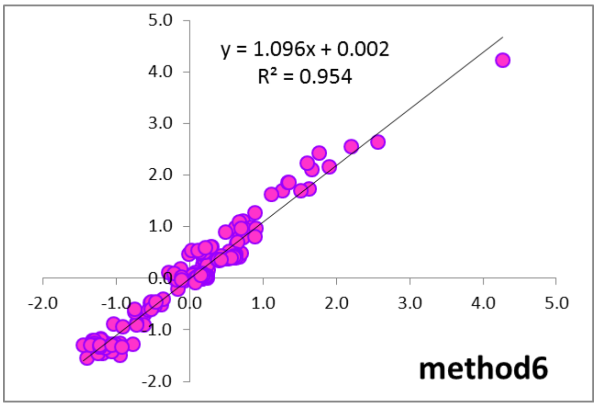

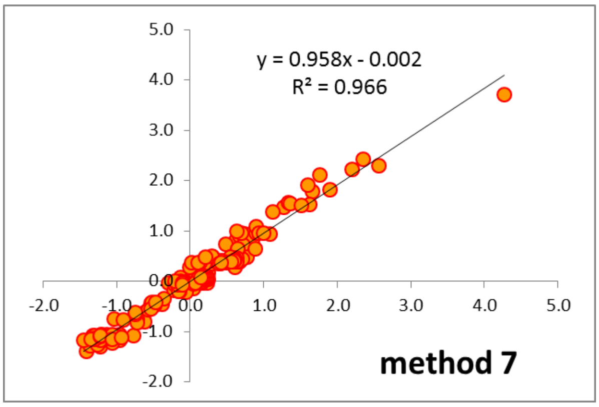

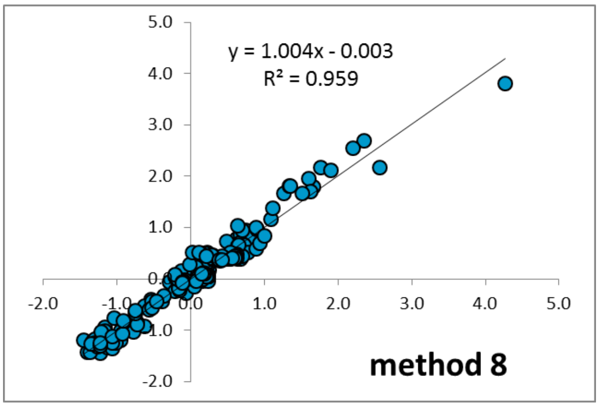

4. Data for fluorides XF_n^{m-}

Table S1. Data for graph shown in Fig. 1

species	explicit Rb	implicit Rb	ΔH kcal/mol	TΔS kcal/mol	ΔG (Rb.F) kcal/mol	ΔG (X-F) kcal/mol	γ(X)
XeF2	0	0	47.38	15.2	0	16.1	7.54
SF2	0	0	156.78	14.6	0	71.1	5.51
KrF2	0	0	12.08	15.4	0	-1.7	7.99
SeF2	0	0	151.89	14.4	0	68.8	5.67
Rb+ [F3]-	0	1	41.7	16.7	-3	11.1	8.11
Rb+ [ClF2]-	0	1	85.5	15.8	-3	33.4	7.42
Rb+ [BrF2]-	0	1	97.31	17.8	-3	38.3	7.19
Rb+ [IF2]-	0	1	110.38	15.7	-3	45.9	6.94
OF2	0	0	84	14.9	0	34.6	7.45
XeF3	0	0	50.29	20.8	0	9.8	8.11
PF3	0	0	329.94	23.8	0	102.1	3.29
SF3	0	0	204.98	22.6	0	60.8	5.37
ClF3	0	0	96.43	23.5	0	24.3	7.39
AsF3	0	0	300.57	23.2	0	92.4	4.06
SbF3	0	0	298.98	22.7	0	92.1	3.76
BrF3	0	0	126.33	23.2	0	34.4	6.86
IF3	0	0	162.62	22.0	0	46.9	6.29
AlF3	0	0	392.46	23.5	0	123.0	0.81
BF3	0	0	448.9	23.8	0	141.7	1.07
NF3	0	0	194.32	24.1	0	56.8	6.26
XeF4	0	0	84.03	30.6	0	13.4	7.45
Rb+ [AlF4]-	0	1	493.71	32.5	-3	114.6	0.78
Rb+ [PF4]-	0	1	363.96	33.0	-3	82.0	3.48
Rb+ [AsF4]-	0	1	345.04	32.2	-3	77.5	4.08
Rb+ [SbF4]-	0	1	357.36	31.6	-3	80.7	3.79
SiF4	0	0	521.99	33.3	0	122.2	1.34
GeF4	0	0	415.05	32.0	0	95.8	2.84
SF4	0	0	283.45	33.0	0	62.6	4.97
SeF4	0	0	286.31	32.4	0	63.5	5.25
TeF4	0	0	315.8	31.8	0	71.0	4.72
Rb+ [ClF4]-	0	1	145.47	32.3	-3	27.6	7.50
Rb+ [BrF4]-	0	1	185.16	32.5	-3	37.5	6.90
Rb+ [IF4]-	0	1	226.01	32.2	-3	47.8	6.26
Rb+ [BF4]-	0	1	515.95	33.7	-3	119.8	1.05
CF4	0	0	458.67	34.3	0	106.1	2.91
ClF5	0	0	123.93	42.9	0	16.2	8.10
PF5	0	0	493.05	42.8	0	90.1	2.29
AsF5	0	0	418	42.0	0	75.2	3.92
SbF5	0	0	424.53	40.7	0	76.8	3.44
SF5	0	0	312.41	43.0	0	53.9	5.07

Table S1 continued

species	explicit Rb	implicit Rb	ΔH kcal/mol	TΔS kcal/mol	ΔG (Rb.F) kcal/mol	ΔG (X-F) kcal/mol	γ(X)
Rb+ [SF5]-	0	1	328.61	42.1	-3	56.7	5.47
Rb+ [SeF5]-	0	1	346.47	42.4	-3	60.3	5.28
Rb+ [TeF5]-	0	1	388.83	41.8	-3	68.8	4.68
BrF5	0	0	188.3	42.6	0	29.1	7.02
IF5	0	0	267.15	41.8	0	45.1	5.94
Rb+ [XeF5]-	0	1	131.9	40.0	-3	17.8	7.55
XeF6	0	0	105.51	47.0	0	9.7	7.72
Rb+ [PF6]-	0	1	572.61	55.1	-3	85.8	2.30
Rb+ [AsF6]-	0	1	507.36	54.1	-3	75.1	3.77
Rb+[SbF6]-	0	1	531.61	52.6	-3	79.4	3.25
Rb+ Rb[SiF6]-	1	1	719.92	60.4	-4	109.2	1.30
Rb+ Rb[SnF6]-	1	1	639.82	45.8	-4	98.3	2.30
Rb+ Rb2[GaF6]-	2	1	673.14	42.2	-6	104.2	1.80
SF6	0	0	396.67	53.2	0	57.2	4.37
SeF6	0	0	382.59	54.1	0	54.7	5.37
TeF6	0	0	434.9	52.7	0	63.7	4.52
Rb+ [ClF6]-	1	1	173.84	46.9	-4	20.4	8.19
Rb+ Rb[SeF6]-	1	1	475.5	55.8	-4	69.2	5.26
Rb+ [BrF6]-	0	1	249.41	49.1	-3	32.9	7.09
Rb+ [IF6]-	0	1	323.74	45.6	-3	45.9	5.94
Rb+ Rb[XeF6]-	1	1	257.72	55.0	-4	33.1	7.57
IF7	0	0	311.82	60.3	0	35.9	6.25
Rb+ [TeF7]-	0	1	494.72	60.1	-3	61.7	4.56
Rb+ Rb[SbF7]-	1	1	650.82	67.6	-4	82.7	3.26
Rb+ [XeF7]-	0	1	172.36	58.0	-3	15.9	7.72
Rb+ Rb[XeF8]-	1	1	308.26	75.8	-4	28.5	7.77

Best fit parabolic curve:

$\gamma = 8.57 - 0.04735(\Delta G) - 0.0001364(\Delta G)^2$ RMS: 0.581

Data for individual fluorides

4.1 XeF_2
Geometry: linear, Xe-F 2.032 Å
Thermodynamics: H -7433.686202 Hartree S 61.839 cal $mol^{-1} K^{-1}$
Charges: Xe +1.230 F -0.615
Reference: H. St. A. Elliott *et al., Inorg. Chem.* 2010, **49**, 8504–8523.

4.2 SF_2
Geometry: F-S-F angle 98.5°, S-F 1.619 Å
Thermodynamics: H -597.736485 Hartree S 61.748 cal $mol^{-1} K^{-1}$
Charges: S +1.244 F -0.622
Reference: B. Brupbacher-Gatehouse, *J. Mol. Struct.*, 2001, **599**, p. 51 - 55.

4.3 KrF_2
Geometry: linear, Kr-F 1.903 Å
Thermodynamics: H -2952.762186 Hartree S 59.701 cal $mol^{-1} K^{-1}$

Charges:	QTAIM	NBO	Hirshfeld	Mulliken
Kr	+1.003	+1.027	+0.441	+0.828
F	-0.502	-0.514	-0.221	-0.414

Reference: C. Murchison *et al., J. Am. Chem. Soc.,* 1968 *,* **90***,* 5690 - 5690.

4.4 SeF_2
Geometry: F-Se-F angle 97.9°, Se-F 1.772 Å
Thermodynamics: H -2600.747516 Hartree S 65.310 cal $mol^{-1} K^{-1}$
Charges: Se +1.165 F -0.583
Reference: G. D. Brabson *et al.*, *Chem. Phys. Lett.*, 1996 , **254**, 94 - 97.

4.5 $[F_3]^-$
Geometry: linear, F-F 1.716 Å
Thermodynamics: H -299.365692Hartree S 58.763 cal $mol^{-1} K^{-1}$
Charges: central F -0.056 terminal F -0.472
Reference: B. S. Ault and L. Andrews, *J. Am. Chem. Soc.,* **98**, 1591-1593.

4.6 $[ClF_2]^-$
Geometry: linear, Cl-F 1.886 Å
Thermodynamics: H -659.798921 Hartree S 58.638 cal $mol^{-1} K^{-1}$
Charges: Cl +0.291 F -0.645
Reference: K. O. Christie and J. P. Guertin, *Inorg. Chem.*, 1965 , **4**, p. 905 - 908.

4.7 $[BrF_2]^-$
Geometry: linear, Br-F 1.995 Å
Thermodynamics: H -2773.408934 Hartree S 54.335 cal $mol^{-1} K^{-1}$
Charges: Br +0.405 F -0.702
Reference: R. Minkwitz *et al., Inorg. Chem.,* 1997 *,* **36***,* 4280 - 4283.

4.8 $[IF_2]^-$
Geometry: linear, I-F 2.131 Å
Thermodynamics: H -7119.739523 Hartree S 62.844 cal $mol^{-1} K^{-1}$
Charges: +0.531 F -0.865
Reference: K. O. Christe, *J. Am. Chem. Soc.,* 1998 , **120**, 4711 - 4716.

4.9 OF_2
Geometry: F-O-F angle 103.9°, O-F 1.394 Å
Thermodynamics: H -274.623018 Hartree S 58.845 cal $mol^{-1} K^{-1}$
Charges: O 0.273 F -0.136
(well known)

4.10 XeF_3
Geometry: T-shaped, F-Xe-F angle 90.3^o,
Xe-F (central) 2.427 Å, Xe-F (outer) 2.015 Å
Thermodynamics: H -7533.409379 Hartree S 90.251 cal mol^{-1} K^{-1}
Charges: Xe +1.444 F(central) -0.256 F(outer) -0.594
Reference: E. Y. Misochko *et al., Inorg. Chem.,* 2009, **48**, 8723 - 8728.

4.11 PF_3
Geometry: pyramidal, F-P-F 97.2^o, P-F 1.597 Å
Thermodynamics: H -640.884995 Hartree S 67.763 cal mol^{-1} K^{-1}
Charges: P +2.353 F -0.784
(well known)

4.12 SF_3
Geometry: T-shaped, F-S-F angle 88.0^o, S-F (central) 1.598 Å, S-F (outer) 1.691 Å
Thermodynamics: H -697.531833 Hartree S 71.099 cal mol^{-1} K^{-1}
Charges: S +1.816 F(central) -0.608 F(outer) -0.604
Reference: T. Kiang and R. N. Zare, *J. Am. Chem. Soc.,* 1980, **102**, 4024-4029.

4.13 ClF_3
Geometry: T-shaped, F-Cl-F angle 87.2^o, Cl-F (central) 1.633 Å, Cl-F (outer) 1.732 Å
Thermodynamics: H -759.393001 Hartree S 67.653 cal mol^{-1} K^{-1}
Charges: Cl +1.304 F(central) -0.336 F(outer) -0.484
(well known)

4.14 AsF_3
Geometry: pyramidal, F-As-F angle 96.1^o, As-F 1.753 Å
Thermodynamics: H -2535.035168 Hartree S 72.123 cal mol^{-1} K^{-1}
Charges: As +1.970 F -0.656
(well known)

4.15 SbF_3
Geometry: pyramidal, F-Sb-F angle 94.4^o, Sb-F 1.942 Å
Thermodynamics: H -6615.021862 Hartree S 75.427 cal mol^{-1} K^{-1}
Charges: Sb +2.119 F -0.707
(well known)

4.16 BrF_3
Geometry: T-shaped, F-Br-F 86.2^o, Br-F (central) 1.767 Å, Br-F (outer) 1.847 Å
Thermodynamics: H -2873.029486 Hartree S 70.959 cal mol^{-1} K^{-1}
Charges: Br +1.569 F(central) -0.442 F(outer) -0.564
(well known)

4.17 IF_3
Geometry: T-shaped, F-I-F 83.4^o, I-F (central) 1.930 Å, I-F (outer) 1.994 Å
Thermodynamics: H -7219.397077 Hartree S 73.466 cal mol^{-1} K^{-1}
Charges: I +1.854 F(central) -0.564 F(outer) -0.644
Reference: S. Hoyer and K. Seppelt, *Angew. Chem. Int. Ed.*, 2000, **39**, 1448-1449.

4.18 AlF_3
Geometry: trigonal planar, Al-F 1.659 Å
Thermodynamics: H -542.093337 Hartree S 66.698 cal mol^{-1} K^{-1}
Charges: Al +2.593 F -0.865
(well known)

4.19 BF_3
Geometry: trigonal planar, B-F 1.322 Å
Thermodynamics: H -324.513652 Hartree S 63.136 cal mol^{-1} K^{-1}
Charges: B +2.463 F -0.821
(well known)

4.20 NF_3
Geometry: pyramidal, F-N-F 102.1°, N-F 1.371 Å
Thermodynamics: H -354.038067 Hartree S 64.322 cal mol^{-1} K^{-1}
Charges: N +0.870 F -0.290
(well known)

4.21 XeF_4
Geometry: square planar, Xe-F 2.001 Å
Thermodynamics: H -7633.182049 Hartree S 82.668 cal mol^{-1} K^{-1}
Charges: Xe +2.273 F -0.568
(well known)

4.22 $[AlF_4]^-$
Geometry: tetrahedral, Al-F 1.717 Å
Thermodynamics: H -642.117464 Hartree S 74.062 cal mol^{-1} K^{-1}
Charges: Al +2.612 F -0.903
(well known)

4.23 $[PF_4]^-$
Geometry: seesaw, F-As-F (ax-eq) 86.7°, (eq-eq) 98.7°,
As-F (ax) 1.780 Å, As-F (eq) 1.647 Å
Thermodynamics: H -740.801974 Hartree S 74.383 cal mol^{-1} K^{-1}
Charges: P +2.260 F (ax) -0.820 F (eq) -0.809
Reference: K. O. Christe *et al., J. Am. Chem. Soc.,* 1994, **116**, 2850-2858.

4.24 $[AsF_4]^-$
Geometry: seesaw, F-As-F (ax-eq) 86.6°, (eq-eq) 99.2°
As-F (ax) 1.919 Å, As-F (eq) 1.798 Å
Thermodynamics: H -2634.968804 Hartree S 79.534 cal mol^{-1} K^{-1}
Charges: As +1.962 F(ax) -0.775 F(eq) -0.706
Reference: P. Klampfer *et al.*, *Coll. Czech. Chem. Comm.,* 2004, **69**, 339-350.

4.25 $[SbF_4]^-$
Geometry: seesaw, F-Sb-F (ax-eq) 83.3°, (eq-eq) 103.6°
Sb-F (ax) 2.051 Å, Sb-F (eq) 1.982 Å
Thermodynamics: H -6714.977668 Hartree S 82.977 cal mol^{-1} K^{-1}
Charges: Sb +2.103 F(ax) -0.795 F(eq) -0.756
Reference: V. Y. Kavun *et al.*, *Russ. J. Inorg. Chem.*, 2003, **48**, 874-879.

4.26 SiF_4
Geometry: tetrahedral, Si-F 1.582 Å
Thermodynamics: H -689.021694 Hartree S 71.065 cal mol^{-1} K^{-1}
Charges: Si +3.332 F -0.833
(well known)

4.27 GeF_4
Geometry: tetrahedral, Ge-F 1.721 Å
Thermodynamics: H -2476.061106 Hartree S 78.316 cal mol^{-1} K^{-1}
Charges: Ge +2.581 F -0.645
(well known)

4.28 SF_4
Geometry: seesaw, F-S-F (ax-eq) 87.5°, (eq-eq) 101.4°
S-F (ax) 1.681 Å, S-F (eq) 1.583 Å
Thermodynamics: H -797.375423 Hartree S 72.381 cal mol^{-1} K^{-1}
Charges: S +2.515 F(ax) -0.633 F(eq) -0.624
(well known)

4.29 SeF_4
Geometry: seesaw, F-Se-F (ax-eq) 86.5°, (eq-eq) 101.4°
Se-F (ax) 1.812 Å, Se-F (eq) 1.733 Å
Thermodynamics: H -2800.398804 Hartree S 77.104 cal mol^{-1} K^{-1}
Charges: Se +2.375 F(ax) -0.631 F(eq) -0.556
Reference: K. O. Christe *et al., J. Am. Chem. Soc.* 2001*,* **123***,* 6338-6348.

4.30 TeF_4
Geometry: seesaw, F-Te-F (ax-eq) 83.0°, (eq-eq) 106.7°
Te-F (ax) 1.959 Å, Se-F (eq) 1.914 Å
Thermodynamics: H -7013.118518 Hartree S 80.785 cal mol^{-1} K^{-1}
Charges: Te +2.641 F(ax) -0.682 F(eq) -0.639
Reference: S. A. Shlykov, *Dalton Trans.,* 2010, **39**, 3245-3255.

4.31 $[ClF_4]^-$
Geometry: square planar, Cl-F 1.900 Å
Thermodynamics: H -859.331558 Hartree S 75.458 cal mol^{-1} K^{-1}
Charges: Cl +1.249 F -0.562
Reference: K. O. Christe and J. P. Guertin, *Inorg. Chem.,* 1966, **5**, 473–476.

4.32 $[BrF_4]^-$
Geometry: square planar, Br-F 1.931 Å
Thermodynamics: H -2972.986018 Hartree S 77.329 cal mol^{-1} K^{-1}
Charges: Br +1.551 F -0.637
Reference: V. F. Sukhoverkhov and J. Moltasova, *Russ. J. Inorg. Chem.,* 1980, **25**, 1671 - 1673.

4.33 $[IF_4]^-$
Geometry: square planar, I-F 2.074 Å
Thermodynamics: H -7319.360866 Hartree S 79.915 cal mol^{-1} K^{-1}
Charges: I +1.872 F -0.718
Reference: K. O. Christe and D. Naumann, *Inorg. Chem.,* 1973, **12**, 59-62.

4.34 $[BF_4]^-$
Geometry: tetrahedral, B-F 1.417 Å
Thermodynamics: H -424.483280 Hartree S 67.359 cal mol^{-1} K^{-1}
Charges: B +2.475 F -0.869
(well known)

4.35 CF_4
Geometry: tetrahedral, C-F 1.325 Å
Thermodynamics: H -437.436947 Hartree S 65.248 cal mol^{-1} K^{-1}
Charges: C +2.545 F -0.636
(well known)

4.36 ClF_5
Geometry: square pyramidal, F-Cl-F (ax-eq) 85.8°
Cl-F (ax) 1.650 Å, Cl-F (eq) 1.704 Å
Thermodynamics: H -958.873904 Hartree S 74.796 cal mol^{-1} K^{-1}
Charges: Cl +1.948 F(ax) -0.301 F(eq) -0.412
Reference: G. M. Begun *et al., J. Chem. Phys,* 1965, **42**, 2236-2242.

4.37 PF_5
Geometry: trigonal bipyramidal,
P-F (ax) 1.598 Å, P-F (eq) 1.564 Å
Thermodynamics: H -840.581998 Hartree S 76.261 cal mol^{-1} K^{-1}
Charges: P +3.853 F(ax) -0.779 F(eq) -0.765
(well known)

4.38 AsF_5
Geometry: trigonal bipyramidal,
As-F (ax) 1.733 Å, As-F (eq) 1.711 Å
Thermodynamics: H -2734.659378 Hartree S 81.567 cal mol^{-1} K^{-1}
Charges: As +3.041 F(ax) -0.624 F(eq) -0.597
(well known)

4.39 SbF_5
Geometry: trigonal bipyramidal,
Sb-F (ax) 1.911 Å, Sb-F (eq) 1.902 Å
Thermodynamics: H -6814.659009 Hartree S 87.438 cal mol^{-1} K^{-1}
Charges: Sb +3.281 F(ax) -0.663 F(eq) -0.651
(well known)

4.40 SF_5
Geometry: square pyramidal, F-S-F (ax-eq) 91.6°
S-F (ax) 1.572 Å, S-F (eq) 1.642 Å
Thermodynamics: H -897.140110 Hartree S 74.925 cal mol^{-1} K^{-1}
Charges: S +2.964 F(ax) -0.638 F(eq) -0.581
Reference: P. Hassanzadeh and L. Andrews, *J. Phys. Chem.*, 1992, **96**, 79-84.

4.41 $[SF_5]^-$
Geometry: square pyramidal, F-S-F (ax-eq) 85.3°
S-F (ax) 1.626 Å, S-F (eq) 1.764 Å
Thermodynamics: H -897.310162 Hartree S 79.430 cal mol^{-1} K^{-1}
Charges: S +2.263 F(ax) -0.648 F(eq) -0.654
Reference: J. Bittner, *Z. Anorg. Allg. Chem.,* 1988, **557**, 182-190.

4.42 $[SeF_5]^-$
Geometry: square pyramidal, F-Se-F (ax-eq) 85.0°
Se-F (ax) 1.767 Å, Se-F (eq) 1.881 Å
Thermodynamics: H -2900.357439 Hartree S 81.298 cal mol^{-1} K^{-1}
Charges: Se +2.362 F(ax) -0.607 F(eq) -0.689
Reference: K. O. Christe *et al.*, *J. Fluor. Chem.,* 2010, **131**, 791-799.

4.43 $[TeF_5]^-$
Geometry: square pyramidal, F-Te-F (ax-eq) 82.0°
Te-F (ax) 1.939 Å, Te-F (eq) 2.019 Å
Thermodynamics: H -7113.097675 Hartree S 84.594 cal mol^{-1} K^{-1}
Charges: Te +2.658 F(ax) -0.690 F(eq) -0.742
Reference: W. Abriel, *Mater. Res. Bull.,* 1989, **24**, 1515-1519.

4.44 BrF_5
Geometry: square pyramidal, F-Br-F (ax-eq) 84.9°
Br-F (ax) 1.759 Å, Br-F (eq) 1.811 Å
Thermodynamics: H -3072.565323 Hartree S 78.222 cal mol^{-1} K^{-1}
Charges: Br +2.489 F(ax) -0.425 F(eq) -0.516
Reference: G. M. Begun *et al.*, *J. Chem. Phys.,* 1965, **42**, 2236-2242.

4.45 IF_5
Geometry: square pyramidal, F-I-F (ax-eq) 82.1°
I-F (ax) 1.903 Å, I-F (eq) 1.947 Å
Thermodynamics: H -7419.000736 Hartree S 82.223 cal mol^{-1} K^{-1}
Charges: I +3.029 F(ax) -0.566 F(eq) -0.615
Reference: G. M. Begun *et al.*, *J. Chem. Phys.,* 1965, **42**, 2236-2242.

4.46 $[XeF_5]^-$
Geometry: pentagonal planar, Xe-F 2.083 Å
Thermodynamics: H -7733.120742 Hartree S 88.438 cal mol^{-1} K^{-1}
Charges: Xe +2.226 F -0.645
Reference: K. O. Christe *et al., J. Am. Chem. Soc.* 1991*,* **113***,* 3351-3361.

4.47 XeF_6
Geometry: octahedral, Xe-F 1.998 Å
Thermodynamics: H -7832.652980 Hartree S 99.705 cal mol^{-1} K^{-1}
Charges: Xe +3.140 F -0.523
(well known)

4.48 $[PF_6]^-$
Geometry: octahedral, P-F 1.639 Å
Thermodynamics: H -940.571556 Hartree S 72.280 cal mol^{-1} K^{-1}

Charges:	QTAIM	NBO	Hirshfeld	Mulliken
P	+3.851	+2.795	+0.517	+1.548
F	-0.808	-0.633	-0.253	-0.425

(well known)

4.49 $[AsF_6]^-$
Geometry: octahedral, As-F 1.771 Å
Thermodynamics: H -2834.664556 Hartree S 78.527 cal mol^{-1} K^{-1}
Charges: As +3.118 F -0.686
(well known)

4.50 $[SbF_6]^-$
Geometry: octahedral, Sb-F 1.945 Å
Thermodynamics: H -6914.692415 Hartree S 84.970 cal mol^{-1} K^{-1}
Charges: Sb +3.374 F -0.729
(well known)

4.51 $[RbSiF_6]^-$
Cartesian coordinates:

Si	-0.575425	-0.321477	0.017761
F	-1.850296	-0.085600	-1.052285
F	0.843637	-0.400495	1.101168
F	0.562724	0.325741	-1.199788
F	-1.566302	-0.824669	1.279221
F	-0.254934	-1.883078	-0.515623
F	-0.729188	1.370840	0.572443
Rb	1.812902	1.923849	0.430858

Thermodynamics: H -3828.702666 Hartree S 94.539 cal mol^{-1} K^{-1}
Charges: Si +3.348 F -0.890 (x 3), -0.876 (x 3) Rb +0.953
Reference: B. Neumüller and K. Dehnicke, *Z. Anorg. Allg. Chem.,* 2008, **634**, 2567-2571.

4.52 $[RbSnF_6]^-$
Cartesian coordinates:

Sn	-0.614830	-0.353758	0.006779
F	-2.108853	-0.042585	-1.266979
F	1.042738	-0.395260	1.242537
F	0.744189	0.437527	-1.337444
F	-1.787031	-0.935670	1.502889
F	-0.232000	-2.196802	-0.631766
F	-0.707103	1.609904	0.650723
Rb	1.906008	1.981756	0.467016

Thermodynamics: H -9564.087522 Hartree S 106.895 cal mol^{-1} K^{-1}
Charges: Sn +2.850 F -0.823 (x 3), -0.778 (x 3) Rb +0.953
Reference: G. M. Begun and A. C. Rutenberg, *Inorg. Chem.,* 1967, **6**, 2212-2216.

4.53 $[Rb_2GaF_6]^-$

Cartesian coordinates:

```
Ga    -0.000070   -0.000591    0.000110
F      1.283129    0.687566    1.306897
F      0.962404   -1.702706    0.061065
F     -1.239426   -0.567076    1.405505
F     -0.961289    1.702482   -0.060865
F      1.238729    0.566364   -1.405792
F     -1.283749   -0.688325   -1.306277
Rb    -3.208514    0.413161    0.035876
Rb     3.208838   -0.410854   -0.036004
```

Thermodynamics: H -8403.523567 Hartree S 116.748 cal mol^{-1} K^{-1}

Charges: Ga +2.098 F -0.829 Rb +0.937

Reference: G. A. Kirakosyan *et al., Russ. J. Inorg. Chem.,* 1990*,* **35***,* 1314 - 1317.

4.54 SF_6

Geometry: octahedral, S-F 1.594 Å

Thermodynamics: H -996.992922 Hartree S 76.963 cal mol^{-1} K^{-1}

Charges: S +3.813 F -0.635

(well known)

4.55 SeF_6

Geometry: octahedral, Se-F 1.728 Å

Thermodynamics: H -2999.989311 Hartree S 76.516 cal mol^{-1} K^{-1}

Charges: Se +3.317 F -0.553

(well known)

4.56 TeF_6

Geometry: octahedral, Te-F 1.892 Å

Thermodynamics: H -7212.745396 Hartree S 82.903 cal mol^{-1} K^{-1}

Charges: Te +3.740 F -0.623

(well known)

4.57 $[ClF_6]^-$

Geometry: octahedral, Cl-F 1.807 Å

Thermodynamics: H -1058.813845 Hartree S 90.480 cal mol^{-1} K^{-1}

Charges: Cl +1.904 F -0.484

Reference: K. O. Christe *et al., Inorg. Chem.,* 1990, **29**, 3506-3511.

4.58 $[RbSeF_6]^-$

Cartesian coordinates:

```
Se    -0.006410   -1.014502    0.154743
F     -0.568679   -2.259380   -1.078417
F      0.644957    0.451551    1.586249
F      1.179710    0.000053   -1.324031
F     -1.028274   -1.871261    1.422921
F      1.390874   -2.133512    0.585119
F     -1.629612    0.297159   -0.346998
Rb     0.179075    2.233334   -0.315086
```

Thermodynamics: H -5940.065849 Hartree S 112.834 cal mol^{-1} K^{-1}

Charges: Se +2.376 F -0.789 (x 3), -0.650 (x 3) Rb +0.941

Reference: A. R. Mahjoub *et al.*, *Chem. Eur. J.,* 1995, 261-265.

4.59 $[BrF_6]^-$

Geometry: octahedral, Br-F 1.901 Å

Thermodynamics: H -3172.525476 Hartree S 93.923 cal mol^{-1} K^{-1}

Charges: Br +2.456 F -0.576

Reference: A. R. Mahjoub *et al.*, *Chem. Eur. J.,* 1995, 261-265.

4.60 $[IF_6]^-$
Geometry: octahedral, I-F 2.051 Å
Thermodynamics: H -7518.953699 Hartree S 106.981 cal mol^{-1} K^{-1}
Charges: I +3.032 F -0.672
Reference: A. R. Mahjoub and K. Seppelt, *Angew. Chem. Int. Edn. Engl*, 1991, **30**, 323-324.

4.61 $[RbXeF_6]^-$
Full geometry optimization resulted in dissociation of one F from Xe. A stable geometry could only be obtained using a z-matrix, final geometry as follows;
Xe
F1 Xe R1
F2 Xe R2 F1 A2
F3 Xe R3 F1 A3 F2 D3
F4 Xe R4 F1 A4 F2 D4
F5 Xe R5 F1 A5 F2 D5
F6 Xe R6 F1 A6 F2 D6
Rb Xe R9 F1 A9 F2 D9
optimized variables:
R1 2.066105, R2 2.060195, R3 2.081407, R4 2.179316,
R5 2.174397, R9 3.679195
A2 73.2623, A3 73.2405, A9 156.8631, D3 -180.1915, D9 79.7849,
A4 143.1718, A5 144.7949, D4 -18.9291, D5 -174.6872, A6 108.903
constants (taken from the optimized $[XeF]^{2-}$ ion):
R6 2.6265, D6 79.822
This geometry gave no negative IR frequencies.
Thermodynamics: H -10772.816027 Hartree S 112.297 cal mol^{-1} K^{-1}
Charges: Xe +2.213 F(ax) -0.892, F(eq) -0.693, -0.697, -0.637, -0.620, -0.616 Rb +0.941
Reference: A. I. Popov and Y. M. Kiselev, *Russ. J. Inorg. Chem.*, 1988, **33**, 1307-1309.

4.62 IF_7
Geometry: pentagonal bipyramidal, I-F (ax) 1.884 Å, I-F (eq) 1.935 Å
Thermodynamics: H -7618.509007 Hartree S 92.720 cal mol^{-1} K^{-1}
Charges: I +3.874 F(ax) -0.544 F(eq) -0.557
Reference: K. O. Christe *et al.*, *J. Am. Chem. Soc.,* 1993*,* **115***,* 1520-1526.

4.63 $[TeF_7]^-$
Geometry: pentagonal bipyramidal, Te-F (ax) 1.917 Å, I-F (eq) 1.970 Å
Thermodynamics: H -7312.703503 Hartree S 95.369 cal mol^{-1} K^{-1}
Charges: Te +3.720 F(ax) -0.666 F(eq) -0.678
Reference: K. O. Christe *et al.*, *J. Am. Chem. Soc.,* 1993, **115**, 9461-9467.

4.64 $[RbSbF_7]^-$
Cartesian coordinates:
```
Sb    0.166782   -0.071180    0.061854
F    -0.494827    1.078035    1.637328
F    -1.553500    0.745955   -0.721226
F     1.805599    0.116907    1.140574
F    -1.220407   -1.228818    0.848839
F     0.822059    1.799338   -0.494975
F     0.984041   -1.882891   -0.051902
F     0.454527   -0.304891   -1.873984
Rb   -1.290492    3.173895    0.258875
```
Thermodynamics: H -9954.385184 Hartree S 111.212 cal mol^{-1} K^{-1}
Charges: Sb +3.371 F -0.794 (x 3), -0.734 (x 3), -0.745 Rb +0.958
Reference: G. W. Drake *et al., J. Am. Chem. Soc.,* 1998, **120**, 8392-8400.

4.65 $[XeF_7]^-$
Geometry: pentagonal bipyramidal, Xe-F (ax) 2.032 Å, Xe-F (eq) 2.061 Å
Thermodynamics: H -7932.622294 Hartree S 100.584 cal mol^{-1} K^{-1}
Charges: Xe +3.140 F(ax) -0.573 F(eq) -0.599
Reference: A. Ellern, *Angew. Chem. Int. Edn. Engl.,* 1996, **35**, 1123-1125.

4.66 $[RbXeF_8]^-$
Cartesian coordinates:

Xe	-0.011562	-0.314763	-0.256325
F	-0.177725	-1.208185	-2.084251
F	-1.160831	0.735295	1.267541
F	1.330155	0.885373	0.970313
F	-1.472729	0.987165	-1.213417
F	-1.680149	-1.487474	-0.164188
F	1.019626	1.137805	-1.510504
F	1.758479	-1.279683	-0.578425
F	0.255974	-1.556453	1.341672
Rb	-0.171857	3.053472	0.104958

Thermodynamics: H -10972.341655 Hartree S 117.600 cal mol^{-1} K^{-1}
Charges: Xe +3.116 F -0.685 (x 4), -0.582 (x 4) Rb +0.956
Reference: K. O. Christe and W. W. Wilson, *Inorg. Chem.,* 1982, **21**, 4113–4117.

5. Data for chlorides XCl_n^{m-}

Table S2. Data for graph shown in Fig. 2

species	explicit Rb	implicit Rb	ΔH kcal/mol	TΔS kcal/mol	ΔG (Rb.Cl) kcal/mol	ΔG (X-Cl) kcal/mol	γ(X)
SCl2	0	0	110.03	14.1	0	48.0	7.30
OCl2	0	0	85.97	14.5	0	35.8	8.00
SeCl2	0	0	114.63	13.9	0	50.4	6.84
TeCl2	0	0	117.58	13.6	0	52.0	6.36
Rb+ [BrCl2]-	0	1	74.55	14.8	25	42.6	7.78
Rb+ [ICl2]-	0	1	81.58	14.7	25	46.2	7.48
XeCl2	0	0	9.01	14.3	0	-2.6	8.47
Rb+ [Cl3]-	0	1	65.47	14.2	25	38.3	8.04
PCl3	0	0	207.89	22.7	0	61.7	5.50
NCl3	0	0	130.39	23.1	0	35.8	8.00
BCl3	0	0	315.04	22.6	0	97.5	2.19
AsCl3	0	0	212.31	22.5	0	63.3	5.44
SbCl3	0	0	217.71	21.9	0	65.3	4.99
GaCl3	0	0	245.88	21.7	0	74.7	3.08
InCl3	0	0	233.96	21.0	0	71.0	2.81
AlCl3	0	0	291.65	21.7	0	90.0	1.33
SCl4	0	0	140.95	30.9	0	27.5	8.33
CCl4	0	0	292.82	32.5	0	65.1	7.15
Rb+ [BCl4]-	0	1	353.19	31.9	25	86.7	2.35
TeCl4	0	0	201.68	30.5	0	42.8	6.57
PCl4	0	0	223.33	30.4	0	48.2	6.13
Rb+ [ICl4]-	0	1	143.08	30.7	25	34.5	7.72
Rb+ [AsCl4]-	0	1	238.93	30.7	25	58.4	5.34
Rb+ [SbCl4]-	0	1	253.08	30.4	25	62.0	4.89
SiCl4	0	0	354.3	31.6	0	80.7	2.53
GeCl4	0	0	312.42	31.2	0	70.3	4.18
SnCl4	0	0	306.03	30.2	0	69.0	4.04
Rb+ [AlCl4]-	0	1	361.41	30.6	25	89.1	1.25
Rb+ [GaCl4]-	0	1	308.59	31.1	25	75.7	2.95
Rb+ [InCl4]-	0	1	303.63	29.6	25	74.9	2.71
XeCl4	0	0	0.78	30.3	0	-7.4	9.53
PCl5	0	0	262.89	41.0	0	44.4	6.47
AsCl5	0	0	261.81	40.8	0	44.2	6.46
SbCl5	0	0	292.66	39.5	0	50.6	5.60
Rb+ [SeCl5]-	0	1	212.78	40.0	25	39.6	7.41
Rb+ [GeCl5]-	0	1	336.67	39.8	25	64.5	4.34
Rb+ [SnCl5]-	0	1	352.62	39.0	25	67.8	3.91
Rb+ [TeCl5]-	0	1	249.43	39.8	25	47.0	6.47
Rb+ [PCl6]-	0	1	298.47	50.7	25	45.5	6.83
Rb+ [AsCl6]-	0	1	306.35	52.0	25	46.6	6.39

Table S2 continued

Rb+ [SbCl6]-	0	1	353.66	49.1	25	55.0	5.44
Rb+ Rb[GeCl6]-	1	1	445.66	56.8	5	65.7	4.29
Rb+ Rb[SnCl6]-	1	1	475.93	56.0	5	70.9	3.81
Rb+ Rb[SeCl6]-	1	1	337.97	55.1	5	48.0	7.34
Rb+ Rb[TeCl6]-	1	1	374.59	53.9	5	54.3	6.36
Rb+ Rb2[InCl6]-	2	1	545.28	62.9	-15	77.9	2.53

Best fit parabolic curve:

$\gamma = 9.04 - 0.004542(\Delta G) - 0.0009708(\Delta G)^2$ RMS: 0.487

Data for individual chlorides

5.1 SCl_2
Geometry: bent, Cl-S-Cl 103.6° S-Cl 2.044 Å
Thermodynamics: H -1318.387626 Hartree S 67.282 cal mol^{-1} K^{-1}
Charges: S +0.532 Cl -0.176
(well known)

5.2 OCl_2
Geometry: bent, Cl-O-Cl 112.5° O-Cl 1.711 Å
Thermodynamics: H -995.351798 Hartree S 63.868 cal mol^{-1} K^{-1}
Charges: O -0.459 Cl +0.230
(well known)

5.3 $SeCl_2$
Geometry: bent, Cl-Se-Cl 101.1° Se-Cl 2.178 Å
Thermodynamics: H -3321.413782 Hartree S 70.486 cal mol^{-1} K^{-1}
Charges: Se +0.580 Cl -0.290
Reference: A. Maaninen *et al., Inorg. Chem.,* 1999, **38**, 4093-4097.

5.4 $TeCl_2$
Geometry: bent, Cl-Te-Cl 98.8° Te-Cl 2.366 Å
Thermodynamics: H -7534.091207 Hartree S 73.053 cal mol^{-1} K^{-1}
Charges: Te +0.822 Cl -0.411
Reference: G. H. Westphal *et al., J. Chem. Phys.,* 1980, **72**, 5192-5201.

5.5 $[BrCl_2]^-$
Geometry: linear, Br-Cl 2.449 Å
Thermodynamics: H -3494.098300 Hartree S 68.158 cal mol^{-1} K^{-1}
Charges: Br +0.108 Cl -0.554
(well known)

5.6 $[ICl_2]^-$
Geometry: linear, I-Cl 2.616 Å
Thermodynamics: H -7840.419273 Hartree S 69.850 cal mol^{-1} K^{-1}
Charges: I +0.263 Cl -0.631
(well known)

5.7 $XeCl_2$
Geometry: linear, Xe-Cl 2.511 Å
Thermodynamics: H -8154.350690 Hartree S 68.682 cal mol^{-1} K^{-1}
Charges: Xe +0.763 Cl-0.382
Reference: I. R. Beattie *et al. J. Chem. Soc., Dalton Trans.,* 1975*,* 1659-1662.

5.8 $[Cl_3]^-$
Geometry: linear, Cl-Cl 2.357 Å
Thermodynamics: H -1380.492632 Hartree S 67.574 cal mol^{-1} K^{-1}

Charges:	QTAIM	NBO	Hirshfeld	Mulliken
Cl(central)	-0.019	-0.053	-0.110	-0.039
Cl(terminal)	-0.490	-0.474	-0.445	-0.481

(well known)

5.9 PCl_3
Geometry: trigonal pyramidal, Cl-P-Cl 100.8° P-Cl 2.068 Å
Thermodynamics: H -1721.778946 Hartree S 76.636 cal mol^{-1} K^{-1}
Charges: P +1.252 Cl -0.418
(well known)

5.10 NCl_3
Geometry: trigonal pyramidal, Cl-N-Cl 108.4° N-Cl 1.766 Å
Thermodynamics: H -1435.024649 Hartree S 72.788 cal mol^{-1} K^{-1}
Charges: N -0.238 Cl +0.080
(well known)

5.11 BCl_3
Geometry: trigonal planar, B-Cl 1.748 Å
Thermodynamics: H -1405.388801 Hartree S 72.633 cal mol^{-1} K^{-1}
Charges: B +1.904 Cl -0.635
(well known)

5.12 $AsCl_3$
Geometry: trigonal pyramidal, Cl-As-Cl 99.2° As-Cl 2.193 Å
Thermodynamics: H -3615.982977 Hartree S 80.208 cal mol^{-1} K^{-1}
Charges: As +1.280 Cl -0.427
(well known)

5.13 $SbCl_3$
Geometry: trigonal pyramidal, Cl-Sb-Cl 97.0° Sb-Cl 2.375 Å
Thermodynamics: H -7695.980802 Hartree S 83.342 cal mol^{-1} K^{-1}
Charges: Sb +1.507 Cl -0.503
(well known)

5.14 $GaCl_3$
Geometry: trigonal planar, Ga-Cl 2.119 Å
Thermodynamics: H -3305.062817 Hartree S 81.124 cal mol^{-1} K^{-1}
Charges: Ga +1.460 Cl -0.487
Reference: O. Grabandt *et al.*, *Chem. Phys.,* 1990, **143**, 227-238.

5.15 $InCl_3$
Geometry: trigonal planar, In-Cl 2.334 Å
Thermodynamics: H -7122.668082 Hartree S 85.003 cal mol^{-1} K^{-1}
Charges: In +1.596 Cl -0.532
Reference: A. Givan and A. Loewenschuss, *J. Mol. Struct.,* 1979, **55**, 163-168.

5.16 $AlCl_3$
Geometry: trigonal planar, Al-Cl 2.079 Å
Thermodynamics: H -1623.021140 Hartree S 78.447 cal mol^{-1} K^{-1}
Charges: Al +2.333 Cl -0.778
(well known)

5.17 SCl_4
Geometry: seesaw, Cl-S-Cl (ax-eq) 93.0º, (eq-eq) 104.1º
S-Cl(ax) 2.291 Å, S-Cl(eq) 2.035 Å
Thermodynamics: H -2238.599606 Hartree S 86.786 cal mol^{-1} K^{-1}

Charges:	QTAIM	NBO	Hirshfeld	Mulliken
S	+0.833	+0.772	+0.426	+0.476
Cl(ax)	-0.369	-0.359	-0.256	-0.297
Cl(eq)	-0.048	-0.027	+0.043	+0.059

Reference: R. Steudel *et al., Z Naturforschung B,* 1987, **42***,* 163-168.

5.18 CCl_4
Geometry: tetrahedral, C-Cl 1.780 Å
Thermodynamics: H -1878.623924 Hartree S 78.582 cal mol^{-1} K^{-1}
Charges: C +0.425 Cl -0.106
(well known)

5.19 $[BCl_4]^-$
Geometry: tetrahedral, B-Cl 1.873 Å
Thermodynamics: H -1865.675175 Hartree S 80.860 cal mol^{-1} K^{-1}
Charges: B +1.825 Cl -0.706
Reference: J. A. Creighton, *J. Chem. Soc.*, 1965, 6589-6591.

5.20 $TeCl_4$
Geometry: seesaw, Cl-Te-Cl (ax-eq) 88.5º, (eq-eq) 101.0º
Te-Cl(ax) 2.471 Å, Te-Cl(eq) 2.345 Å
Thermodynamics: H -8454.387937 Hartree S 92.267 cal mol^{-1} K^{-1}
Charges: Te +1.713 Cl(ax) -0.509 Cl(eq) -0.348
(well known)

5.21 PCl_4
Geometry: seesaw, Cl-P-Cl (ax-eq) 100.7º, (eq-eq) 103.7º
P-Cl(ax) 2.185 Å, P-Cl(eq) 2.037 Å
Thermodynamics: H -2181.884912 Hartree S 88.832 cal mol^{-1} K^{-1}
Charges: P +1.435 Cl(ax) -0.339 Cl(eq) -0.379
Reference: J. P. Michaut and J. Roncin, *New J. Chem.,* 1997, **21**, 547-556.

5.22 $[ICl_4]^-$
Geometry: square planar, I-Cl 2.558 Å
Thermodynamics: H -8760.679991 Hartree S 92.081 cal mol^{-1} K^{-1}

Charges:	QTAIM	NBO	Hirshfeld	Mulliken
I	+1.142	+1.103	+0.263	+0.708
Cl	-0.535	-0.526	-0.316	-0.427

Reference: J. Shamir *et al.*, *Inorg. Chim. Acta,* 1986, **114**, 35-39.

5.23 $[AsCl_4]^-$
Geometry: seesaw, Cl-As-Cl (ax-eq) 93.0º, (eq-eq) 98.4º
As-Cl(ax) 2.482 Å, As-Cl(eq) 2.235 Å
Thermodynamics: H -4076.250984 Hartree S 91.768 cal mol^{-1} K^{-1}
Charges: As +1.331 Cl(ax) -0.675 Cl(eq) -0.491
Reference: L. A. Lesikar and A. F. Richards, *J. Organomet. Chem.,* 2006, **691**, 4250–4256.

5.24 $[SbCl_4]^-$
Geometry: seesaw, Cl-Sb-Cl (ax-eq) 89.3º, (eq-eq) 98.1º
Sb-Cl(ax) 2.605 Å, Sb-Cl(eq) 2.423 Å
Thermodynamics: H -8156.262762 Hartree S 94.398 cal mol^{-1} K^{-1}
Charges: Sb +1.553 Cl(ax) -0.707 Cl(eq) -0.570
Reference: N. Weslati *et al.*, *Physica B*, 2014, **441**, 42-46.

5.25 $SiCl_4$
Geometry: tetrahedral, Si-Cl 2.033 Å
Thermodynamics: H -2130.205740 Hartree S 83.880 cal mol^{-1} K^{-1}
Charges: Si +2.734 Cl -0.684
(well known)

5.26 $GeCl_4$
Geometry: tetrahedral, Ge-Cl 2.131 Å
Thermodynamics: H -3917.348839 Hartree S 88.376 cal mol^{-1} K^{-1}
Charges: Ge +1.792 Cl -0.448
(well known)

5.27 $SnCl_4$
Geometry: tetrahedral, Sn-Cl 2.324 Å
Thermodynamics: H -7865.641325 Hartree S 92.851 cal mol^{-1} K^{-1}
Charges: Sn +1.980 Cl -0.495
(well known)

5.28 $[AlCl_4]^-$
Geometry: tetrahedral, Al-Cl 2.169 Å
Thermodynamics: H -2083.357909 Hartree S 87.721 cal mol^{-1} K^{-1}
Charges: Al +2.377 Cl -0.844
(well known)

5.29 $[GaCl_4]^-$
Geometry: tetrahedral, Ga-Cl 2.210 Å
Thermodynamics: H -3765.388331 Hartree S 88.873 cal mol^{-1} K^{-1}
Charges: Ga +1.522 Cl -0.631
Reference: T. Timofte and A.-V. Mudring, *Z. Anorg. Allg. Chem.*, 2009, **635**, 840-847.

5.30 $[InCl_4]^-$
Geometry: tetrahedral, In-Cl 2.413 Å
Thermodynamics: H -7583.004694 Hartree S 95.538 cal mol^{-1} K^{-1}
Charges: In +1.643 Cl -0.661
Reference: J. Trotter *et al.*, *Acta Cryst. B,* 1969, **25**, 603-604.

5.31 $XeCl_4$
Geometry: square planar, Xe-Cl 2.504 Å
Thermodynamics: H -9074.500299 Hartree S 90.821 cal mol^{-1} K^{-1}
Charges: Xe +1.237 Cl -0.309
Reference: G. J. Perlow and M. R. Perlow, *J. Chem. Phys.,* 1964, **41**, 1157-1158.

5.32 PCl_5
Geometry: trigonal bipyramidal, P-Cl(ax) 2.153 Å, P-Cl(eq) 2.051 Å
Thermodynamics: H -2642.029319 Hartree S 91.479 cal mol^{-1} K^{-1}
Charges: P +1.767 Cl(ax) -0.383 Cl(eq) -0.334
(well known)

5.33 $AsCl_5$
Geometry: trigonal bipyramidal, As-Cl(ax) 2.242 Å, As-Cl(eq) 2.170 Å
Thermodynamics: H -4536.224577 Hartree S 94.741 cal mol^{-1} K^{-1}
Charges: As +1.769 Cl(ax) -0.401 Cl(eq) -0.322
Reference: S. Haupt and K. Seppelt, *Z. Anorg. Allg. Chem*. 2002, **628**, 729-734.

5.34 $SbCl_5$
Geometry: trigonal bipyramidal, Sb-Cl(ax) 2.381 Å, Sb-Cl(eq) 2.335 Å
Thermodynamics: H -8616.262956 Hartree S 100.319 cal mol^{-1} K^{-1}
Charges: Sb +2.199 Cl(ax) -0.471 Cl(eq) -0.419
Reference: S. Haupt and K. Seppelt, *Z. Anorg. Allg. Chem*. 2002, **628**, 729-734.

5.35 $[SeCl_5]^-$
Geometry: square pyramidal, Se-Cl(ax) 2.200 Å, Se-Cl(eq) 2.406 Å
Thermodynamics: H -4701.958499 Hartree S 98.357 cal mol^{-1} K^{-1}
Charges: Se +1.293 Cl(ax) -0.264 Cl(eq) -0.507
Reference: R. J. Gillespie *et al.*, *Inorg. Chem.*, 1990, **29**, 1251-1259.

5.36 $[GeCl_5]^-$
Geometry: trigonal bipyramidal, Ge-Cl(ax) 2.310 Å, Ge-Cl(eq) 2.216 Å
Thermodynamics: H -4377.613072 Hartree S 98.578 cal mol^{-1} K^{-1}
Charges: Ge +1.829 Cl(ax) -0.609 Cl(eq) -0.537
Reference: C. J. Carmalt *et al.*, *Chem. Commun.*, 1997, 2095-2096.

5.37 $[SnCl_5]^-$
Geometry: trigonal bipyramidal, Sn-Cl(ax) 2.451 Å, Sn-Cl(eq) 2.399 Å
Thermodynamics: H -8325.941156 Hartree S 102.888 cal mol^{-1} K^{-1}
Charges: Sn +2.044 Cl(ax) -0.632 Cl(eq) -0.593
Reference: J. Shamir *et al.*, *Inorg. Chem.,* 1985, **24**, 2301-2309.

5.38 $[TeCl_5]^-$
Geometry: square pyramidal, Te-Cl(ax) 2.386 Å, Te-Cl(eq) 2.539 Å
Thermodynamics: H -8914.689619 Hartree S 100.525 cal mol^{-1} K^{-1}
Charges: Te +1.764 Cl(ax) -0.417 Cl(eq) -0.586
Reference: T. Schöenherr, *Z. Naturforsch. B,* 1988, **43**, 159-164.

5.39 $[PCl_6]^-$
Geometry: octahedral, P-Cl 2.182 Å
Thermodynamics: H -3102.311600 Hartree S 98.203 cal mol^{-1} K^{-1}
Charges: P +1.582 Cl -0.431
Reference: P. N. Gates *et al.*, *J. Chem. Soc., Dalton Trans.,* 1995, 2719-2722.

5.40 $[AsCl_6]^-$
Geometry: octahedral, As-Cl 2.279 Å
Thermodynamics: H -4996.521141 Hartree S 96.278 cal mol^{-1} K^{-1}
Charges: As +1.806 Cl -0.467
Reference: S. Haupt and K. Seppelt, *Z. Anorg. Allg. Chem*. 2002, **628**, 729-734.

5.41 $[SbCl_6]^-$
Geometry: octahedral, Sb-Cl 2.2422 Å
Thermodynamics: H -9076.585766 Hartree S 107.443 cal mol^{-1} K^{-1}
Charges: Sb +2.279 Cl -0.547
Reference: G. R. Willey *et al., J. Indian Chem. Soc.,* 1998*,* **75***,* 271-275*.*

5.42 $[RbGeCl_6]^-$
Cartesian coordinates:
```
Ge   0.102911   -1.775202    0.107731
Cl  -1.522223   -2.946760   -0.930620
Cl   1.748406   -0.288593    1.173036
Cl   1.805399   -2.906947   -0.847557
Cl  -1.631797   -0.327232    1.088199
Cl   0.070431   -3.018742    1.990164
Cl   0.130301   -0.214530   -1.794699
Rb   0.051789    2.169526    0.233045
```
Thermodynamics: H -7777.652359 Hartree S 120.324 cal mol^{-1} K^{-1}
Charges: Ge +1.854 Cl -0.688 (x 3), -0.571 (x 3) Rb +0.919
Reference: P. N. Bartlett *et al.*, *Chem. Eur. J.,* 2014, **20**, 5019-5027.

5.43 $[RbSnCl_6]^-$

Cartesian coordinates:

```
Sn   -0.813116   -0.572513   -0.130983
Cl   -2.542293    0.010572   -1.735625
Cl    1.155161   -0.959839    1.492283
Cl   -0.646284   -2.889709   -0.843005
Cl   -0.714836    1.896172    0.611668
Cl   -2.362859   -1.027857    1.684276
Cl    0.978370    0.060915   -1.875294
Rb    2.425330    1.708772    0.390671
```

Thermodynamics: H -11726.003263 Hartree S 124.427 cal mol^{-1} K^{-1}

Charges: Sn +2.094 Cl -0.713 (x 3), -0.625 (x 3) Rb +0.920

Reference: J. Shamir *et al.*, *Inorg. Chem.,* 1985, **24**, 2301-2309.

5.44 $[RbSeCl_6]^-$

Cartesian coordinates:

```
Se    0.789721   -0.536806   -0.266572
Cl    0.654902   -2.496327   -1.431143
Cl    2.463001    0.246318   -1.608576
Cl   -1.014284    0.539165   -1.955551
Cl   -1.216691   -1.388694    1.314089
Cl    2.282506   -1.420826    1.217308
Cl    0.863641    1.781628    1.103929
Rb   -2.252783    1.528302    0.758600
```

Thermodynamics: H -8102.023608 Hartree S 126.094 cal mol^{-1} K^{-1}

Charges: Se +1.317 Cl -0.703 (x 3), -0.374 (x 3) Rb +0.912

Reference: W. Abriel, *Acta Cryst. C*, 1986, **42**, 1113-1115.

5.45 $[RbTeCl_6]^-$

Cartesian coordinates:

```
Te    0.781143   -0.531709   -0.263895
Cl    0.691252   -2.649312   -1.519154
Cl    2.608367    0.265474   -1.709441
Cl   -1.111013    0.602849   -2.000176
Cl   -1.327742   -1.394515    1.379656
Cl    2.420873   -1.502833    1.298492
Cl    0.829578    1.885866    1.161716
Rb   -2.322444    1.576939    0.784885
```

Thermodynamics: H -12314.754681 Hartree S 131.577 cal mol^{-1} K^{-1}

Charges: Te +1.821 Cl -0.734 (x 3), -0.512 (x 3) Rb +0.916

Reference: W. Abriel, *Z. Naturforschung B*, 1986, **41**, 592-598.

5.46 $[Rb_2InCl_6]^-$

Cartesian coordinates:

```
In    -0.744431    0.491536    0.910288
Cl    -2.494240    0.161256    2.811485
Cl     1.005542    0.820999   -0.989585
Cl     0.210412    2.557820    2.167535
Cl    -1.701020   -1.574703   -0.346087
Cl    -2.417345    2.045773   -0.343190
Cl     0.926877   -1.063497    2.165223
Rb     1.339429   -2.289019   -0.697515
Rb    -2.830949    3.270442    2.520152
```

Thermodynamics: H -14383.120999 Hartree S 140.770 cal mol^{-1} K^{-1}

Charges: In +1.735 Cl -0.759 (x 6) Rb +0.911 (x 2)

Reference: H. Narita *et al.*, *Dalton Trans.,* 2014, **43**, 1630-1635.

6. Data for oxides XO_n^{m-}

Table S3. Data for Graph Shown in Fig. 3

species	explicit Rb	implicit Rb	ΔH kcal/mol	TΔS kcal/mol	ΔG (Rb.O) kcal/mol	ΔG (X-O) kcal/mol	γ(X)
O3	0	0	126.9	15.3	0.0	55.8	9.52
CO2	0	0	375.71	17.1	0.0	179.3	3.51
NO2	0	0	216.63	15.6	0.0	100.5	7.58
Rb+ [CO2]-	0	1	268.5	15.6	54.0	153.4	3.99
Rb+ [NO2]-	0	1	171.14	16.2	54.0	104.5	7.19
PO2	0	0	241.01	14.8	0.0	113.1	3.94
SO2	0	0	221.87	15.1	0.0	103.4	5.25
SeO2	0	0	182.71	14.9	0.0	83.9	6.38
TeO2	0	0	159.24	15.6	0.0	71.8	6.07
ClO2	0	0	90.77	14.3	0.0	38.2	8.31
BrO2	0	0	86.64	14.1	0.0	36.3	8.07
Rb+ [ClO2]-	0	1	51.08	15.2	54.0	44.9	8.58
Rb+ [BrO2]-	0	1	50.44	15.1	54.0	44.7	8.17
NO3	0	0	264.88	24.1	0.0	80.3	8.14
Rb+ [NO3]-	0	1	265.09	25.4	54.0	97.9	8.30
Rb+ Rb[CO3]-	1	1	420.13	32.6	45.1	144.2	3.70
SO3	0	0	288.12	24.7	0.0	87.8	4.85
Rb+ Rb[SO3]-	1	1	301.22	33.0	45.1	104.4	5.22
SeO3	0	0	222.14	24.3	0.0	66.0	6.95
Rb+ Rb[SeO3]-	1	1	271.54	32.6	45.1	94.7	6.30
Rb+ Rb[TeO3]-	1	1	267.12	32.3	45.1	93.3	5.90
Rb+ [ClO3]-	0	1	111.69	24.7	54.0	47.0	8.67
Rb+ [BrO3]-	0	1	113.28	24.4	54.0	47.6	8.14
Rb+ Rb2[AsO3]-	2	1	322.77	40.3	36.2	106.2	4.66
Rb+ [IO3]-	0	1	134.3	24.0	54.0	54.8	7.28
XeO3	0	0	-5.96	23.1	0.0	-9.7	9.00
XeO4	0	0	5.03	32.6	0.0	-6.9	9.72
Rb+ [ClO4]-	0	1	164.27	34.8	54.0	45.9	9.11
Rb+ [BrO4]-	0	1	159.1	34.2	54.0	44.7	8.71
Rb+ [IO4]-	0	1	189.88	33.6	54.0	52.6	7.54
Rb+ Rb[SO4]-	1	1	409.66	43.3	45.1	102.9	4.34
Rb+ Rb[SeO4]-	1	1	348.84	42.6	45.1	87.9	6.46
Rb+ Rb[TeO4]-	1	1	340.15	41.4	45.1	86.0	5.90
Rb+ Rb2[NO4]-	2	1	346.97	51.9	36.2	82.8	8.50
Rb+ Rb2[PO4]-	2	1	520.03	51.7	36.2	126.1	2.60
Rb+ Rb2[AsO4]-	2	1	438.49	50.9	36.2	106.0	4.83
Rb+ Rb3[SiO4]-	3	1	571.06	59.6	27.3	134.7	1.65
Rb+ Rb3[SeO5]-	3	1	434.3	68.3	27.3	78.7	6.70
Rb+ Rb[BrO5]- r	1	1	208.37	50.9	45.1	40.5	8.88
Rb+ Rb[IO5]- r	1	1	270.71	50.3	45.1	53.1	7.38

Table S3 continued

species	explicit Rb	implicit Rb	ΔH kcal/mol	TΔS kcal/mol	ΔG (Rb.O) kcal/mol	ΔG (X-O) kcal/mol	γ(X)
Rb+ Rb2[IO5]-	2	1	334.52	58.7	36.2	62.4	7.47
Rb+ Rb3[XeO6]-	3	1	277.55	78.9	27.3	37.7	9.41
Rb+ Rb4[IO6]-	4	1	509.05	88.1	18.4	73.2	7.26
Rb+ Rb5[TeO6]-	5	1	637.28	96.4	9.6	91.7	5.63
Rb+ Rb5[SeO6]-	5	1	569.12	95.8	9.6	80.5	6.81

Best fit parabolic curve:

$\gamma = 9.50 - 0.01432(\Delta G) - 0.0002947(\Delta G)^2$ RMS: 0.536

Data for individual oxides

6.1 O_3
Geometry: bent, O-O-O 117.7° O-O 1.252 Å
Thermodynamics: H -225.358505 Hartree S 58.177 cal mol^{-1} K^{-1}

Charges:	QTAIM	NBO	Hirshfeld	Mulliken
O(central)	+0.238	+0.324	+0.218	+0.287
O(terminal)	-0.119	-0.162	-0.109	-0.143

(well known)

6.2 CO_2
Geometry: linear, C-O 1.166 Å
Thermodynamics: H -188.534743 Hartree S 51.048 cal mol^{-1} K^{-1}
Charges: C +2.243 O -1.121
(well known)

6.3 NO_2
Geometry: bent, O-N-O 133.8° N-O 1.198 Å
Thermodynamics: H -205.022171 Hartree S 57.346 cal mol^{-1} K^{-1}
Charges: N +0.710 O -0.355
(well known)

6.4 $[CO_2]^-$
Geometry: bent, O-C-O 133.8° C-O 1.249 Å
Thermodynamics: H -188.508134 Hartree S 57.380 cal mol^{-1} K^{-1}
Charges: C +1.506 O -1.253
Reference: Z. H. Kafafi *et al., J. Am. Chem. Soc.*, 1983, **105**, 3886-3893.

6.5 $[NO_2]^-$
Geometry: bent, O-N-O 115.5° N-O 1.265 Å
Thermodynamics: H -205.093902 Hartree S 56.612 cal mol^{-1} K^{-1}
Charges: N +0.407 O -0.703
(well known)

6.6 PO_2
Geometry: bent, O-P-O 134.0° P-O 1.478 Å
Thermodynamics: H -491.691823 Hartree S 62.225 cal mol^{-1} K^{-1}

Charges:	QTAIM	NBO	Hirshfeld	Mulliken
P	+2.529	+1.822	+0.572	+0.943
O	-1.265	-0.911	-0.286	-0.472

Reference: H.-B. Qian *et al., J. Chem. Soc., Faraday Trans.,* 1995, **91**, 2993-2998.

6.7 SO_2
Geometry: bent, O-S-O 118.4° S-O 1.449 Å
Thermodynamics: H -548.507293 Hartree S 60.722 cal mol^{-1} K^{-1}

Charges:	QTAIM	NBO	Hirshfeld	Mulliken
S	+2.375	+1.598	+0.477	+0.940
O	-1.187	-0.799	-0.238	-0.470

(well known)

6.8 SeO_2
Geometry: bent, O-Se-O 114.2° Se-O 1.616 Å
Thermodynamics: H -2551.459509 Hartree S 64.141 cal mol^{-1} K^{-1}

Charges:	QTAIM	NBO	Hirshfeld	Mulliken
Se	+1.808	+1.716	+0.549	+0.876
O	-0.904	-0.858	-0.275	-0.438

Reference: R.J.M Konings *et al., Chem. Phys. Lett.,* 1998, **292**, 447–453.

6.9 TeO_2
Geometry: bent, O-Te-O 112.5° Te-O 1.817 Å
Thermodynamics: H -6764.099040 Hartree S 66.656 cal mol^{-1} K^{-1}

Charges:	QTAIM	NBO	Hirshfeld	Mulliken
Te	+1.964	+1.969	+0.757	+1.120
O	-0.982	-0.984	-0.379	-0.560

Reference: R.J.M Konings *et al., Chem. Phys. Lett.,* 1998, **292**, 447–453.

6.10 ClO_2
Geometry: bent, O-Cl-O 116.7° Cl-O 1.503 Å
Thermodynamics: H -610.330167 Hartree S 61.474 cal mol^{-1} K^{-1}

Charges:	QTAIM	NBO	Hirshfeld	Mulliken
Cl	+1.346	+1.293	+0.455	+0.974
O	-0.673	-0.646	-0.227	-0.487

(well known)

6.11 BrO_2
Geometry: bent, O-Br-O 114.3° Br-O 1.663 Å
Thermodynamics: H -2723.914782 Hartree S 66.009 cal mol^{-1} K^{-1}

Charges:	QTAIM	NBO	Hirshfeld	Mulliken
Br	+1.467	+1.474	+0.456	+0.869
O	-0.733	-0.737	-0.228	-0.435

(well known)

6.12 $[ClO_2]^-$
Geometry: bent, O-Cl-O 115.1° Cl-O 1.606 Å
Thermodynamics: H -610.411149 Hartree S 61.105 cal mol^{-1} K^{-1}
Charges: Cl +0.707 O -0.854
(well known)

6.13 $[BrO_2]^-$
Geometry: bent, O-Br-O 114.2° Br-O 1.750 Å
Thermodynamics: H -2724.001324 Hartree S 64.095 cal mol^{-1} K^{-1}
Charges: Br +0.915 O -0.957
Reference: W. Levason *et al., J. Chem. Soc., Dalton Trans.,* 1990, 349-353.

6.14 NO_3
Geometry: planar, Y-shaped, O-N-O 111.8°, 124.1° (x2) N-O 1.207 Å, 1.252 Å (x 2)
Thermodynamics: H -280.151142 Hartree S 65.248 cal mol^{-1} K^{-1}
Charges: N +0.930 O -0.368, -0.281 (x 2)
Reference: I. Hirota *et al., J. Chem. Phys.,* 1997, **107**, 2829-2838.

6.15 $[NO_3]^-$
Geometry: trigonal planar, N-O 1.260 Å
Thermodynamics: H -280.295711 Hartree S 62.248 cal mol^{-1} K^{-1}
Charges: N +0.849 O -0.616
(well known)

6.16 $[RbCO_3]^-$
Cartesian coordinates:

C	1.339367	-1.202150	0.122436
O	2.271309	-2.047184	0.208901
O	1.590929	0.099775	0.020126
O	0.062633	-1.574553	0.129714
Rb	-0.875465	0.806107	-0.081334

Thermodynamics: H -3203.586097 Hartree S 77.659 cal mol^{-1} K^{-1}
Charges: C +2.148 O -1.356 (x 2), -1.314 Rb +0.877
(well known)

6.17 SO_3
Geometry: trigonal planar, S-O 1.438
Thermodynamics: H -623.664941 Hartree S 65.016 cal mol^{-1} K^{-1}

Charges:	QTAIM	NBO	Hirshfeld	Mulliken
S	+3.576	+2.437	+0.654	+1.192
O	-1.192	-0.812	-0.218	-0.397

(well known)

6.18 $[RbSO_3]^-$
Cartesian coordinates:

S	0.379904	0.742842	0.045830
O	-0.173132	0.094525	-1.261315
O	-0.154569	-0.161248	1.199575
O	1.916764	0.476687	0.006677
Rb	1.098570	-2.148633	-0.255424

Thermodynamics: H -3563.614301 Hartree S 79.098 cal mol^{-1} K^{-1}
Charges: S +2.390 O -1.416 Rb +0.858
(well known)

6.19 SeO_3
Geometry: trigonal planar, Se-O 1.607 Å
Thermodynamics: H -2626.578625 Hartree S 69.192 cal mol^{-1} K^{-1}

Charges:	QTAIM	NBO	Hirshfeld	Mulliken
Se	+2.527	+2.542	+0.654	+1.416
O	-0.842	-0.847	-0.218	-0.472

(well known)

6.20 $[RbSO_3]^-$
Cartesian coordinates:

Se	-2.309061	0.626738	-0.111330
O	-1.826829	-0.517213	1.080833
O	-1.357530	2.006753	0.279182
O	-1.550427	0.033290	-1.537438
Rb	0.688830	0.137656	0.103761

Thermodynamics: H -5566.585822 Hartree S 83.062 cal mol^{-1} K^{-1}
Charges: Se +1.849 O -1.243 Rb +0.882
(well known)

6.21 $[RbTeO_3]^-$
Cartesian coordinates:

Te	-2.419944	0.645540	-0.119209
O	-1.813617	-0.604647	1.175251
O	-1.306119	2.122386	0.308962
O	-1.512851	-0.009926	-1.652806
Rb	0.697514	0.133871	0.102810

Thermodynamics: H -9779.251515 Hartree S 85.656 cal mol^{-1} K^{-1}
Charges: Te +2.052 O -1.315 Rb +0.893
Reference: L. Andersen *et al., Acta Cryst. B,* 1989*,* **45***,* 344-348*.*

6.22 $[ClO_3]^-$
Geometry: trigonal pyramidal, O-Cl-O 108.2º Cl-O 1.522 Å
Thermodynamics: H -685.559810 Hartree S 65.701 cal mol^{-1} K^{-1}
Charges: Cl +1.664 O -0.888
(well known)

6.23 $[BrO_3]^-$
Geometry: trigonal pyramidal, O-Br-O 107.6º Br-O 1.679 Å
Thermodynamics: H -2799.153553 Hartree S 69.247 cal mol^{-1} K^{-1}
Charges: Br +1.929 O -0.976
Reference: W. Levason *et al., J. Chem. Soc., Dalton Trans.,* 1990, 349-353.

6.24 $[Rb_2AsO_3]^-$
Cartesian coordinates:

As	-0.934133	0.495542	0.915001
O	-1.370013	1.549933	-0.401988
O	-1.670301	-1.070723	0.712547
O	0.833371	0.172103	0.630375
Rb	0.994653	1.785241	-1.477570
Rb	0.509088	-2.448923	0.319531

Thermodynamics: H -8340.783901 Hartree S 98.427 cal mol^{-1} K^{-1}
Charges: As +1.671 O -1.331 (x 2), -1.438 (x 1) Rb +0.716 (x 2)
Reference: M. B. Hamida and M. S. Wickleder, *Z. Anorg. Allg. Chem.,* 2006, **632**, 2195–2197.

6.25 $[IO_3]^-$
Geometry: trigonal pyramidal, O-I-O 106.8º I-O 1.841 Å
Thermodynamics: H -7145.496815 Hartree S 71.978 cal mol^{-1} K^{-1}
Charges: I +2.359 O -1.120
(well known)

6.26 XeO_3
Geometry: trigonal pyramidal, O-Xe-O 107.8º Xe-O 1.819 Å
Thermodynamics: H -7459.320352 Hartree S 72.436 cal mol^{-1} K^{-1}

Charges:	QTAIM	NBO	Hirshfeld	Mulliken
Xe	+2.498	+2.728	+0.746	+1.522
O	-0.833	-0.909	-0.249	-0.507

Reference: D. S. Brock and G. J. Schrobilgen, *J. Am. Chem. Soc.,* 2011*,* **133***,* 6265–6269.

6.27 XeO_4
Geometry: tetrahedral, Xe-O 1.801 Å
Thermodynamics: H -7534.389949 Hartree S 77.001 cal mol^{-1} K^{-1}

Charges:	QTAIM	NBO	Hirshfeld	Mulliken
Xe	+3.140	+3.512	+0.789	+1.754
O	-0.785	-0.878	-0.197	-0.439

Reference: H. Selig *et al., Science,* 1964*,* **143***,* 1322-1322.

6.28 $[ClO_4]^-$
Geometry: tetrahedral, Cl-O 1.484 Å
Thermodynamics: H -760.695680 Hartree S 68.299 cal mol^{-1} K^{-1}
Charges: Cl +2.446 O -0.861
(well known)

6.29 $[BrO_4]^-$
Geometry: tetrahedral, Br-O 1.648 Å
Thermodynamics: H -2874.278643 Hartree S 72.760 cal mol^{-1} K^{-1}
Charges: Br +2.646 O -0.912
Reference: W. Levason *et al., J. Chem. Soc., Dalton Trans.,* 1990, 349-353.

6.30 $[IO_4]^-$
Geometry: tetrahedral, I-O 1.819 Å
Thermodynamics: H -7220.637457 Hartree S 76.370 cal mol^{-1} K^{-1}
Charges: I +3.230 O -1.058
(well known)

6.31 $[RbSO_4]^-$, $[SO_4]^{2-}$
$[RbSO_4]^-$ Cartesian coordinates:

```
S     -1.275903   -0.610173   -0.493839
O     -0.088263   -0.170741   -1.341692
O     -0.956700   -0.200539    0.938907
O     -1.549699   -2.055363   -0.616982
O     -2.458462    0.244202   -0.932991
Rb    -0.712021    2.312496   -0.240882
```

Charges: S +3.832 O -1.449 (x3), -1.415 (x1) Rb +0.930
Thermodynamics: H -3638.839191 Hartree S 81.163 cal mol^{-1} K^{-1}

$[SO_4]^{2-}$ geometry: tetrahedral, S-O 1.514 Å

Charges:	QTAIM	NBO	Hirshfeld	Mulliken
S	+3.846	+2.613	+0.238	+1.305
O	-1.462	-1.153	-0.559	-0.826

(well known)

6.32 $[RbSeO_4]^-$
Cartesian coordinates:

```
Se    -1.280641   -0.639728   -0.496297
O      0.030226   -0.119793   -1.423976
O     -0.922697   -0.151954    1.079447
O     -1.587685   -2.248653   -0.633071
O     -2.570310    0.338094   -0.975141
Rb    -0.709940    2.341917   -0.238441
```

Thermodynamics: H -5641.761086 Hartree S 86.208 cal mol^{-1} K^{-1}
Charges: Se +2.771 O -1.199 (x 3), -1.099 Rb +0.925
(well known)

6.33 $[RbTeO_4]^-$
Cartesian coordinates:

```
Te    -1.212760   -0.112277   -0.111150
O     -0.304539    0.431594   -1.655089
O     -0.288442   -1.665911    0.376980
O     -3.035044   -0.287174   -0.277243
O     -0.628665    1.126801    1.164755
Rb     1.929606    0.176691    0.177889
```

Thermodynamics: H -9854.419971 Hartree S 91.555 cal mol^{-1} K^{-1}
Charges: Te +3.048 O -1.270 (x 3), -1.162 Rb +0.924
(well known)

6.34 $[Rb_2NO_4]^-$

Cartesian coordinates:

```
N     -0.441642    0.923038    0.423435
O     -1.705942    1.003932    0.904138
O     -0.463193    0.423184   -0.946351
O      0.199555    2.115911    0.468370
O      0.312217   -0.073322    1.177423
Rb    -1.776014   -1.532509    0.337743
Rb     2.169461    0.769891   -0.564616
```

Thermodynamics: H -6235.046760 Hartree S 90.994 cal mol^{-1} K^{-1}

Charges: N +0.749 O -0.934 (x 2), -0.808 (x 2) Rb +0.868 (x 2)

Reference: T. Bremm and M. Jansen, *Z. Anorg. Allg. Chem.,* 1992, **608**, 49-55.

6.35 $[Rb_2PO_4]^-$

Cartesian coordinates:

```
P      1.123869   -0.843343   -0.208509
O      0.676838   -0.060835    1.125235
O      2.480135   -0.277597   -0.701454
O      1.048633   -2.370364    0.046575
O     -0.033838   -0.421686   -1.244342
Rb     1.101558    2.052422   -0.642805
Rb    -1.501163   -1.752595    0.717248
```

Thermodynamics: H -6521.953349 Hartree S 93.884 cal mol^{-1} K^{-1}

Charges: P +3.701 O -1.637 (x 2), -1.615 (x 2) Rb +0.900 (x 2)

(well known)

6.36 $[Rb_2AsO_4]^-$

Cartesian coordinates:

```
As   -0.000293    0.052724    0.065908
O     1.396568   -0.734990   -0.501031
O     0.340453    1.772395    0.215333
O    -0.224171   -0.344350    1.765296
O    -1.484080   -0.143270   -0.742238
Rb    2.422483    0.608149    1.705345
Rb   -2.240417    1.564405    1.316013
```

Thermodynamics: H -8416.020390 Hartree S 99.222 cal mol^{-1} K^{-1}

Charges: As +2.585 O -1.388 (x 2), -1.301 (x 2) Rb +0.896 (x 2)

(well known)

6.37 $[Rb_3SiO_4]^-$

Cartesian coordinates:

```
Si    -0.842292    1.193294   -0.606321
O     -1.921495    1.396995   -1.846950
O     -1.534194    0.940746    0.877904
O     -0.081799   -0.345690   -0.973272
O      0.423963    2.261643   -0.606996
Rb    -2.785356   -0.946580   -0.688960
Rb     1.139954    0.497019    1.382788
Rb     0.487906    1.265782   -3.173844
```

Thermodynamics: H -9409.931020 Hartree S 107.095 cal mol^{-1} K^{-1}

Charges: Si +3.173 O -1.693 (x 3), -1.695 (x 1) Rb +0.867 (x 3)

Reference: W. Klein and M. Jansen, *Z. Anorg. Allg. Chem.,* 2008, **634**, 1077-1081.

6.38 $[Rb_3SeO_5]^-$

Cartesian coordinates:

Se	0.467555	-0.749540	-0.602275
O	-1.157189	-0.856432	-1.330023
O	1.894916	-1.676978	-0.299436
O	0.410064	0.880245	0.210946
O	1.150343	-0.153896	-2.112696
O	-0.224655	-1.523464	0.994187
Rb	1.979605	-0.549285	2.101883
Rb	-0.504862	1.739695	-2.303276
Rb	-2.316604	-0.114265	0.947917

Thermodynamics: H -11596.517853 Hartree S 117.491 cal mol^{-1} K^{-1}

Charges: Se +2.652 O -1.155, -1.239, -1.233, -1.292, -1.313 Rb +0.836, +0.863, +0.881

Reference: H. Haas and M. Jansen, *Z. Anorg. Allg. Chem.*, 2001, **627**, 755-760.

6.39 $[RbBrO_5]^-$

Cartesian coordinates:

Br	0.000000	0.000000	0.000000
O	0.000000	0.000000	1.686573
O	1.704382	0.000000	-0.516432
O	0.432439	-1.720450	-0.157064
O	-0.081012	1.766057	-0.214714
O	-1.496456	-0.325049	-0.706746
Rb	2.004096	1.962642	1.488445

Thermodynamics: H -5889.193480 Hartree S 93.756 cal mol^{-1} K^{-1}

Charges: Br +2.561 O -0.989 (x 2), -0.586, -0.856, -1.070 Rb +0.928

Reference: J.R. Byberg, *Inorg. Chem.*, 1993, **32**, 5513-5516.

6.40 $[RbIO_5]^-$

Cartesian coordinates:

I	-0.126863	0.188430	-0.015287
O	-0.130532	1.940121	0.510651
O	1.323519	0.168465	-1.176988
O	0.497095	-0.545471	1.599095
O	-1.788072	0.082135	-0.770273
O	-0.130230	-1.949590	-0.213075
Rb	2.580949	-1.850731	0.240164

Thermodynamics: H -10235.602601 Hartree S 97.132 cal mol^{-1} K^{-1}

Charges: I +3.309 O -1.110, -1.178, -0.767, -1.057, -1.128 Rb +0.931

Reference: J. R. Byberg, *J. Phys. Chem.,* 1992, **96**, 4220-4225.

6.41 $[Rb_2IO_5]^-$

Cartesian coordinates:

I	-1.536637	-0.390268	0.411174
O	-1.541506	1.370120	1.123931
O	-2.792361	-0.136739	-1.009656
O	0.059313	0.010023	-0.537634
O	-2.752130	-0.867351	1.790472
O	-1.147381	-2.228280	0.133370
Rb	-0.879577	-1.621983	-2.520250
Rb	-4.271241	1.261070	0.991900

Thermodynamics: H -13175.488533 Hartree S 109.851 cal mol^{-1} K^{-1}

Charges: I +3.263 O -1.201 (x 4), -1.286 Rb +0.914 (x 2)

Reference: H. Dölling and M. Trömel, *Naturwissenschaften*, 1973, **60**, 153-154.

6.42 $[Rb_3XeO_6]^-$
Cartesian coordinates:

Xe	0.029281	0.637923	0.200301
O	-0.535696	-0.034170	-1.580556
O	0.592645	1.142750	1.945250
O	-1.063280	-0.818678	0.994163
O	1.125028	1.934208	-0.658165
O	-1.494370	1.775591	0.234380
O	1.529795	-0.660307	0.113233
Rb	0.979917	-1.427219	2.625041
Rb	-3.062709	-0.199675	-0.689264
Rb	2.011807	0.106367	-2.413168

Thermodynamics: H -16504.425371 Hartree S 117.860 cal mol^{-1} K^{-1}
Charges: Xe +3.297 O -1.210 (x 3), -1.123 (x 3) Rb +0.901 (x 3)
Reference: M. A. M. Forgeron *et al., Inorg. Chem.,* 2007*,* **46**, 3585−3592.

6.43 $[Rb_4IO_6]^-$
Cartesian coordinates:

I	-0.030450	0.029246	-0.125637
O	0.056345	-1.896374	-0.536120
O	-0.119069	1.955592	0.283263
O	0.766853	0.439614	-1.880916
O	-0.827145	-0.381018	1.630013
O	1.768737	-0.058323	0.672727
O	-1.831178	0.117620	-0.923825
Rb	2.542351	-1.506373	-1.437259
Rb	0.839948	1.405811	2.710276
Rb	-2.438690	-2.119028	0.399996
Rb	-1.076482	2.349461	-2.179769

Thermodynamics: H -19130.387234 Hartree S 128.905 cal mol^{-1} K^{-1}
Charges: I +3.370 O -1.318 (x 4) Rb +0.884 (x 4)
Reference: A. Kovalevskiy and M. Jansen, *Z. Anorg. Allg. Chem.*, 2006, **632**, 577-581.

6.44 $[Rb_5TeO_6]^-$
Cartesian coordinates:

Te	-0.012891	0.420220	0.036073
O	1.261700	-0.723370	-1.132087
O	1.488011	1.542767	0.677244
O	-0.347851	1.676680	-1.459052
O	-1.512998	-0.853544	-0.617803
O	0.328913	-0.986254	1.521681
O	-1.281551	1.412798	1.188582
Rb	2.991729	-0.497641	1.003407
Rb	-0.570363	-0.240844	-3.135685
Rb	-0.110802	3.644562	0.322192
Rb	-2.375908	-0.750517	1.998941
Rb	0.089421	-2.955627	-0.262188

Thermodynamics: H -21764.139773 Hartree S 141.285 cal mol^{-1} K^{-1}
Charges: Te +3.193 O --1.423 (x 3), -1.390 (x 3) Rb +0.853 (x 3), +0.812, +0.875
Reference: A. Kovalevskiy and M. Jansen, *Z. Anorg. Allg. Chem.*, 2006, **632**, 577-581.

6.45 [Rb_5SeO_6]$^-$

Cartesian coordinates:

Se	-0.005682	0.013964	-0.004467
O	1.433462	-1.141577	-0.719810
O	1.196464	0.957014	0.986022
O	0.175336	1.163019	-1.406233
O	-1.258844	-1.117659	-1.045963
O	-0.193606	-1.330549	1.441230
O	-1.393094	0.981987	0.670676
Rb	2.467939	-1.068540	1.773017
Rb	0.362393	-0.648824	-3.144846
Rb	-0.007078	3.160131	0.274319
Rb	-2.853020	-1.015962	1.124326
Rb	-0.005589	-3.315944	-0.291330

Thermodynamics: H -17551.353264 Hartree S 143.001 cal mol^{-1} K^{-1}

Charges: Se +2.596 O -1.309 (x 3), -1.271 (x 3) Rb +0.835 (x 3), +0.770, +0.869

Reference: J. Haas, *Z. Anorg. Allg. Chem.*, 2001, **627**, 1313-1318.

7. Thermodynamic data for atoms and ions

Table S4. Enthalpies and entropies of free atoms and ions in their ground states, calculated at the wB97XD/DGDZVP level of theory

species	multiplicity	H, Hartrees	S, cal mol^{-1} K^{-1}
H	m=2	-0.498492	27.392
Li	m=2	-7.484814	33.176
B	m=2	-24.642674	34.519
C	m=3	-37.831855	35.581
N	m=4	-54.572791	36.613
O	m=3	-75.052079	36.438
F	m=2	-99.718538	36.145
Al	m=2	-242.312300	37.191
Si	m=3	-289.315698	38.104
P	m=4	-341.203585	38.979
S	m=3	-398.049560	38.502
Cl	m=2	-460.081358	37.964
Ar	m=1	-527.464859	36.984
Ga	m=2	-1924.426902	39.986
Ge	m=3	-2076.525529	41.001
As	m=4	-2235.400567	41.612
Se	m=3	-2401.068386	41.233
Br	m=2	-2573.672556	40.390
Kr	m=1	-2753.305863	39.195
Rb	m=2	-2939.784246	40.608
Rb^+	m=1	-2939.640014	39.231
In	m=2	-5742.051166	41.510
Sn	m=3	-6024.828197	42.392
Sb	m=4	-6315.389789	43.039
Te	m=3	-6613.741112	42.681
I	m=2	-6919.982319	41.806
Xe	m=1	-7234.173618	40.543

8. Data for species in Table 3

8.1 CLi_6
Geometry: octahedral, C-Li 2.054 Å
Charges: C -4.593 Li +0.766 (x 3), +0.767 (x 3)
Reference: H. Kudo, *Nature*, 1992, **355**, 432-434.

8.2 N_3^-
Geometry: linear, N-N 1.189 Å
Charges: N(terminal) -0.332 N(central) -0.335
(well known)

8.3 CH_2NN
Cartesian coordinates and *charges (QTAIM, NBO, Hirshfeld, Mulliken)*:

C	-1.016033	-0.654820	0.002537	*+0.270*	*-0.434*	*-0.129*	*-0.547*
N	0.073966	0.047431	-0.000320	*-0.672*	*+0.035*	*+0.112*	*+0.217*
N	1.033932	0.666074	-0.003033	*+0.147*	*-0.043*	*-0.098*	*-0.181*
H	-1.607035	-0.669364	-0.900478	*+0.127*	*+0.221*	*+0.058*	*+0.255*
H	-1.270808	-1.183830	0.908168	*+0.127*	*+0.221*	*+0.058*	*+0.255*

(well known)

8.4 CH_2NCH
Cartesian coordinates and *charges*:

H	-0.303247	-0.407256	0.224811	*+0.127*
C	0.160561	0.420734	0.770154	*+0.586*
N	1.200348	0.160501	1.364449	*-1.376*
C	2.283363	0.037019	2.026802	*+0.460*
H	3.232519	0.213834	1.533927	*+0.102*
H	2.239167	-0.242461	3.073175	*+0.102*

Reference: G. Maier *et al.*, *Chem. Ber.*, 1993, **126**, 2337-2352.

8.5 NNS
Geometry: linear, N-N 1.127 Å N-S 1.588 Å
Charges: N(terminal) +0.223 N(central) -0.627 S +0.404
Reference: R.D. Brown *et al.*, *J. Mol. Spec.*, 1990, **140**, 390-400.

8.6 PNS
Geometry: linear, P-N 1.511 Å N-S 1.572 Å
Charges: P +1.115 N -1.585 S +0.470
Reference: X. Zeng *et al.*, *Angew. Chem. Int. Ed.*, 2012, **51**, 3334-3339.

8.7 NS_2^-
Geometry: bent, S-N-S 122.1° N-S 1.645 Å
Charges: N -1.108 S +0.054
Reference: P. Hassanzadeh and L. Andrews, *J. Am. Chem. Soc.,* 1992, **114**, 83-91.

8.8 OLi_4
Geometry: tetrahedral, O-Li 1.790 Å
Charges: O -2.799 Li +0.700
Reference: J. Viallon *et al.*, *Eur. Phys. J.*, 2005, **D33**, 405-411.

8.9 XeF_2O_3
Geometry: trigonal bipyramidal (F axial, O equatorial), Xe-F 1.963 Å Xe-O 1.791 Å
Charges: Xe +3.340 F -0.553 O -0.745
Reference: J. L. Huston, *Inorg. Chem.,* 1982, **21**, 685-688.

8.10 NNO
Geometry: linear, N-N 1.130 Å N-O 1.193 Å
Charges: N(terminal) +0.258 N(central) +0.102 O -0.360
(well known)

8.11 PNO
Geometry: linear, 1.512 Å N-O 1.192 Å
Charges: P +1.024 N -0.622 O -0.401
Reference: T. Okabayashi *et al., J. Chem. Phys*., 1999, **111**, 3012-3017

8.12 HCNO
Geometry: linear, H-C 1.066 Å C-N 1.606 Å N-O 1.209 Å
Charges: H +0.282 C +0.727 N -0.579 O -0.430
(well known)

8.13 NH_4 radical
Geometry: tetrahedral, N-H 1.081 Å
Charges: N -1.032 H +0.258
Reference: G. I. Gellene and R. F. Porter, *J. Phys. Chem.,* 1984, **88**, 6680-6684.

8.14 N_5^+
Cartesian coordinates and *charges*:

N1	0.120240	0.000000	0.120553	*+0.570*
N2	0.094182	0.000000	1.234031	*-0.168*
N3	-0.235970	0.000000	2.502140	*+0.197*
N4	0.836178	0.000000	3.255397	*-0.168*
N5	1.576540	0.000000	4.087565	*+0.570*

Reference: K. O. Christe *et al., Angew. Chem. Int. Ed.,* 1999, **38**, 2004-2009.

8.15 FLi_2
Geometry: bent, Li-F-Li 100.2º F-Li 1.741 Å
Charges: F -1.418 Li +0.709
Reference: K. Yokoyama *et al., Chem. Phys. Lett.,* 2000, **330**, 339–346.

8.16 PPS
Geometry: linear, P-P 1.885 Å P-S 1.906 Å
Charges: P(terminal) +0.442 P(central) +0.086 S -0.529
Reference: Z.Mielke *et al.*, *J. Phys. Chem*., 1991, **95**, 75-79.

8.17 HArF
Geometry: linear, H-Ar 1.372 Å Ar-F 2.010 Å
Charges: H +0.255 Ar +0.429 F -0.684
Reference: L. Khriachtchev *et al., Nature,* 2000, **406**, 874-876.

8.18 HKrF
Geometry: linear, H-Kr 1.509 Å Kr-F 2.053 Å
Charges: H +0.131 Kr +0.578 F -0.709
Reference: M. Pettersson *et al.*, *J. Chem. Phys*., 2002, **116**, 2508-2515

8.19 Me_3NO
Cartesian coordinates and *charges*:

N1	-0.005337	-0.007411	-0.008111	*-0.470*
O2	-0.022938	1.354532	-0.034906	*-0.720*
C3	1.407094	-0.485662	0.015410	*+0.204*
C4	-0.713810	-0.489667	1.212456	*+0.204*
C5	-0.689972	-0.536973	-1.222649	*+0.204*
H6	1.462636	-1.578079	0.037445	*+0.033*
H7	1.893057	-0.090220	-0.874533	*+0.080*
H8	1.875607	-0.054696	0.898173	*+0.080*
H9	-0.718627	-1.582161	1.270531	*+0.033*
H10	-0.201968	-0.056979	2.069959	*+0.080*
H11	-1.728549	-0.098701	1.168477	*+0.079*
H12	-0.697623	-1.630903	-1.236444	*+0.033*
H13	-1.703902	-0.141507	-1.215529	*+0.080*
H14	-0.159356	-0.141524	-2.086709	*+0.080*

8.20 $ClNO_2$

Cartesian coordinates and *charges*:

Cl	-0.046718	-0.055329	0.054328	*+0.020*
N	0.278603	0.450619	1.798216	*+0.703*
O	1.284985	0.001375	2.260758	*-0.361*
O	-0.555337	1.168641	2.265316	*-0.361*

(well known)

8.21 HXeF

Geometry: linear, H-Xe 1.697 Å Xe-F 2.140 Å

Charges: H -0.062 Xe +0.805 F -0.743

Reference:

8.22 CF_3NO_2

Cartesian coordinates and *charges*:

C1	0.007984	-0.558715	0.000733	*+2.177*
N2	0.010140	0.989626	-0.002738	*+0.529*
O3	-1.093284	1.490880	0.069346	*-0.414*
O4	1.087702	1.540206	-0.075167	*-0.408*
F5	1.241167	-0.998097	-0.078246	*-0.629*
F6	-0.696957	-0.961826	-1.038469	*-0.627*
F7	-0.556752	-0.956918	1.124384	*-0.627*

(well known)

8.23 Ph_3I

Cartesian coordinates and *charges*:

I1	-0.908526	-0.230134	0.096104	*+0.849*
C2	-1.262516	2.012839	0.232238	*-0.301*
C3	-2.190743	2.447617	1.177690	*-0.080*
C4	-2.556872	3.794258	1.252932	*-0.067*
C5	-1.995449	4.716346	0.372622	*-0.064*
C6	-1.069978	4.288258	-0.580745	*-0.067*
C7	-0.706965	2.943509	-0.649090	*-0.065*
C8	-0.156695	-2.369691	-0.081972	*-0.301*
C9	0.938629	-2.871210	0.625331	*-0.065*
C10	1.263353	-4.225910	0.561044	*-0.067*
C11	0.492884	-5.094867	-0.213526	*-0.064*
C12	-0.603119	-4.603636	-0.919116	*-0.067*
C13	-0.924741	-3.245422	-0.848594	*-0.081*
C14	1.161646	0.307849	-0.122451	*-0.324*
C15	1.738662	1.174979	0.797915	*-0.019*
C16	3.082706	1.516790	0.654254	*-0.044*
C17	3.828783	1.000987	-0.404020	*-0.054*
C18	3.232944	0.136444	-1.320628	*-0.044*
C19	1.892162	-0.219493	-1.180844	*-0.019*
H20	-2.638687	1.738049	1.872842	*+0.047*
H21	-3.275238	4.121333	1.999120	*+0.057*
H22	-2.276376	5.763571	0.427498	*+0.057*
H23	-0.632084	5.003432	-1.271222	*+0.057*
H24	0.023418	2.627873	-1.391544	*+0.057*
H25	1.555438	-2.205364	1.225763	*+0.057*
H26	2.117013	-4.605339	1.115375	*+0.057*
H27	0.746956	-6.149073	-0.265560	*+0.057*
H28	-1.204892	-5.273886	-1.526123	*+0.057*
H29	-1.785201	-2.875981	-1.405817	*+0.047*
H30	1.151128	1.596123	1.607219	*+0.094*
H31	3.543410	2.186631	1.372939	*+0.072*
H32	4.873609	1.272517	-0.514322	*+0.069*
H33	3.808525	-0.264084	-2.148714	*+0.072*
H34	1.434861	-0.912171	-1.879846	*+0.094*

Reference: H. J. Reich *et al., J. Am. Chem. Soc.,* 1991, **113**, 1414-1416.

8.24 $MeONO_2$

Cartesian coordinates and *charges*:

C1	1.036047	-1.533684	0.034441	*+0.365*
O2	0.966012	-0.102365	0.093621	*-0.640*
N3	-0.324372	0.399584	-0.027437	*+0.872*
O4	-0.371949	1.601707	0.023084	*-0.407*
O5	-1.228175	-0.398485	-0.165718	*-0.443*
H6	2.099095	-1.735920	0.144235	*+0.088*
H7	0.473005	-1.984776	0.851512	*+0.082*
H8	0.672323	-1.901283	-0.925089	*+0.082*

(well known)

8.25 $MeNO_2$

Cartesian coordinates and *charges (QTAIM, NBO, Hirshfeld, Mulliken)*:

C1	-0.005244	-0.004064	0.057653	*+0.197*	*-0.439*	*-0.023*	*-0.527*
N2	0.008593	0.021481	1.546439	*+0.427*	*+0.550*	*+0.263*	*+0.385*
O3	1.104150	-0.001099	2.089668	*-0.468*	*-0.400*	*-0.218*	*-0.316*
O4	-1.067480	0.086555	2.122875	*-0.465*	*-0.394*	*-0.215*	*-0.316*
H5	0.742813	-0.721081	-0.270080	*+0.103*	*+0.230*	*+0.068*	*+0.258*
H6	-1.005725	-0.265486	-0.273215	*+0.104*	*+0.227*	*+0.064*	*+0.258*
H7	0.267278	0.995987	-0.276309	*+0.102*	*+0.225*	*+0.061*	*+0.258*

(well known)

8.26 $PhNO_2$

Cartesian coordinates and *charges*:

C1	0.303602	-1.279778	0.062834	*-0.003*
C2	1.630607	-1.517236	-0.280971	*-0.029*
C3	2.463449	-0.456810	-0.636481	*-0.052*
C4	1.973872	0.848807	-0.650379	*-0.030*
C5	0.649287	1.102984	-0.309221	*-0.003*
C6	-0.162629	0.030097	0.041895	*+0.158*
N7	-1.565472	0.290246	0.404276	*+0.398*
O8	-2.262132	-0.669442	0.710130	*-0.468*
O9	-1.954339	1.451289	0.378849	*-0.468*
H10	-0.363527	-2.085570	0.342607	*+0.129*
H11	2.013225	-2.531918	-0.270792	*+0.080*
H12	3.497463	-0.648526	-0.903590	*+0.079*
H13	2.623167	1.672161	-0.927122	*+0.080*
H14	0.244765	2.107474	-0.312162	*+0.129*

(well known)

8.27 Ph_4Se

Cartesian coordinates and *charges*:

Se1	-0.501211	0.808437	0.144010	*+0.951*
C2	0.850565	1.215985	1.760426	*-0.245*
C3	1.128163	2.578526	1.879501	*-0.088*
C4	2.001074	3.061882	2.855759	*-0.070*
C5	2.612456	2.173515	3.738794	*-0.067*
C6	2.341923	0.808887	3.637662	*-0.074*
C7	1.466477	0.337998	2.657739	*-0.081*
C8	1.058892	0.666382	-1.001674	*-0.285*
C9	2.100330	-0.208338	-0.714653	*-0.021*
C10	3.202255	-0.257693	-1.566659	*-0.045*
C11	3.267259	0.578856	-2.679122	*-0.054*
C12	2.221683	1.460601	-2.949435	*-0.044*
C13	1.105760	1.498548	-2.116662	*-0.024*
C14	-0.915571	-0.901878	0.962366	*-0.285*
C15	-0.671869	-2.104122	0.308708	*-0.021*
C16	-1.002741	-3.301632	0.940448	*-0.045*
C17	-1.593262	-3.289554	2.202484	*-0.054*
C18	-1.843843	-2.076566	2.842530	*-0.044*
C19	-1.493645	-0.876336	2.228501	*-0.024*
C20	-1.727904	0.197785	-1.508889	*-0.245*
C21	-3.066299	0.547872	-1.324562	*-0.088*
C22	-4.040648	0.227283	-2.271420	*-0.070*
C23	-3.680031	-0.454977	-3.432119	*-0.067*
C24	-2.345912	-0.807813	-3.635172	*-0.074*
C25	-1.380750	-0.480693	-2.681282	*-0.081*
H26	0.658921	3.288164	1.196025	*+0.038*
H27	2.201670	4.127104	2.929315	*+0.053*
H28	3.291711	2.541200	4.501916	*+0.053*
H29	2.812715	0.110263	4.324057	*+0.051*
H30	1.266568	-0.731066	2.605792	*+0.046*
H31	2.067001	-0.837316	0.168286	*+0.090*
H32	4.014590	-0.944233	-1.351534	*+0.069*
H33	4.131884	0.545276	-3.334037	*+0.068*
H34	2.268087	2.115442	-3.813370	*+0.070*
H35	0.274391	2.156826	-2.349408	*+0.087*
H36	-0.242276	-2.115195	-0.687095	*+0.090*
H37	-0.807632	-4.243374	0.437894	*+0.069*
H38	-1.859474	-4.223656	2.686426	*+0.068*
H39	-2.305145	-2.061477	3.824531	*+0.070*
H40	-1.652805	0.065976	2.743681	*+0.087*
H41	-3.366661	1.081415	-0.421221	*+0.037*
H42	-5.076398	0.510571	-2.106095	*+0.053*
H43	-4.431395	-0.707110	-4.174291	*+0.053*
H44	-2.056445	-1.337670	-4.538670	*+0.051*
H45	-0.345347	-0.760951	-2.868326	*+0.046*

Reference: A. M. Bienfait *et al., Heteroatom Chem.,* 2011, **22**, 576-578.

9. Data for species in Scheme 5

9.1 Molecule 8 (pyridine-*N*-oxide)

Cartesian coordinates and *charges*:

C1	-1.333377	1.035650	0.000445	*-0.022*
C2	0.039856	1.188536	0.000135	*+0.343*
N3	0.875179	0.108694	-0.000246	*-0.564*
C4	0.329446	-1.142737	-0.000211	*+0.343*
C5	-1.039441	-1.330480	0.000134	*-0.022*
C6	-1.900948	-0.236171	0.000431	*-0.028*
O7	2.140929	0.265914	-0.000437	*-0.573*
H8	-1.949562	1.927369	0.000698	*+0.089*
H9	0.553794	2.140572	0.000139	*+0.132*
H10	1.060724	-1.940067	-0.000462	*+0.132*
H11	-1.418732	-2.345856	0.000127	*+0.089*
H12	-2.976182	-0.369743	0.000676	*+0.081*

(well known)

9.2 Molecule 9 (azomethine ylide)

Cartesian coordinates and *charges*:

C1	0.398332	-0.950403	0.900557	*+0.244*
N2	-0.027278	0.093127	-0.048785	*-1.194*
C3	0.953995	0.739232	-0.681419	*+0.239*
C4	-1.344764	0.231716	-0.208784	*+0.240*
H5	-0.345187	-1.046389	1.689673	*+0.066*
H6	1.353360	-0.670465	1.341352	*+0.066*
H7	0.498117	-1.895564	0.367095	*+0.071*
H8	1.978185	0.503561	-0.439594	*+0.065*
H9	0.697852	1.467205	-1.438141	*+0.069*
H10	-2.011561	-0.377259	0.380720	*+0.065*
H11	-1.711187	0.935355	-0.942819	*+0.069*

(well known)

9.3 Anion 10

Cartesian coordinates and *charges*:

N1	-0.076062	-0.394626	0.170928	*-1.243*
C2	1.183641	-0.087564	-0.101616	*+0.232*
C3	-1.126587	0.385152	-0.038270	*+0.232*
H4	1.966243	-0.817005	0.116058	*-0.048*
H5	1.485359	0.876573	-0.543511	*-0.062*
H6	-2.120068	0.019132	0.228107	*-0.048*
H7	-1.048448	1.395038	-0.474032	*-0.062*

Reference: S. R. Kass and C. H. DePuy, *J. Org. Chem.,* 1985, **50**, 2874-2877.

9.4 Molecule 11

Cartesian coordinates and *charges*:

C1	-1.207316	-2.296742	0.000099	*+0.010*
C2	-1.180566	-0.918150	0.000283	*+0.337*
N3	-0.001779	-0.229557	0.000661	*-1.186*
C4	1.166467	-0.935893	0.000826	*+0.337*
C5	1.172372	-2.314737	0.000602	*+0.010*
C6	-0.022925	-3.027104	0.000231	*-0.021*
C7	0.008818	1.174748	0.000861	*+0.531*
C8	1.244397	1.844052	0.001108	*+0.852*
N9	2.296008	2.349695	0.000889	*-1.173*
C10	-1.216522	1.862604	0.000754	*+0.852*
N11	-2.260414	2.383993	0.000817	*-1.173*
H12	-2.174004	-2.785464	-0.000213	*+0.107*
H13	-2.081856	-0.320054	0.000139	*+0.156*
H14	2.076691	-0.351486	0.001151	*+0.156*
H15	2.131564	-2.818012	0.000756	*+0.107*
H16	-0.031120	-4.110363	0.000035	*+0.100*

Reference: C. Bugg and R. L. Sass, *Acta Cryst.,* 1965, **18**, 591-594.

9.5 Molecule 12

Cartesian coordinates and *charges*:

C1	-3.784908	-1.339391	0.237830	*-0.002*
C2	-2.665374	-0.532949	0.233053	*+0.332*
N3	-1.423777	-1.052014	0.002247	*-1.217*
C4	-1.297628	-2.392157	-0.226573	*+0.332*
C5	-2.400325	-3.221490	-0.227384	*-0.002*
C6	-3.671206	-2.706346	0.006261	*-0.038*
C7	-0.292625	-0.219349	0.000194	*+0.197*
C8	-0.320361	1.206486	0.230428	*+0.975*
C9	1.059971	-0.669806	-0.232934	*+0.975*
H10	-4.745557	-0.875446	0.426303	*+0.099*
H11	-2.695596	0.535663	0.407010	*+0.219*
H12	-0.286675	-2.738836	-0.402339	*+0.219*
H13	-2.243217	-4.276927	-0.414424	*+0.099*
H14	-4.544141	-3.348906	0.007799	*+0.093*
O15	-1.273354	1.954755	0.463174	*-1.164*
O16	1.490725	-1.802497	-0.464761	*-1.164*
C17	1.917390	0.560367	-0.139352	*-0.053*
C18	1.108728	1.659590	0.132077	*-0.053*
C19	1.638441	2.928915	0.270966	*-0.028*
C20	3.024730	3.072992	0.128558	*-0.055*
C21	3.836136	1.970043	-0.143797	*-0.055*
C22	3.286394	0.688845	-0.282174	*-0.028*
H23	0.995314	3.777009	0.482688	*+0.094*
H24	3.476949	4.054422	0.230857	*+0.065*
H25	4.907317	2.110115	-0.249269	*+0.065*
H26	3.904599	-0.177595	-0.493853	*+0.094*

Reference: V. F. Kaminskii *et al.*, *J. Struct. Chem.,* 1976, **17**, 768-777.

9.6 Molecule 13

Cartesian coordinates and *charges*:

C1	-1.456115	-0.467883	0.015967	*+0.006*
C2	-1.418289	0.958365	-0.130637	*-0.019*
C3	-0.258246	1.683540	-0.205997	*-0.261*
S4	1.279039	0.931598	0.120818	*+0.417*
C5	0.978728	-0.687595	-0.423369	*-0.095*
C6	-0.314912	-1.204618	-0.252437	*-0.043*
C7	-2.776946	-1.173596	0.220915	*-0.048*
C8	-2.718786	1.703491	-0.311391	*-0.052*
C9	1.283930	0.775682	1.943601	*-0.189*
C10	2.074470	-1.411629	-0.946305	*+0.833*
N11	2.988452	-2.007450	-1.357902	*-1.202*
H12	-0.209178	2.722057	-0.508135	*+0.092*
H13	-0.417828	-2.276923	-0.404871	*+0.085*
H14	-3.487873	-0.975910	-0.588512	*+0.029*
H15	-2.631307	-2.254627	0.270137	*+0.035*
H16	-3.260763	-0.871801	1.155712	*+0.026*
H17	-3.364261	1.561038	0.559722	*+0.050*
H18	-2.553659	2.774327	-0.438228	*+0.039*
H19	-3.263270	1.331517	-1.183418	*+0.052*
H20	0.374451	0.267055	2.262600	*+0.092*
H21	2.169229	0.201262	2.220732	*+0.082*
H22	1.334100	1.782471	2.362064	*+0.073*

Reference: H. Shimizu *et al.*, *Tetrahedron Lett.,* 1990, **31**, 115-118.

9.7 Molecule 14

Cartesian coordinates and *charges*:

S1	0.219415	0.873010	0.667034	*+1.525*
C2	1.536429	0.366835	-0.511712	*-0.202*
C3	2.317900	1.424484	-0.967427	*-0.031*
C4	3.361406	1.169887	-1.853355	*-0.043*
C5	3.625486	-0.136245	-2.264585	*-0.057*
C6	2.849993	-1.190815	-1.785671	*-0.048*
C7	1.799453	-0.943962	-0.902334	*-0.055*
C8	-1.211025	1.019329	-0.479165	*-0.202*
C9	-2.257965	1.784348	0.026233	*-0.031*
C10	-3.394777	1.984708	-0.752424	*-0.043*
C11	-3.471295	1.433950	-2.031251	*-0.057*
C12	-2.408236	0.686827	-2.535990	*-0.048*
C13	-1.268191	0.476910	-1.760669	*-0.055*
C14	-0.155383	-0.743097	1.459895	*-0.202*
C15	0.505920	-0.940814	2.668351	*-0.031*
C16	0.293937	-2.120221	3.377479	*-0.043*
C17	-0.584943	-3.083734	2.883714	*-0.058*
C18	-1.258242	-2.866579	1.682696	*-0.048*
C19	-1.047784	-1.690457	0.963576	*-0.055*
H20	2.111841	2.428864	-0.607424	*+0.121*
H21	3.974255	1.989057	-2.215054	*+0.073*
H22	4.443282	-0.334037	-2.950102	*+0.069*
H23	3.064766	-2.209292	-2.092797	*+0.069*
H24	1.204078	-1.768700	-0.524744	*+0.087*
H25	-2.160688	2.228921	1.013032	*+0.122*
H26	-4.216457	2.578300	-0.365162	*+0.073*
H27	-4.355382	1.596009	-2.639524	*+0.069*
H28	-2.460495	0.270988	-3.537046	*+0.069*
H29	-0.438971	-0.097365	-2.160771	*+0.087*
H30	1.161189	-0.161059	3.047166	*+0.122*
H31	0.807115	-2.283086	4.319612	*+0.073*
H32	-0.753874	-3.999822	3.440542	*+0.069*
H33	-1.954245	-3.608946	1.305566	*+0.069*
H34	-1.581006	-1.521829	0.033776	*+0.086*
N35	0.501349	2.016080	1.553428	*-1.474*

Reference: T. Yoshimura *et al.*, *J. Org. Chem.,* 1997, **61**, 3802-3803.

9.8 Molecule 15

Cartesian coordinates and *charges*:

S1	0.237705	-0.049836	0.435480	*+0.503*
C2	-1.561768	0.231720	0.385403	*-0.178*
C3	-2.499085	-0.733630	0.032340	*-0.029*
C4	-3.849390	-0.393684	0.019560	*-0.044*
C5	-4.253269	0.904242	0.336467	*-0.054*
C6	-3.306725	1.873511	0.647474	*-0.037*
C7	-1.948347	1.549505	0.655668	*-0.005*
C8	-0.832013	2.468634	0.901537	*-0.035*
C9	-0.902881	3.777445	1.392777	*-0.055*
C10	0.275624	4.483006	1.603916	*-0.055*
C11	1.515103	3.880104	1.354435	*-0.064*
C12	1.576747	2.568284	0.890791	*-0.066*
C13	0.395290	1.878134	0.627619	*-0.168*
H14	-2.188412	-1.735364	-0.236674	*+0.105*
H15	-4.585936	-1.143595	-0.249560	*+0.067*
H16	-5.306901	1.163888	0.317874	*+0.066*
H17	-3.621688	2.890827	0.857778	*+0.064*
H18	-1.859116	4.238201	1.623993	*+0.055*
H19	0.235938	5.500543	1.979630	*+0.059*
H20	2.430253	4.437675	1.530345	*+0.059*
H21	2.542086	2.097002	0.716168	*+0.062*
C22	1.150567	-0.145585	-1.138506	*-0.178*
C23	1.459641	0.951404	-1.936133	*-0.029*
C24	2.153107	0.749132	-3.126613	*-0.044*
C25	2.513745	-0.539689	-3.522893	*-0.054*
C26	2.164237	-1.634062	-2.740370	*-0.037*
C27	1.461055	-1.447380	-1.548344	*-0.005*
C28	0.969423	-2.497427	-0.649939	*-0.035*
C29	1.292506	-3.859075	-0.679221	*-0.055*
C30	0.758316	-4.699679	0.289948	*-0.055*
C31	-0.068113	-4.183375	1.296177	*-0.064*
C32	-0.362898	-2.822716	1.332798	*-0.066*
C33	0.128133	-1.987851	0.331155	*-0.168*
H34	1.161532	1.950476	-1.644000	*+0.105*
H35	2.402086	1.601208	-3.750527	*+0.067*
H36	3.048190	-0.690512	-4.455376	*+0.066*
H37	2.414299	-2.637753	-3.069663	*+0.064*
H38	1.962733	-4.259871	-1.434407	*+0.055*
H39	0.995918	-5.758682	0.276752	*+0.059*
H40	-0.473908	-4.847705	2.053360	*+0.059*
H41	-0.989986	-2.422345	2.127120	*+0.062*

Reference: S. Ogawa *et al., J. Chem. Soc., Chem. Commun.,* 1992, 1141-1142.

9.9 Molecule 16

Cartesian coordinates and *charges (QTAIM, NBO, Hirshfeld, Mulliken)*:

S1	0.425592	-0.368704	0.810884	*+0.825*	*+1.221*	*+0.334*	*+0.780*
C2	-1.284462	-0.562676	-0.060205	*-0.160*	*-0.192*	*-0.048*	*-0.122*
C3	-1.613806	-1.462721	-1.069204	*-0.051*	*-0.223*	*-0.056*	*-0.211*
C4	-2.906157	-1.470580	-1.594018	*-0.060*	*-0.191*	*-0.053*	*-0.273*
C5	-3.870357	-0.587112	-1.109481	*-0.056*	*-0.200*	*-0.056*	*-0.260*
C6	-3.541170	0.310386	-0.100455	*-0.052*	*-0.191*	*-0.051*	*-0.359*
C7	-2.245473	0.321343	0.422062	*-0.025*	*-0.073*	*-0.018*	*+0.088*
C8	-1.758765	1.219988	1.481837	*-0.029*	*-0.063*	*-0.013*	*+0.127*
C9	-2.521366	2.199058	2.123952	*-0.052*	*-0.192*	*-0.049*	*-0.369*
C10	-1.941024	3.003656	3.097986	*-0.059*	*-0.200*	*-0.055*	*-0.252*
C11	-0.598499	2.828820	3.429163	*-0.064*	*-0.200*	*-0.055*	*-0.284*
C12	0.162705	1.851033	2.787432	*-0.060*	*-0.229*	*-0.054*	*-0.256*
C13	-0.415674	1.044223	1.810813	*-0.157*	*-0.202*	*-0.041*	*-0.108*
C14	-0.100886	-1.632365	2.149083	*-0.217*	*-0.804*	*-0.145*	*-0.856*
H15	-0.884472	-2.160886	-1.463121	*+0.082*	*+0.218*	*+0.045*	*+0.272*
H16	-3.159519	-2.170739	-2.384255	*+0.059*	*+0.212*	*+0.054*	*+0.234*
H17	-4.875759	-0.599318	-1.518511	*+0.059*	*+0.211*	*+0.054*	*+0.232*
H18	-4.295244	0.995876	0.274371	*+0.053*	*+0.205*	*+0.052*	*+0.221*
H19	-3.566380	2.340632	1.866067	*+0.057*	*+0.208*	*+0.053*	*+0.227*
H20	-2.530747	3.767153	3.595297	*+0.062*	*+0.213*	*+0.055*	*+0.234*
H21	-0.138026	3.456478	4.185907	*+0.060*	*+0.213*	*+0.054*	*+0.236*
H22	1.206144	1.756153	3.058883	*+0.066*	*+0.216*	*+0.051*	*+0.257*
H23	-0.834304	-2.325977	1.737259	*+0.047*	*+0.220*	*+0.035*	*+0.270*
H24	0.768808	-2.175507	2.522068	*+0.032*	*+0.210*	*+0.036*	*+0.251*
H25	-0.552738	-1.079336	2.972629	*+0.054*	*+0.226*	*+0.037*	*+0.311*
C26	0.945664	0.856285	-0.585422	*-0.160*	*-0.192*	*-0.048*	*-0.122*
C27	0.727935	2.230370	-0.618899	*-0.051*	*-0.223*	*-0.056*	*-0.211*
C28	1.170830	2.974181	-1.712633	*-0.060*	*-0.191*	*-0.053*	*-0.273*
C29	1.835256	2.350367	-2.768267	*-0.056*	*-0.200*	*-0.056*	*-0.260*
C30	2.053923	0.978156	-2.733333	*-0.052*	*-0.191*	*-0.051*	*-0.359*
C31	1.606302	0.232442	-1.639935	*-0.025*	*-0.073*	*-0.018*	*+0.088*
C32	1.761561	-1.222457	-1.476430	*-0.029*	*-0.063*	*-0.013*	*+0.127*
C33	2.380125	-2.076802	-2.392903	*-0.052*	*-0.192*	*-0.049*	*-0.369*
C34	2.446129	-3.441432	-2.135326	*-0.059*	*-0.200*	*-0.055*	*-0.252*
C35	1.893421	-3.950867	-0.961609	*-0.064*	*-0.200*	*-0.055*	*-0.284*
C36	1.275591	-3.097235	-0.046780	*-0.060*	*-0.229*	*-0.054*	*-0.256*
C37	1.207244	-1.730057	-0.302512	*-0.157*	*-0.202*	*-0.041*	*-0.108*
C38	2.111015	-0.109224	1.680837	*-0.217*	*-0.804*	*-0.145*	*-0.856*
H39	0.215575	2.740614	0.188360	*+0.082*	*+0.218*	*+0.045*	*+0.272*
H40	0.995901	4.045450	-1.738518	*+0.059*	*+0.212*	*+0.054*	*+0.234*
H41	2.181246	2.933875	-3.615673	*+0.059*	*+0.211*	*+0.054*	*+0.232*
H42	2.572388	0.496739	-3.557013	*+0.053*	*+0.205*	*+0.052*	*+0.221*
H43	2.808515	-1.684155	-3.309916	*+0.057*	*+0.208*	*+0.053*	*+0.227*
H44	2.922953	-4.107166	-2.847685	*+0.062*	*+0.213*	*+0.055*	*+0.234*
H45	1.937851	-5.016002	-0.756265	*+0.060*	*+0.213*	*+0.054*	*+0.236*
H46	0.842759	-3.531359	0.845211	*+0.066*	*+0.216*	*+0.051*	*+0.257*
H47	2.503584	0.879723	1.443198	*+0.047*	*+0.220*	*+0.035*	*+0.270*
H48	2.003623	-0.226575	2.760273	*+0.032*	*+0.210*	*+0.036*	*+0.251*
H49	2.800025	-0.867756	1.309174	*+0.054*	*+0.226*	*+0.037*	*+0.311*

Reference: S. Sato *et al., J. Am. Chem. Soc.,* 2006, **128**, 6778 - 6779.

10. Data for species in Scheme 7

10.1 Molecule 21

Cartesian coordinates and *charges*:

Cl1	-3.185845	-4.223008	-1.732279	*-0.142*
Cl2	3.185837	4.217631	-1.746180	*-0.142*
O3	-2.117658	1.485747	1.399619	*-1.109*
O4	2.117755	-1.481160	1.404429	*-1.109*
C5(*)	0.000056	0.001759	0.988861	*-0.001*
C6	0.000140	0.004202	2.476868	*-0.034*
C7	-1.190482	0.261911	3.177702	*-0.011*
C8	-1.174542	0.276537	4.568982	*-0.027*
C9	0.000334	0.008782	5.264867	*-0.033*
C10	1.175112	-0.261248	4.569702	*-0.027*
C11	1.190859	-0.251172	3.178374	*-0.011*
C12	-2.458396	0.555701	2.414707	*+0.449*
C13	-2.912672	1.595941	0.287285	*+0.387*
C14	-2.429685	2.456640	-0.703291	*-0.031*
C15	-3.111482	2.575143	-1.905442	*-0.052*
C16	-4.288978	1.854111	-2.152107	*-0.011*
C17	-4.763856	1.024714	-1.137595	*-0.043*
C18	-4.093032	0.888515	0.080285	*-0.034*
C19	-5.036732	2.019064	-3.451285	*-0.053*
C20	2.458651	-0.547515	2.416158	*+0.449*
C21	2.912532	-1.595217	0.292320	*+0.387*
C22	2.429281	-2.459174	-0.695244	*-0.031*
C23	3.110869	-2.581813	-1.897142	*-0.052*
C24	4.288376	-1.861769	-2.146431	*-0.011*
C25	4.763532	-1.028988	-1.134777	*-0.043*
C26	4.092959	-0.888610	0.082726	*-0.034*
C27	5.036330	-2.031295	-3.444908	*-0.054*
C28	-0.743769	-1.016714	0.301373	*+0.022*
C29	-1.000648	-2.248677	0.946744	*-0.036*
C30	-1.731041	-3.237314	0.320087	*+0.021*
C31	-2.250523	-2.998078	-0.959103	*-0.002*
C32	-2.035800	-1.783794	-1.610936	*+0.038*
C33	-1.282765	-0.804530	-0.987589	*-0.039*
C34	0.743909	1.017931	0.298023	*+0.023*
C35	1.282929	0.801521	-0.990222	*-0.039*
C36	2.035929	1.778761	-1.616784	*+0.038*
C37	2.250600	2.995197	-0.968963	*-0.001*
C38	1.731133	3.238608	0.309448	*+0.021*
C39	1.000774	2.252010	0.939348	*-0.036*
H40	-2.085714	0.502977	5.113754	*+0.089*
H41	0.000404	0.010566	6.349241	*+0.100*
H42	2.086365	-0.485895	5.115079	*+0.089*
H43	-3.228895	0.972437	3.070533	*+0.074*
H44	-2.846336	-0.370098	1.974409	*+0.065*
H45	-1.520231	3.019617	-0.516316	*+0.081*
H46	-2.724881	3.245942	-2.667729	*+0.074*
H47	-5.684669	0.468674	-1.287986	*+0.074*
H48	-4.514017	0.242835	0.841929	*+0.071*
H49	-5.609197	2.950710	-3.450390	*+0.059*
H50	-5.738634	1.199597	-3.616186	*+0.044*
H51	-4.352729	2.055705	-4.302325	*+0.043*
H52	3.229315	-0.961872	3.073293	*+0.074*
H53	2.846408	0.376760	1.972510	*+0.065*
H54	1.519768	-3.021374	-0.506239	*+0.081*
H55	2.724021	-3.255112	-2.657089	*+0.074*
H56	5.684366	-0.473538	-1.287252	*+0.074*

continued…

10.1 Molecule 21 (continued)

H57	4.514117	-0.240385	0.842113	*+0.071*
H58	5.731536	-1.207602	-3.617072	*+0.044*
H59	4.351999	-2.080391	-4.295022	*+0.043*
H60	5.616332	-2.958244	-3.436839	*+0.059*
H61	-0.578332	-2.436385	1.927697	*+0.120*
H62	-1.903825	-4.190631	0.806181	*+0.121*
H63	-2.473800	-1.601021	-2.585089	*+0.121*
H64	-1.173757	0.155469	-1.477173	*+0.122*
H65	1.174018	-0.160100	-1.476636	*+0.122*
H66	2.473913	1.592794	-2.590341	*+0.121*
H67	1.903908	4.193514	0.792415	*+0.121*
H68	0.578474	2.442906	1.919693	*+0.120*

note: C5(*)is the hypercoordinate center.

Reference: K.-y. Akiba *et al., J. Am. Chem. Soc.,* 2005, **127**, 5893–5901.

10.2 Molecule 22

Cartesian coordinates and *charges*:

O1	-2.291385	0.913363	1.262308	*-1.133*
O2	2.058655	1.619782	-0.862566	*-1.133*
O3	-0.780514	1.996982	-0.916039	*-1.084*
O4	0.158168	2.167441	1.060781	*-1.104*
C5	-2.230975	-0.437484	1.090300	*+0.423*
C6	-3.196639	-1.314679	1.496776	*+0.012*
C7	-3.026980	-2.714885	1.299483	*-0.029*
C8	-1.901886	-3.202533	0.712019	*-0.024*
C9	2.464355	-2.493571	-1.420147	*-0.024*
C10	3.446644	-1.663727	-1.861973	*-0.029*
C11	3.338010	-0.253582	-1.694692	*+0.012*
C12	2.237657	0.288138	-1.092234	*+0.423*
C13	-0.008864	-0.040706	-0.014680	*+0.001*
C14	0.278605	-2.796473	-0.342303	*-0.028*
C15	-1.029212	-0.898973	0.445391	*+0.023*
C16	-0.874468	-2.310960	0.271073	*+0.006*
C17	1.297795	-1.958247	-0.789668	*+0.006*
C18	1.159791	-0.543527	-0.623583	*+0.023*
C19	-3.452215	1.472671	1.869045	*+0.396*
C20	3.057381	2.530051	-1.312462	*+0.396*
C21	-0.999932	3.430004	-0.888074	*+0.338*
C22	0.816852	1.555613	2.203077	*+0.325*
C23(*)	-0.204134	1.429851	0.074483	*+1.495*
H24	-4.101297	-0.963182	1.977154	*+0.089*
H25	-3.810540	-3.386522	1.631579	*+0.096*
H26	-1.766826	-4.268748	0.565323	*+0.088*
H27	2.546362	-3.568411	-1.540850	*+0.088*
H28	4.330934	-2.064551	-2.344386	*+0.096*
H29	4.144430	0.375780	-2.050093	*+0.089*
H30	0.385412	-3.868835	-0.480144	*+0.093*
H31	-3.280129	2.546405	1.914510	*+0.070*
H32	-3.585992	1.085915	2.883004	*+0.069*
H33	-4.340671	1.267936	1.265939	*+0.072*
H34	2.716386	3.520626	-1.017378	*+0.070*
H35	3.159937	2.485316	-2.399884	*+0.072*
H36	4.017856	2.322008	-0.833086	*+0.069*
H37	-1.495455	3.651960	-1.827296	*+0.117*
H38	-0.040238	3.939466	-0.818146	*+0.099*
H39	-1.633550	3.680346	-0.038776	*+0.099*
H40	0.145144	0.827701	2.654517	*+0.110*
H41	1.011342	2.379329	2.882973	*+0.112*
H42	1.746143	1.094466	1.873449	*+0.110*

note: C23(*)is the hypercoordinate center.

Reference: K.-y. Akiba *et al., J. Am. Chem. Soc.,* 1999*,* **121**, 10644–10645.

10.3 Molecule 23

Cartesian coordinates and *charges*:

S1	-3.871101	-0.486221	-1.426093	*+0.393*
S2	3.871016	0.485711	-1.426251	*+0.393*
O3	-1.026296	0.901317	2.536368	*-1.112*
O4	0.650256	-2.541072	-0.308233	*-1.120*
O5	1.026298	-0.900506	2.536747	*-1.112*
O6	-0.650080	2.541181	-0.308533	*-1.121*
C7	-2.259994	0.833323	2.022621	*+0.552*
C8	-3.388468	1.389574	2.638051	*-0.022*
C9	-4.643234	1.308815	2.046424	*-0.001*
C10	-4.794767	0.688759	0.812462	*-0.005*
C11	-3.023802	-2.917540	-2.234256	*+0.001*
C12	-2.090003	-3.941937	-2.326698	*+0.006*
C13	-0.861926	-3.828173	-1.686409	*-0.016*
C14	-0.537592	-2.693884	-0.933727	*+0.515*
C15	-1.228426	-0.462843	0.079741	*+0.243*
C16	-3.659399	0.147672	0.217652	*-0.215*
C17	-2.681634	-1.798217	-1.484418	*-0.211*
C18	-1.471494	-1.639398	-0.801587	*+0.050*
C19	-2.381147	0.160852	0.784902	*+0.057*
C20	-0.803523	1.604785	3.753344	*+0.393*
C21	1.532644	-3.657026	-0.205885	*+0.386*
C22	-3.081812	0.811553	-2.425395	*-0.170*
C23(*)	-0.000004	0.000040	0.183741	*-0.416*
C24	2.259965	-0.832773	2.022891	*+0.552*
C25	3.388416	-1.388928	2.638448	*-0.022*
C26	4.643151	-1.308483	2.046711	*-0.001*
C27	4.794677	-0.688818	0.812554	*-0.005*
C28	3.023904	2.916994	-2.234853	*+0.001*
C29	2.090175	3.941447	-2.327437	*+0.006*
C30	0.862118	3.827919	-1.687073	*-0.016*
C31	0.537718	2.693795	-0.934157	*+0.515*
C32	1.228419	0.462878	0.079643	*+0.243*
C33	3.659333	-0.147790	0.217633	*-0.215*
C34	2.681666	1.797841	-1.484803	*-0.211*
C35	1.471542	1.639264	-0.801893	*+0.050*
C36	2.381117	-0.160690	0.784967	*+0.057*
C37	0.803551	-1.603920	3.753757	*+0.393*
C38	-1.532573	3.657090	-0.206694	*+0.386*
C39	3.081485	-0.812150	-2.425271	*-0.170*
H40	-3.293818	1.886785	3.595462	*+0.107*
H41	-5.503980	1.736757	2.547271	*+0.121*
H42	-5.765016	0.629758	0.331472	*+0.113*
H43	-3.987107	-2.994182	-2.726583	*+0.116*
H44	-2.318433	-4.834565	-2.897411	*+0.123*
H45	-0.154248	-4.643295	-1.773267	*+0.105*
H46	-1.083814	2.657633	3.657002	*+0.066*
H47	0.266830	1.532184	3.933965	*+0.087*
H48	-1.346120	1.145060	4.584303	*+0.066*
H49	1.039237	-4.509431	0.268484	*+0.079*
H50	2.356105	-3.318869	0.421906	*+0.082*
H51	1.920995	-3.948059	-1.186437	*+0.056*
H52	-3.745189	1.675709	-2.397968	*+0.118*
H53	-2.999782	0.430202	-3.443079	*+0.122*
H54	-2.104481	1.049836	-2.005524	*+0.124*
H55	3.293756	-1.885773	3.596049	*+0.107*
H56	5.503892	-1.736348	2.547634	*+0.121*
H57	5.764919	-0.630035	0.331522	*+0.113*

continued…

10.3 Molecule 23 (continued)

H58	3.987202	2.993489	-2.727217	*+0.116*
H59	2.318661	4.833941	-2.898339	*+0.123*
H60	0.154524	4.643099	-1.774057	*+0.105*
H61	1.083991	-2.656744	3.657526	*+0.066*
H62	-0.266824	-1.531468	3.934312	*+0.087*
H63	1.346081	-1.144066	4.584692	*+0.066*
H64	-1.039297	4.509739	0.267369	*+0.079*
H65	-2.356040	3.319121	0.421188	*+0.082*
H66	-1.920882	3.947674	-1.187399	*+0.056*
H67	2.104244	-1.050385	-2.005156	*+0.124*
H68	3.744848	-1.676318	-2.397955	*+0.118*
H69	2.999214	-0.430848	-3.442956	*+0.122*

note: C23(*)is the hypercoordinate center.

Reference: T. Yamaguchi *et al.*, *J. Am. Chem. Soc.,* 2008, **130**, 6894–6895.

10.4 Molecule 24
Cartesian coordinates and *charges*:

S1	4.229856	-0.199437	-0.690635	*+0.393*
S2	-4.229845	0.199731	-0.690428	*+0.392*
H3	0.634968	-1.043071	2.547470	*+0.092*
H4	0.156666	2.528916	-0.590019	*+0.089*
H5	-0.634670	1.042334	2.547622	*+0.092*
H6	-0.156961	-2.529102	-0.590485	*+0.089*
C7	1.680999	-1.172898	2.288789	*-0.014*
C8	2.511886	-1.900780	3.135198	*-0.014*
C9	3.863519	-2.062874	2.839113	*+0.006*
C10	4.383009	-1.505597	1.675292	*-0.009*
C11	3.845105	2.263095	-1.751911	*-0.008*
C12	3.090837	3.384636	-2.081331	*+0.006*
C13	1.760067	3.462423	-1.675956	*-0.017*
C14	1.178883	2.431970	-0.942799	*-0.011*
C15	1.300843	0.205897	0.213237	*+0.211*
C16	3.531240	-0.797704	0.830424	*-0.207*
C17	3.239327	1.233580	-1.036339	*-0.207*
C18	1.905721	1.285155	-0.613290	*+0.010*
C19	2.173832	-0.598460	1.114210	*+0.003*
C20	3.615117	-1.402325	-1.907453	*-0.159*
C21	0.000026	-0.000184	0.195474	*-0.528*
C22	-1.680704	1.172327	2.289033	*-0.014*
C23	-2.511458	1.900124	3.135643	*-0.014*
C24	-3.863091	2.062436	2.839671	*+0.006*
C25	-4.382714	1.505462	1.675763	*-0.009*
C26	-3.845447	-2.262684	-1.752099	*-0.008*
C27	-3.091335	-3.384270	-2.081721	*+0.006*
C28	-1.760547	-3.462270	-1.676440	*-0.017*
C29	-1.179190	-2.431987	-0.943183	*-0.012*
C30	-1.300827	-0.206040	0.213146	*+0.211*
C31	-3.531073	0.797651	0.830697	*-0.207*
C32	-3.239498	-1.233339	-1.036424	*-0.207*
C33	-1.905869	-1.285127	-0.613478	*+0.010*
C34	-2.173672	0.598200	1.114361	*+0.003*
C35	-3.615029	1.402755	-1.907072	*-0.159*
H36	2.103154	-2.333322	4.041580	*+0.121*
H37	4.511900	-2.615877	3.508584	*+0.129*
H38	5.434855	-1.623203	1.434380	*+0.120*
H39	4.888048	2.197718	-2.045439	*+0.121*
H40	3.546494	4.197797	-2.634158	*+0.128*
H41	1.177543	4.345722	-1.913316	*+0.116*
H42	4.102219	-2.353532	-1.695043	*+0.128*
H43	3.908135	-1.039749	-2.892363	*+0.130*
H44	2.532521	-1.484856	-1.825576	*+0.106*
H45	-2.102622	2.332429	4.042091	*+0.121*
H46	-4.511368	2.615374	3.509296	*+0.129*
H47	-5.434563	1.623225	1.434941	*+0.120*
H48	-4.888405	-2.197144	-2.045540	*+0.121*
H49	-3.547124	-4.197306	-2.634624	*+0.128*
H50	-1.178144	-4.345607	-1.913956	*+0.116*
H51	-2.532421	1.485157	-1.825227	*+0.106*
H52	-4.102023	2.353978	-1.694482	*+0.128*
H53	-3.908126	1.040376	-2.892031	*+0.130*

(fragment of molecule **23**)

10.5 Molecule 25

Cartesian coordinates and *charges*:

S1	-0.473315	-2.163594	-0.269160	*+0.366*
O2	-1.845262	2.678335	0.095469	*-1.111*
O3	2.702982	1.756634	-0.221877	*-1.111*
C4	-2.224048	1.390085	0.318214	*+0.453*
C5	-3.473706	1.011706	0.756429	*-0.034*
C6	-3.792060	-0.350484	0.983156	*-0.031*
C7	-2.851074	-1.319149	0.731696	*-0.057*
C8	2.156315	-2.333897	0.382318	*-0.057*
C9	3.423549	-1.812747	0.479704	*-0.030*
C10	3.630763	-0.428011	0.260742	*-0.034*
C11	2.579037	0.416745	-0.016874	*+0.453*
C12	0.132568	0.815744	-0.238583	*-0.032*
C13	-1.583820	-0.925421	0.259416	*-0.221*
C14	1.089911	-1.467244	0.072876	*-0.220*
C15	1.223246	-0.065859	-0.089798	*-0.003*
C16	-1.184102	0.421979	0.078142	*-0.003*
C17	-2.796467	3.697311	0.311765	*+0.433*
C18	3.994287	2.321239	-0.161595	*+0.433*
C19	-0.572918	-2.026670	-2.093743	*-0.188*
H20	-4.237043	1.757049	0.948024	*+0.056*
H21	-4.780392	-0.620046	1.337897	*+0.057*
H22	-3.079777	-2.371484	0.872461	*+0.061*
H23	1.975918	-3.396023	0.519709	*+0.061*
H24	4.267950	-2.453715	0.706531	*+0.057*
H25	4.641442	-0.042189	0.328558	*+0.056*
H26	-3.665245	3.580893	-0.346842	*+0.039*
H27	-2.292028	4.633816	0.077811	*+0.061*
H28	-3.131514	3.719622	1.355312	*+0.041*
H29	4.447540	2.183538	0.827014	*+0.041*
H30	3.865787	3.386032	-0.351163	*+0.061*
H31	4.653998	1.895369	-0.926778	*+0.039*
H32	-1.575446	-2.338226	-2.389327	*+0.076*
H33	0.181564	-2.694027	-2.511948	*+0.076*
H34	-0.383724	-0.992932	-2.382541	*+0.111*
H35	0.337655	1.868897	-0.358978	*+0.105*

(fragment of molecule **23**)

11. Miscellaneous species mentioned in the text

11.1 S_3

Geometry: bent, S-S-S 117.0º S-S 1.925 Å
Charges: S(terminal) -0.099 S(central) +0.198
Reference: M. C. McCarthy *et al., J. Am. Chem. Soc.,* 2004, **126**, 4096–4097.

11.2 S_N2 intermediate $Cl\text{-}CH_3\text{-}Cl$

Cartesian coordinates and *charges*:

Cl1	0.000000	0.000000	0.150292	*-0.712*
C2	0.000000	0.000000	2.493303	*+0.025*
H3	1.074891	0.000000	2.493303	*+0.133*
H4	-0.537445	0.930883	2.493303	*+0.133*
H5	-0.537446	-0.930883	2.493303	*+0.133*
Cl6	0.000000	0.000000	4.836315	*-0.712*

Single imaginary frequency = -408.9 cm^{-1}

11.3 S_N2 intermediate $Br\text{-}CH_3\text{-}Br$

Cartesian coordinates and *charges*:

Br1	0.000000	0.000000	0.000000	*-0.687*
C2	0.000000	0.000000	2.493303	*-0.036*
H3	1.075201	0.000000	2.493303	*+0.137*
H4	-0.537600	0.931151	2.493303	*+0.137*
H5	-0.537600	-0.931151	2.493303	*+0.137*
Br6	0.000000	0.000000	4.986607	*-0.687*

Single imaginary frequency = -400.4 cm^{-1}

11.4 CH_3Cl

Cartesian coordinates and *charges*:

Cl1	0.000256	-0.002026	-0.010653	*-0.269*
C2	-0.001992	0.000420	1.779137	*+0.036*
H3	1.028230	-0.005295	2.127875	*+0.078*
H4	-0.512438	0.896504	2.124833	*+0.078*
H5	-0.522797	-0.888887	2.126835	*+0.078*

11.5 CH_3Br

Cartesian coordinates and *charges*:

Br1	0.000261	-0.001800	0.030194	*-0.151*
C2	-0.001850	0.000427	1.977799	*-0.100*
H3	1.031036	-0.005376	2.314555	*+0.084*
H4	-0.513879	0.898399	2.311696	*+0.084*
H5	-0.524309	-0.890933	2.313783	*+0.084*

11.6 SiH_6

Geometry: octahedral, Si-H 1.656 Å
Charges: Si +2.609 H -0.768

11.7 Ph_3SiH_2

Cartesian coordinates and *charges*:

Si1	0.000821	-0.000377	0.000002	*+2.717*
H2	0.001140	-0.000388	-1.582160	*-0.694*
H3	0.000847	-0.000425	1.582163	*-0.694*
C4	1.364380	-1.393133	0.000111	*-0.494*
C5	1.878847	-1.918416	1.193995	*-0.087*
C6	2.855573	-2.916199	1.203966	*-0.097*
C7	3.349321	-3.420890	0.000266	*-0.097*
C8	2.855375	-2.916578	-1.203511	*-0.097*
C9	1.878669	-1.918773	-1.193692	*-0.087*
C10	0.524454	1.876996	0.000072	*-0.494*
C11	0.721924	2.585250	-1.193789	*-0.087*
C12	1.096478	3.930332	-1.203644	*-0.097*
C13	1.285629	4.610453	0.000170	*-0.096*
C14	1.096107	3.930349	1.203935	*-0.097*
C15	0.721562	2.585265	1.193984	*-0.087*
C16	-1.887111	-0.484571	-0.000182	*-0.494*
C17	-2.599475	-0.667061	1.193624	*-0.087*
C18	-3.952143	-1.013215	1.203392	*-0.097*
C19	-4.636018	-1.188044	-0.000452	*-0.096*
C20	-3.951979	-1.012933	-1.204162	*-0.097*
C21	-2.599310	-0.666791	-1.194128	*-0.087*
H22	1.495766	-1.526879	2.135030	*+0.059*
H23	3.233029	-3.301546	2.149025	*+0.022*
H24	4.110199	-4.198181	0.000326	*+0.021*
H25	3.232666	-3.302229	-2.148511	*+0.022*
H26	1.495462	-1.527503	-2.134787	*+0.059*
H27	0.575047	2.057387	-2.134746	*+0.059*
H28	1.241354	4.450047	-2.148639	*+0.022*
H29	1.577368	5.658310	0.000208	*+0.021*
H30	1.240691	4.450079	2.148967	*+0.022*
H31	0.574406	2.057414	2.134905	*+0.059*
H32	-2.068786	-0.531322	2.134654	*+0.059*
H33	-4.474849	-1.147079	2.148360	*+0.022*
H34	-5.689789	-1.457635	-0.000555	*+0.021*
H35	-4.474558	-1.146572	-2.149232	*+0.022*
H36	-2.068488	-0.530849	-2.135055	*+0.059*

11.8 OF_4

Geometry: seesaw, F-O-F (ax-eq) 94.9°, (eq-eq) 104.3°,
O-F (ax) 1.760 Å, O-F (eq) 1.378 Å

Thermodynamics: H -474.000859 Hartree S 73.522 cal mol^{-1} K^{-1}

Charges: O +0.614 F (ax) -0.322 F (eq) +0.014

12. Worked examples of γ calculations

12.1 $[XF_6]^-$ species (X = Cl, Br or I)

Contributing resonance forms:

(A) 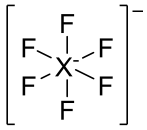

fully covalent
(X has 14 e)

(B) 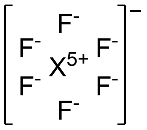

fully ionic
(X has 2e)

For X = Cl, QTAIM charges: Cl +1.904, F -0.484.
This is reproduced by [0.516 x **(A)**] + [0.484 x **(B)**]
Such that charge on Cl = (0.516 x -1) + (0.484 x +5) = +1.904
Then γ(Cl) = (0.516 x 14) + (0.484 x 2) = 8.192.

For X = Br, QTAIM charges: Br +2.456, F -0.576
This is reproduced by [0.424 x **(A)**] + [0.576 x **(B)**]
Such that charge on Br = (0.424 x -1) + (0.576 x +5) = +2.456
Then γ(Br) = (0.424 x 14) + (0.576 x 2) =7.088.

For X = I, QTAIM charges: I +3.032, F -0.672
This is reproduced by [0.328 x **(A)**] + [0.672 x **(B)**]
Such that charge on I = (0.328 x -1) + (0.672 x +5) = +3.032
Then γ(I) = (0.328 x 14) + (0.672 x 2) = 5.936.

12.2 XCl_4 species (X = S, Te)

Contributing resonance forms:

(A) 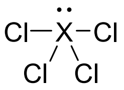

fully covalent
(X has 10 e)

(B) 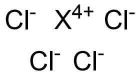

fully ionic
(X has 2 e)

For X = S, QTAIM charges: S +0.833, Cl(ax) -0.369, Cl(eq) -0.048
This is reproduced by [0.7918 x **(A)**] + [0.2083 x **(B)**]
Such that charge on S = (0.7918 x 0) + (0.2083 x +4) = +0.833
Then γ(S) = (0.7918 x 10) + (0.2083 x 2) = 8.335.

For X = Te, QTAIM charges: Te +1.713, Cl(ax) -0.509, Cl(eq) -0.348
This is reproduced by [0.5718 x **(A)**] + [0.4283 x **(B)**]
Such that charge on Te = (0.5718 x 0) + (0.4283 x +4) = +1.713
Then γ(Te) = (0.5718 x 10) + (0.4283 x 2) = 6.575.

12.3 XeO_4

Contributing resonance forms:

(A)

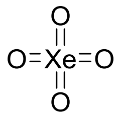

fully covalent
(Xe has 16 e)

(B)

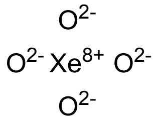

fully ionic
(Xe has 0 e)

QTAIM charges: Xe +3.140, O -0.785
This is reproduced by [0.6075 x **(A)**] + [0.3925 x **(B)**]
Such that charge on Xe = (0.6075 x 0) + (0.3925 x +8) = +3.140
Then γ(Xe) = (0.6075 x 16) + (0.9325 x 0) = 9.720.

12.4 $[XO_4]^{3-}$ species (X = N, P, As)

These were treated as $[Rb_2XO_4]^-$; contributing resonance forms:

(A)

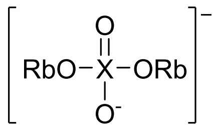

fully covalent
(X has 10 e)

(B)

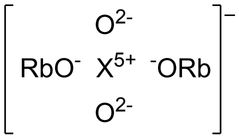

fully ionic
(X has 0 e)

For X = N, QTAIM charges: N +0.749, O -0.934 (x 2), -0.808 (x 2), Rb +0.868 (x 2)
This is reproduced by [0.8502 x **(A)**] + [0.1498 x **(B)**]
Such that charge on N = (0.8502 x 0) + (0.1498 x +5) = +0.749
Then γ(N) = (0.8502 x 10) + (0.1498 x 0) = 8.502.

For X = P, QTAIM charges: P +3.701, O -1.637 (x 2), -1.615 (x 2), Rb +0.900 (x 2)
This is reproduced by [0.2598 x **(A)**] + [0.7402 x **(B)**]
Such that charge on P = (0.2598 x 0) + (0.7402 x +5) = +3.701
Then γ(P) = (0.2598 x 10) + (0.7402 x 0) = 2.598.

For X = As, QTAIM charges: As +2.585, O -1.388 (x 2), -1.301 (x 2), Rb +0.896 (x 2)
This is reproduced by [0.483 x **(A)**] + [0.517 x **(B)**]
Such that charge on As = (0.483 x 0) + (0.517 x +5) = +2.585
Then γ(As) = (0.483 x 10) + (0.517 x 0) = 4.830.

12.5 RNO_2 species (R = Cl, CF_3, MeO, Me, O_2N)

Contributing resonance forms:

(A)

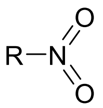

fully covalent
(N has 10 e)

(B)

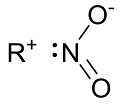

partially ionic
(N has 8 e)

(C)

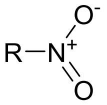

partially ionic
(N has 8 e)

(D)

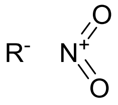

partially ionic
(N has 8 e)

For R = Cl, charges: Cl +0.020, N +0.703, O -0.361 (x2).
This is reproduced by [0.277 x **(A)**] + [0.020 x **(B)**] + [0.703 x **(C)**]
Such that charge on Cl = (0.277 x 0) + (0.020 x +1) + (0.703 x 0) = +0.020
Charge on N = (0.277 x 0) + (0.020 x 0) + (0.703 x +4) = +0.703
Charge on O = (0.277 x 0) + (0.020 x -0.5) + (0.703 x -0.5) = -0.362
Then γ(N) = (0.277 x 10) + (0.020 x 8) + (0.703 x 8) = 8.554.

For R = CF_3, charges: CF_3 +0.294, N +0.529, O -0.408, -0.414 (average -0.411)
This is reproduced by [0.177 x **(A)**] + [0.294 x **(B)**] + [0.529 x **(C)**]
Such that charge on CF_3 = (0.177 x 0) + (0.294 x +1) + (0.529 x 0) = +0.294
Charge on N = (0.177 x 0) + (0.294 x 0) + (0.529 x +1) = +0.529
Charge on O = (0.177 x 0) + (0.294 x -0.5) + (0.529 x -0.5) = -0.412
Then γ(N) = (0.177 x 10) + (0.294 x 8) + (0.529 x 8) = 8.354.

For R = MeO, charges: MeO -0.023, N +0.872, O -0.407, -0.443 (average -0.425)
This is reproduced by [0.128 x **(A)**] + [0.849 x **(C)**] + [0.023 x **(D)**]
Such that charge on MeO = (0.128 x 0) + (0.849 x 0) + (0.023 x -1) = -0.023
Charge on N = (0.128 x 0) + (0.849 x +1) + (0.023 x +1) = +0.872
Charge on O = (0.128 x 0) + (0.849 x -0.5) + (0.023 x 0) = -0.425
Then γ(N) = (0.128 x 10) + (0.849 x 8) + (0.023 x 8) = 8.256.

For R = Me, charges: Me +0.506, N +0.427, O -0.465, -0.468 (average -0.467)
This is reproduced by [0.067 x **(A)**] + [0.506 x **(B)**] + [0.427 x **(C)**]
Such that charge on Me = (0.067 x 0) + (0.506 x +1) + (0.427 x 0) = +0.506
Charge on N = (0.067 x 0) + (0.506 x 0) + (0.427 x +1) = +0.427
Charge on O = (0.067 x 0) + (0.506 x -0.5) + (0.427 x -0.5) = -0.467
Then γ(N) = (0.067 x 10) + (0.506 x 8) + (0.427 x 8) = 8.134.

For R = O_2N, charges: N +0.742, O -0.371
This is reproduced by [0.258 x **(A)**] + [0.742 x **(C)**]
Such that charge on N = (0.258 x 0) + (0.742 x +1) = +0.742
Charge on O = (0.258 x 0) + (0.742 x -0.5) = -0.371
Then γ(N) = (0.258 x 10) + (0.742 x 8) = 8.516.

12.6 HXF species (X = Ar, Kr, Xe)

Contributing resonance forms:

(A)	(B)	(C)	(D)
H–X–F	H^{+} ^{-}X–F	H^{-} ^{+}X–F	H–X^{+} F^{-}
fully covalent (X has 10 e)	*partially ionic (X has 10 e)*	*partially ionic (X has 8 e)*	*partially ionic (X has 8 e)*

For X = Ar, charges: H +0.255, Ar +0.429, F -0.684
This is reproduced by [0.061 x **(A)**] + [0.255 x **(B)**] + [0.684 x **(D)**
Such that charge on Ar = (0.061 x 0) + (0.255 x -1) + (0.684 x +1) = +0.429
Then γ(Ar) = (0.061 x 10) + (0.255 x 10) + (0.684 x 8) = 8.632.

For X = Kr, charges: H +0.131, Kr +0.578, F -0.709
This is reproduced by [0.160 x **(A)**] + [0.131 x **(B)**] + [0.709 x **(D)**
Such that charge on Kr = (0.160 x 0) + (0.131 x -1) + (0.709 x +1) = +0.578
Then γ(Kr) = (0.160 x 10) + (0.131 x 10) + (0.709 x 8) = 8.582.

For X = Xe, charges: H -0.062, Xe +0.805, F -0.743
This is reproduced by [0.195 x **(A)**] + [0.062 x **(C)**] + [0.743 x **(D)**
Such that charge on Xe = (0.195 x 0) + (0.062 x +1) + (0.743 x +1) = +0.805
Then γ(Xe) = (0.195 x 10) + (0.062 x 8) + (0.743 x 8) = 8.390.

12.7 Molecule 8 (Scheme 5)

Summary of charge map:

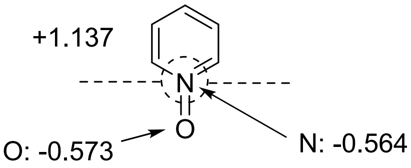

Three contributing resonance forms are required to reproduce this distribution (note that as the most electronegative atom, the O must have 8 electrons in all valid resonance forms):

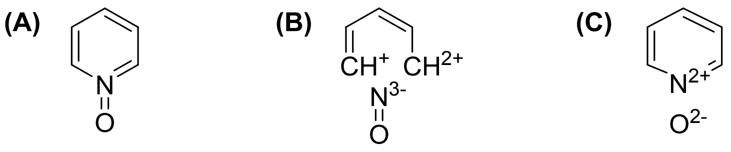

(N has 10 e) *(N has 10 e)* *(N has 6 e)*

Proportions: [0.3345 x **(A)**] + [0.3790 x **(B)**] + [0.2865 x **(C)**]
Such that charge on N = (0.3345 x 0) + (0.3790 x -3) + (0.2865 x +2) = -0.564
Then γ(N) = (0.3345 x 10) + (0.3790 x 10) + (0.2865 x 6) = 8.854.

12.8 Molecule 11 (Scheme 5)

Summary of charge map:

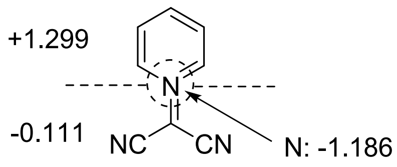

Three contributing resonance forms are required to reproduce this distribution:

(A)

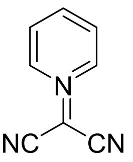

(N has 10 e)

(B)

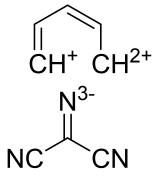

(N has 10 e)

(C)

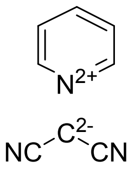

(N has 6 e)

Proportions: [0.5115 x **(A)**] + [0.4330 x **(B)**] + [0.0555 x **(C)**]
Such that charge on N = (0.5115 x 0) + (0.4330 x -3) + (0.0555 x +2) = -1.188
Then γ(N) = (0.5115 x 10) + (0.4330 x 10) + (0.0555 x 6) = 9.778.

12.9 Molecule 13 (Scheme 5)

Summary of charge map:

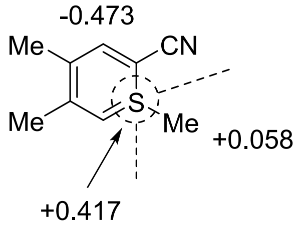

Three contributing resonance forms are required to reproduce this distribution:

(A)

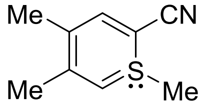

fully covalent
(S has 10 e)

(B)

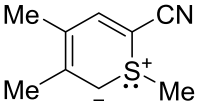

partially ionic
(S has 8 e)

(C)

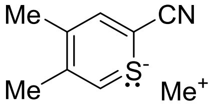

partially ionic
(S has 10 e)

Proportions: [0.469 x **(A)**] + [0.473 x **(B)**] + [0.057 x **(C)**]
Such that charge on S = (0.469 x 0) + (0.473 x +1) + (0.057 x -1) = +0.416
Then γ(S) = (0.469 x 10) + (0.473 x 8) + (0.057 x 10) = 9.044.

12.10 Molecule 14 (Scheme 5)

Summary of charge map: S +1.525, N -1.474, Ph -0.017 (x 3)
Three contributing resonance forms are required to reproduce this distribution:

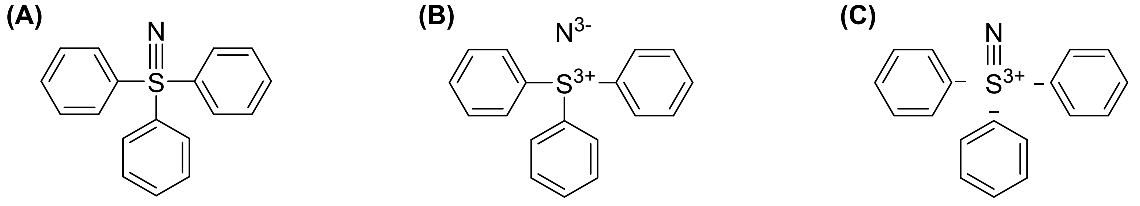

fully covalent	*partially ionic*	*partially ionic*
(S has 12 e)	*(S has 6 e)*	*(S has 6 e)*

Proportions: [0.4917 x **(A)**] + [0.4913 x **(B)**] + [0.017 x **(C)**]
Such that charge on S = (0.4917 x 0) + (0.4913 x +3) + (0.017 x +3) = +1.525
Then γ(S) = (0.4917 x 12) + (0.4913 x 6) + (0.017 x 6) = 8.950.

12.11 Molecule 16 (Scheme 5)

Summary of charge map:

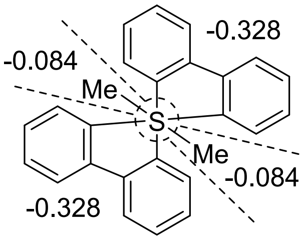

charge on S = +0.825

Three contributing resonance forms are required to reproduce this distribution:

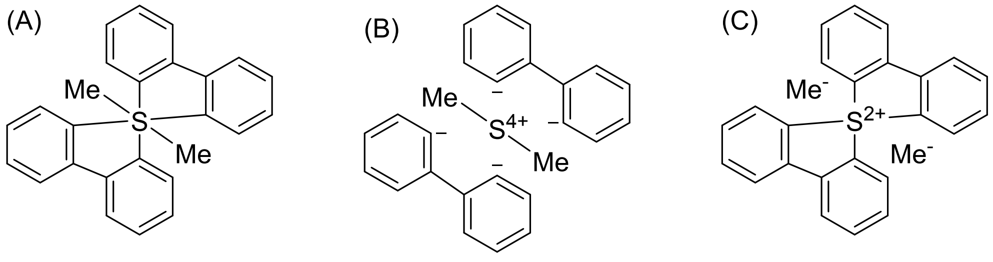

fully covalent	*partially ionic*	*partially ionic*
(S has 12 e)	*(S has 4 e)*	*(S has 8 e)*

Proportions: [0.752 x **(A)**] + [0.164 x **(B)**] + [0.084 x **(C)**]
Such that charge on S = (0.752 x 0) + (0.164 x +4) + (0.084 x +2) = +0.824
Then γ(S) = (0.752 x 12) + (0.164 x 4) + (0.084 x 8) = 10.352.

12.12 Molecule 21 (Scheme 7)

Summary of charge map:

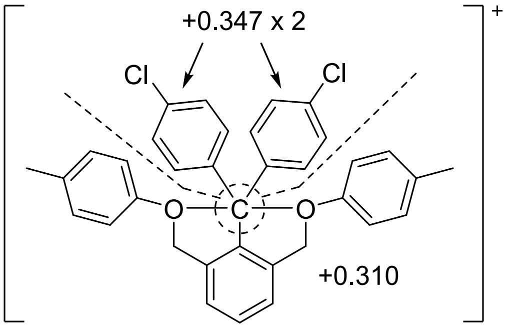

charge on central C = -0.001

Three contributing resonance forms are required to reproduce this distribution:

(A)

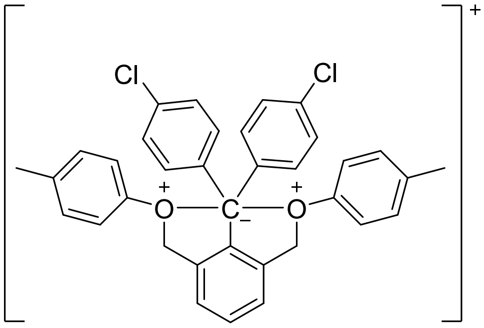

(C has 10 e)

(B)

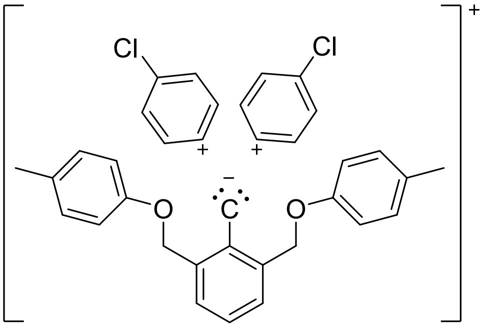

(C has 6 e)

(C)

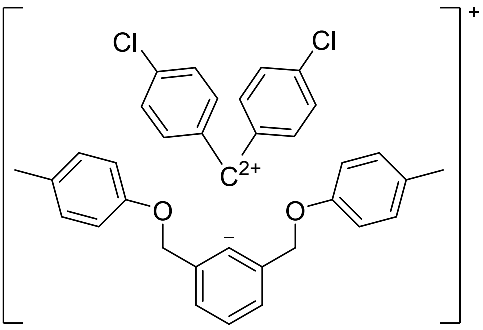

(C has 4 e)

Proportions: [0.320 x **(A)**] + [0.347 x **(B)**] + [0.333 x **(C)**]
Such that charge on hypercoordinate C

$$= (0.320 \times -1) + (0.347 \times -1) + (0.333 \times +2) = -0.001$$

Then γ(C) = (0.320 x 10) + (0.347 x 6) + (0.333 x 4) = 6.614.

12.13 Molecule 22 (Scheme 7)

Summary of charge map:

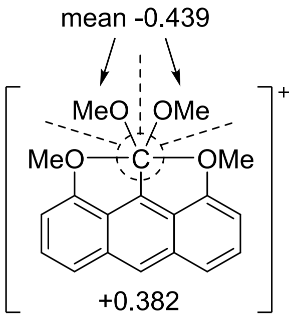

charge on hypercoordinate C = +1.495

Three contributing resonance forms are required to reproduce this distribution:

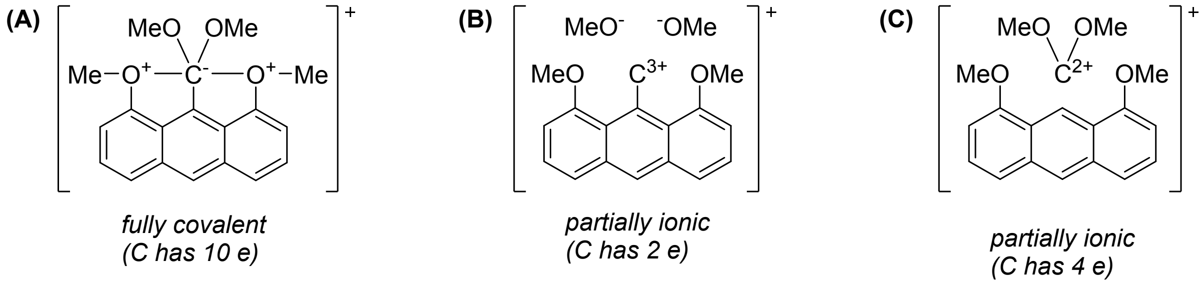

Proportions: [0.3143 x **(A)**] + [0.439 x **(B)**] + [0.2467 x **(C)**]
Such that charge on hypercoordinate C =
(0.3143 x -1) + (0.439 x +3) + (0.2467 x +2) = +1.496
Then γ(C) = (0.3143 x 10) + (0.439 x 2) + (0.2467 x 4) = 5.008.

12.14 Molecule 23 (Scheme 7)

Summary of charge map:

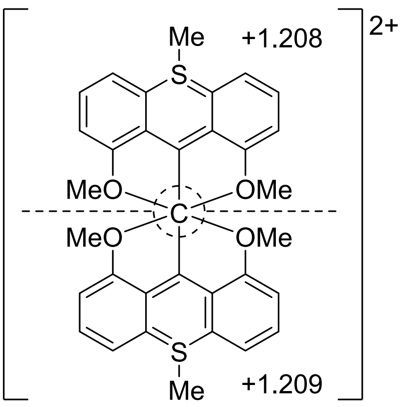

charge on hypercoordinate C = -0.416

If the hypervalent form of the structure is assumed, then two contributing resonance forms are required to reproduce this distribution:

(A)

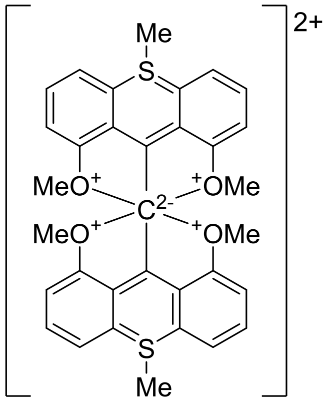

(C has 12 e)

(B)

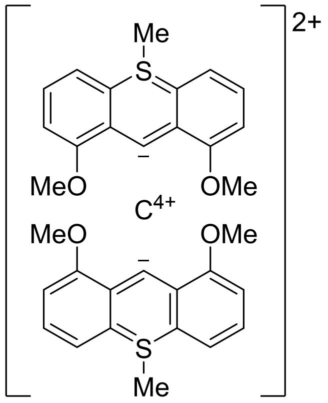

(C has 0 e)

Proportions: [0.73617 x **(A)**] + [0.26383 x **(B)**]
Such that charge on hypercoordinate C = (0.73617 x -2) + (0.26383 x +4) = -0.417
Then γ(C) = (0.73617 x 12) + (0.26383 x 0) = 8.834.

13. Plots of γ(X) versus ΔG(X-O) for different charge schemes (see Table 4)

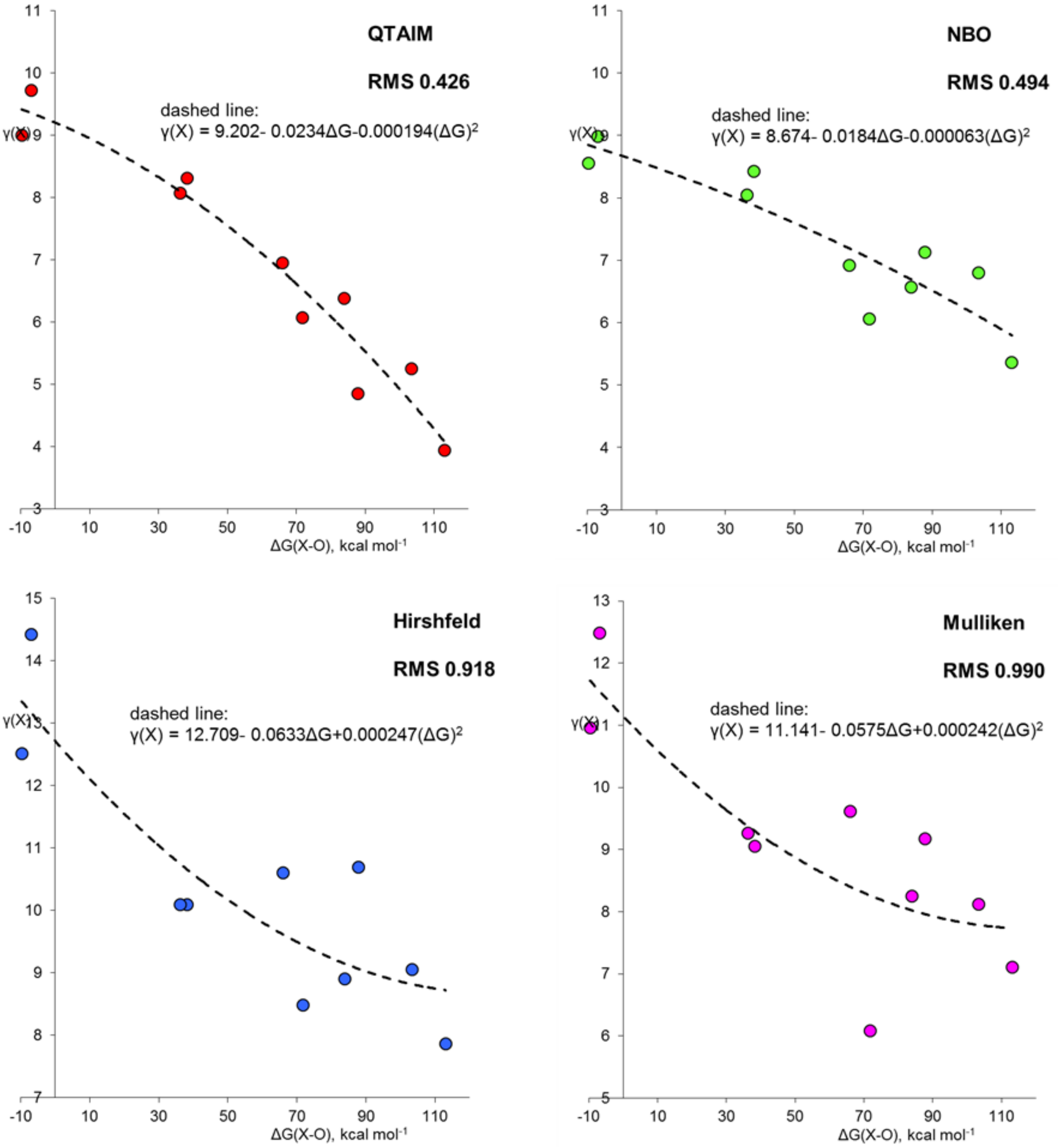

Raw data:

species	ΔG(X-O)	γ(AIM)	γ(NBO)	γ(Hirshfeld)	γ(Mulliken)
PO2	113.105	3.94	5.36	7.86	7.11
SO2	103.385	5.25	6.80	9.05	8.12
SeO2	83.905	6.38	6.57	8.90	8.25
TeO2	71.83	6.07	6.06	8.48	6.08
ClO2	38.23	8.31	8.42	10.09	9.05
BrO2	36.275	8.07	8.05	10.09	9.26
SO3	87.81	4.85	7.13	10.69	9.17
SeO3	65.96	6.95	6.92	10.60	9.62
XeO3	-9.69	9.00	8.55	12.51	10.96
XeO4	-6.8925	9.72	8.98	14.42	12.49
